# Transmembrane Domain Composition Reflects Subcellular Localization of SNARE Proteins

**DOI:** 10.1111/tra.70059

**Published:** 2026-11-09

**Authors:** Christian Baumann, Carlos Pulido‐Quetglas, Dirk Fasshauer

**Affiliations:** ^1^ Department of Computational Biology University of Lausanne Lausanne Switzerland

**Keywords:** amino acid composition, endomembrane system, lipid‐protein interactions, membrane protein localization, molecular dynamics simulations, SNARE proteins, transmembrane domains

## Abstract

Transmembrane domains (TMDs) anchor proteins within membranes of distinct composition and biophysical properties, necessitating adaptation to each membrane's unique environment. SNARE proteins, the core mediators of eukaryotic membrane fusion, provide an ideal system to study such adaptations: they are ubiquitous across Eukaryota, with paralogs localized to different compartments that have evolved independently to match their membrane context. Here, we combine statistical analyses of approximately 14 000 SNARE TMDs with MD simulations of 28 human SNARE TMDs to uncover how TMDs have adapted to distinct membrane environments. We identify a clear compositional dichotomy that separates SNAREs of the early secretory pathway (ER and Golgi apparatus) from those of the late secretory and endosomal pathways: early TMDs are enriched in bulky Phe residues, whereas late TMDs are dominated by smaller Ile residues. The larger Phe side chains likely enhance hydrophobic interactions in loosely packed ER and Golgi membranes, while Ile likely prevents disruption of the tight lipid‐lipid packing in the plasma and endosomal membranes, potentially contributing to protein localization. TMD lengths derived from MD simulations differ from those inferred by sequence‐based approaches and show limited variation across compartments. Together, these results reveal how variations in membrane composition shaped SNARE TMDs across eukaryotic compartments.

## Graphical abstract

SNARE transmembrane domains (TMDs) match the distinct lipid environments of eukaryotic compartments. By analyzing ~14 000 SNARE TMD sequences across Eukaryota, we identify a sharp compositional split between ER/Golgi and PM/endosomal SNARE TMDs. Molecular dynamics simulations of 28 human SNARE TMDs show limited variation in TMD length across compartments.

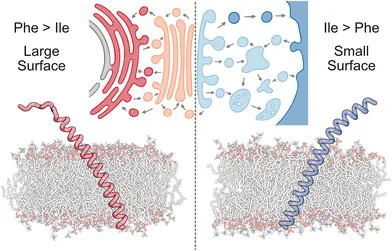

**Abbreviations**
AF2: AlphaFold 2
DMPC: 1,2‐dimyristoyl‐sn‐glycero‐3‐phosphocholine
DOPC: 1,2‐dioleoyl‐sn‐glycero‐3‐phosphocholine
ER: endoplasmic reticulum
ES: endosomal system
GA: Golgi apparatus
GES hydrophobicity scale: Goldman‐Engelman‐Steitz hydrophobicity scale
GET pathway: guided entry of tail‐anchored proteins pathway
HMM: Hidden Markov Model
LASA: lipid‐accessible surface area
LECA: last eukaryotic common ancestor
MD: molecular dynamics
O–O: oxygen‐to‐oxygen membrane thickness definition
PC1: principal component 1
PCA: principal component analysis
PCC: Pearson correlation coefficient
PLS‐DA: partial least squares discriminant analysis
PM: plasma membrane
P–P: phosphate‐to‐phosphate membrane thickness definition
SM proteins: Sec1/Munc18 proteins
SNARE: soluble *N*‐ethylmaleimide‐sensitive factor attachment protein receptor
SV: secretory vesicle
TGN: trans‐Golgi network
TMD: transmembrane domain

## 1 Introduction

Compartmentalization enables eukaryotic cells to perform complex biochemical processes in distinct local environments. Despite being physically separated, cellular compartments are not isolated; they continuously communicate and exchange materials through dynamic processes. One major mode of exchange occurs through vesicles that bud from one compartment and fuse with another. Membrane fusion is a tightly regulated process mediated by specific proteins, most notably the SNARE (soluble *N*‐ethylmaleimide‐sensitive factor attachment protein receptor) family. About 20 proteins of this family were already present in the last eukaryotic common ancestor (LECA), and have since diversified into numerous lineage‐specific variants, totaling 38 in humans (Figure 1A) [3, 4, 5, 6].

**FIGURE 1:**
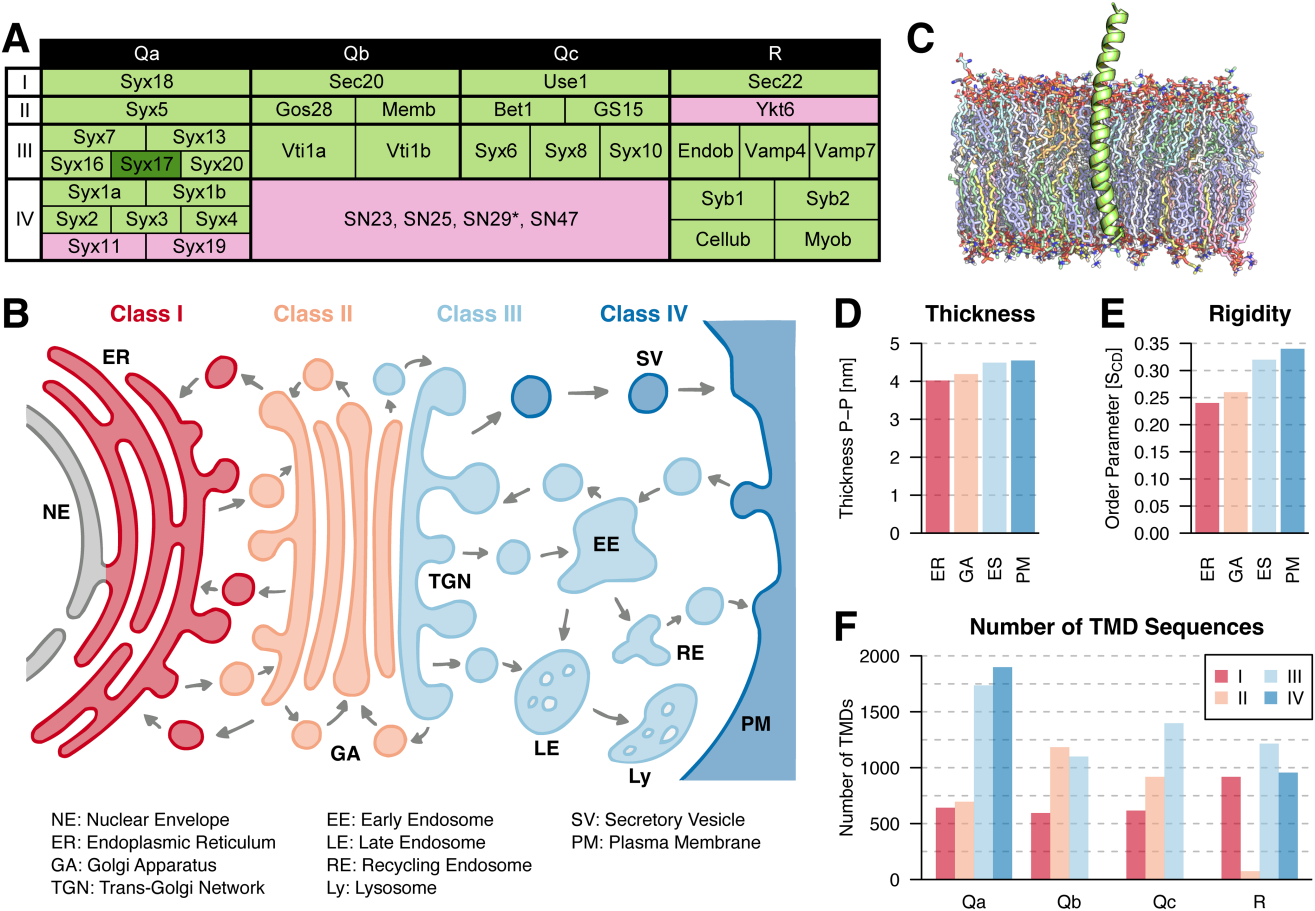
Overview of human SNAREs, membrane environments, and dataset composition. (A) SNAREs in humans. Proteins containing transmembrane domains (TMDs) are shown in green, whereas lipid‐anchored SNAREs are shown in pink. The TMD of Syx17 consists of two α‐helices rather than one. SN29 lacks any type of membrane anchor. (B) Schematic of cellular organization indicating the compartments in which SNAREs function: Class I at the endoplasmic reticulum (ER) (red), Class II at the Golgi apparatus (GA) (orange), Class III at the *trans*‐Golgi network (TGN) and endosomal system (ES) (light blue), and Class IV at the plasma membrane (PM) and secretory vesicles (SVs) (dark blue). (C) *C*‐terminal region of Syb2 embedded in a membrane, shown as an example of a SNARE TMD. The portion of the α‐helix buried within the lipid bilayer is referred to as the TMD. The lipid composition reflects that of the human erythrocyte PM [1]. (D) Membrane thickness in mammalian model membranes, measured as the distance between the phosphate atoms of opposing lipid leaflets. Based on data from Pogozheva et al. [2] (E) Lipid rigidity in mammalian model membranes, represented by the deuterium order parameter; smaller values indicate loosely packed, more flexible lipids. Based on data from Pogozheva et al. [2] (F) Number of SNARE TMD sequences analyzed in this study, categorized by type (Qa, Qb, Qc, R) and Class (I, II, III, IV).

SNAREs mediate membrane fusion by forming tetrameric complexes composed of SNAREs anchored in both participating membranes [7, 8]. The assembly of these complexes in a zipper‐like fashion draws the two membranes into close proximity, ultimately resulting in their fusion. Each tetramer consists of four distinct SNARE types (Qa, Qb, Qc, and R) that interact in a defined manner [9, 10]. The four types can be distinguished by characteristic sequence signatures. Their names stem from the conserved amino acids at the central layer of the SNARE complex: Q‐SNAREs contain a Gln, whereas R‐SNAREs carry an Arg residue. The four SNARE types can each be divided into four classes, which can be assigned to specific intracellular localizations (Figure 1B): Class I at the endoplasmic reticulum (ER), Class II at the *cis*‐ and medial Golgi apparatus (GA), Class III at the *trans*‐Golgi network (TGN) and endosomal system (ES), and Class IV at the plasma membrane (PM) and secretory vesicles (SVs) [3].

Most SNAREs are anchored in membranes via a *C*‐terminal transmembrane domain (TMD) (Figure 1C). Typically, the SNARE TMD consists of a single α‐helix that spans the lipid bilayer. Notable exceptions include Syx17, which contains a two‐helix TMD, and several SNAREs that are anchored via lipidation of cysteine residues [5, 11]. Like other tail‐anchored proteins, SNAREs are targeted post‐translationally to the ER membrane via the GET pathway and reach their destinations within the secretory or endocytic pathway through membrane trafficking [12, 13]. SNARE‐mediated membrane fusion is highly specific in that vesicles fuse only with defined target compartments, requiring the correct spatial localization of SNAREs within the cell. Two distinct mechanisms ensure correct SNARE sorting. First, specific sequence or structural motifs are recognized by, for example, sorting receptors or coat proteins [14, 15]. One example is the KDEL motif, which promotes ER retention and is found in a subset of Sec20 proteins [16, 17]. Second, the physicochemical properties of the TMD itself can drive lateral segregation within membranes, enabling sorting and, in some cases, being sufficient for correct SNARE localization [18, 19, 20, 21].

Eukaryotic endomembranes are complex mixtures composed of hundreds to thousands of distinct lipid species [22]. Lipid compositions vary between species, tissues, organelles, leaflets, and even membrane subdomains, with cells devoting substantial resources to maintain specific lipid distributions [22, 23]. Despite this diversity, membranes can be broadly categorized into two regimes with distinct characteristics and biological roles [24, 25]. The first regime comprises loosely packed, thin membranes enriched in unsaturated lipids, typical of early secretory compartments such as the ER and GA. The second regime includes tightly packed, thick membranes enriched in saturated lipids, sphingolipids, sterols, and negatively charged lipids, particularly in the cytosolic leaflet. This membrane type characterizes the PM, TGN and endosomal membranes, with the transition between the two regimes occurring from the medial Golgi stacks to the TGN. Membrane composition is not uniform across these two regimes but instead follows a continuous gradient along the secretory route from the ER to the PM: lipid monounsaturation decreases, while lipid saturation, sterol and sphingolipid content, transbilayer lipid asymmetry and negative surface charge on the cytoplasmic leaflet all increase [26, 27, 28]. These compositional gradients produce distinct physicochemical effects, including progressive increases in membrane thickness (Figure 1D) and lipid rigidity (Figure 1E) along the secretory pathway [2].

Membrane proteins evolved to match the physical and chemical properties of their native membranes [29, 30, 31, 32]. Research over the past decades has revealed several key principles, including that TMD length increases along the secretory pathway and contributes to protein sorting, that positively charged residues are predominantly found at the cytosolic side of the PM, and that TMD volumes are asymmetrically distributed between the two leaflets of the PM [29, 33, 34, 35, 36]. These studies did not focus specifically on SNAREs; however, Sharpe et al. showed that SNARE TMDs follow similar trends to those observed for other bitopic membrane proteins [29]. SNAREs provide an excellent model system for examining how membranes shape protein properties for two reasons: (1) SNARE classes do not form monophyletic groups, and thus highlight convergent adaptations to their native membranes [3, 37]. (2) All SNAREs perform the same conserved role in membrane fusion, minimizing the influence of functional divergence on potential differences in their TMDs. Moreover, we have established an extensive collection of classified SNARE sequences across Eukaryota, which allows for robust comparative analyses of their TMD sequences [3, 38, 39].

In this study, we analyze SNARE TMDs in terms of their amino acid composition, TMD length and lipid‐accessible surface area (LASA). Our results reveal that TMD amino acid composition and LASA reflect the two membrane regimes: ER and GA SNAREs are enriched in Phe and have a large LASA, whereas ES and PM SNAREs show higher levels of Ile and a small LASA, which likely reflects whether strong or weak protein‐lipid interactions are advantageous in their native membrane environment. TMD lengths derived from molecular dynamics (MD) simulations are surprisingly similar across classes, and short TMDs are not restricted to the early secretory pathway.

## 2 Results

### 2.1 Amino Acid Composition

SNAREs mediate membrane fusion between specific vesicle‐compartment pairs and therefore must be localized at the correct membranes within the cell. Since cellular membranes differ in their biophysical properties, we wondered whether these differences are reflected in the amino acid composition of SNARE TMDs. To address this question, we analyzed the amino acid composition of 13 941 SNARE TMD sequences from our extensive in‐house SNARE collection with respect to their site of action within the cell (Figure 1F). This analysis reveals pronounced class‐specific differences: Classes I and II, that is, SNAREs of the ER and GA, contain a higher proportion of Phe, whereas Classes III and IV, that is, SNAREs of the TGN, ES, SVs and PM, are enriched in Ile (Figure 2A), although considerable variation in individual amino acid frequency exists among TMDs within each class, resulting in substantial overlap between classes. Our results closely align with an earlier study by Munro, who analyzed the amino acid composition of GA and PM TMDs using a much smaller dataset [40]. Particularly, he also observed an enrichment of Phe in GA TMDs and of Ile in PM TMDs.

**FIGURE 2:**
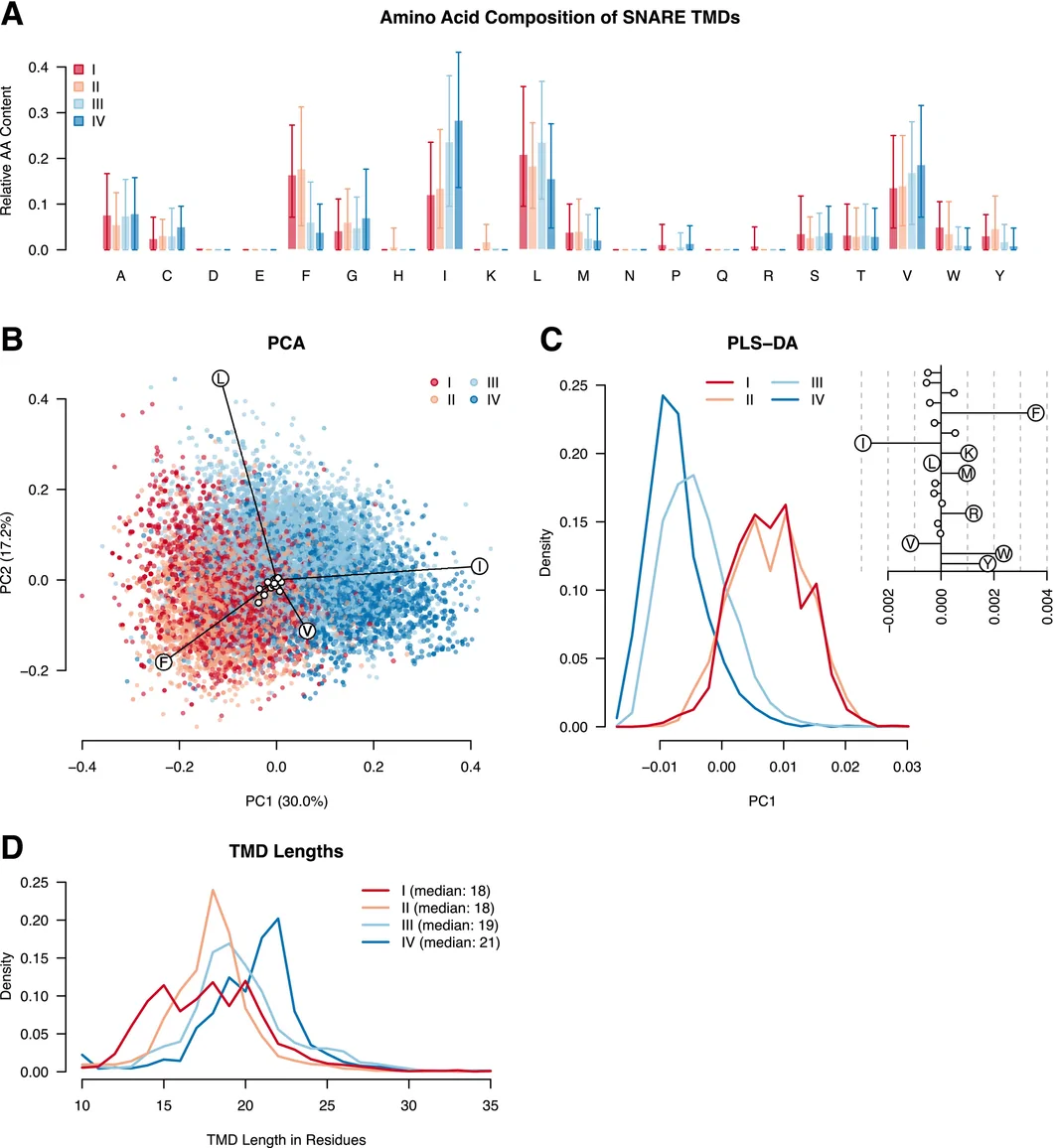
Amino acid composition and length of SNARE TMDs. (A) Mean amino acid composition of SNARE TMDs by class. TMDs were identified using the Goldman‐Engelman‐Steitz (GES) hydrophobicity scale with a rolling mean over 15 residues. Error bars represent the range containing 80% of all TMDs. (B) Unscaled principal component analysis (PCA) of SNARE TMD amino acid composition with corresponding relative loadings. Each data point represents a single TMD sequence. A scaled version of this PCA can be found in Figure S1A,B. (C) Histogram of partial least squares‐discriminant analysis (PLS‐DA) principal component 1 (PC1) scores depicting the separation between Classes I/II and Classes III/IV. Feature weights contributing to the discrimination are shown in the upper‐right panel. (D) Histogram of sequence‐derived TMD lengths of SNAREs calculated using the GES hydrophobicity scale.

Principal component analysis (PCA) reveals a clear compositional dichotomy between Classes I/II and III/IV, demonstrating that the split is evident not only in average composition but also at the level of individual TMDs (Figure 2B). As seen with the averages, the split into the two groups is driven by the differences in Phe and Ile content. Besides these large compositional differences, Classes I/II and III/IV also differ in less frequently occurring amino acids. Using a scaled partial least squares‐discriminant analysis (PLS‐DA), we separated Classes I and II from Classes III and IV (Figure 2C). In this analysis, Phe and Ile remain the dominant contributors to the separation, with smaller contributions from additional amino acids being present as well. Aromatic residues (Trp and Tyr) are more abundant in Classes I and II, as are Met, Lys, and Arg to a lesser extent. However, positively charged residues are overall rare in TMDs and are only present in a subset of proteins. Specifically, Lys occurs in 33.0% of Class II and 5.5% of Class I TMDs, whereas Arg is found in 16.9% of Class I and 1.5% of Class II TMDs. In Classes III and IV, it is only Val that additionally occurs notably more frequently than in Classes I and II. The average Val content continually increases along the secretory pathway.

Although the two dominant compositional groups are evident, the distributions partially overlap. We therefore examined the TMD amino acid composition of all SNAREs individually to see if there were any that could not be easily assigned to either group. Two SNAREs in particular, Gos28 of the Qb II and Nyv1 of the R III subtype, display intermediate properties. The intermediate position stems from a low Phe content in the case of Gos28 and a low Ile content in Nyv1 proteins.

In addition to the clear split between Classes I/II and III/IV, the individual SNARE classes can also be separated with PLS‐DA, even though a larger overlap remains (Figure S1C,D). Nearly all amino acids contribute to the partial differentiation between Classes I and II, including that Class II has more Lys and Class I more Arg. The separation between Classes III and IV is similarly complex, but it is dominated by a higher Leu content in Class III TMDs. This higher Leu content in Class III is also apparent in the mean amino acid composition (Figure 2A) and to some degree in the PCA (Figure 2B). This indicates that although Leu is common in all TMDs and does not contribute to the dichotomy, it helps distinguish Classes III and IV.

SNARE TMDs might be influenced by potential variations in membrane compositions between phylogenetic groups. To investigate these potential differences, PCA and PLS‐DA were performed separately for metazoan, fungal, archaeplastid, and SAR SNARE TMDs (Figures S2–S4). Overall, the main compositional dichotomy between Classes I/II and III/IV, and the differences among individual classes persist across all phylogenetic groups, but their magnitudes vary. Notably, fungi show a clear separation between Classes III and IV, which is evident from PCA alone (Figure S3E). Similar to the complete dataset, fungi Class III TMDs contain more Leu than Class IV TMDs, which in turn contain somewhat more Val and Ile. In metazoan SNAREs, the Class I versus II split is more distinct than in the other taxonomic groups and shows again the complex contribution from many amino acids (Figure S3C). The characteristic Ile‐Phe dichotomy is conserved across all groups, while the specific amino acids contributing to finer class‐level separations differ to some extent among lineages. This suggests that SNARE TMDs may have undergone lineage‐dependent adaptations.

In contrast to the dichotomy between early and late secretory pathway, the four basic SNARE types (Qa, Qb, Qc, and R) cannot be distinguished by their TMD composition. Both PCA and PLS‐DA show that the four SNARE types overlap extensively in their TMD amino acid composition (Figure S5). In contrast to the TMDs, the composition of the adjacent SNARE motifs is sufficient to distinguish the four SNARE types [10]. This distinction is expected, as each of the four SNARE types forms a monophyletic group. Differences between the four SNARE types become apparent only when SNAREs are analyzed within a single class (Figures S6 and S7). These type‐specific differences are most pronounced in Classes I and II. Taken together, these findings suggest that the amino acid compositions of SNARE TMDs represent independent adaptations within each SNARE type to the distinct biophysical properties of membranes across cellular compartments.

### 2.2 TMD Length

Proteins of the late secretory pathway are thought to have longer TMDs than those of the early secretory pathway, consistent with the increasing thickness of these membranes (Figure 1D). This finding is largely based on sequence predictions. TMDs are commonly identified from amino acid sequences using hydrophobicity‐based scoring methods that estimate whether residues are more likely to reside within the membrane or in aqueous environments [41]. A wide range of different hydrophobicity scales and online prediction tools are available for this purpose. In their study, Sharpe et al. used the Goldman‐Engelman‐Steitz (GES) hydrophobicity scale to show that TMD lengths of bitopic membrane proteins differ between early and late secretory pathways [29, 42]. Using a similar GES‐based approach, we obtained comparable results for our SNARE dataset, with TMD lengths increasing along the secretory pathway (Figure 2D).

Sequence‐based methods delineate hydrophobic stretches but do not necessarily capture the actual membrane‐embedded boundaries of TMDs. We therefore asked whether the observed increase in TMD length persists when TMDs are defined by their membrane positioning in all‐atom MD simulations. Given the computational cost of all‐atom simulations for the complete SNARE repertoire, we limited our analysis to human SNARE TMDs. In addition, due to the complexity of biological membranes, simulations were performed in simplified model bilayers that approximate physiological conditions by incorporating only the most essential components.

Although this model approach does not capture the full diversity of membrane environments, it preserves the key interactions required for comparative analysis of TMD length. However, it does not account for factors such as membrane curvature or specific TMD‐lipid interactions, which may influence TMD behavior. Likewise, it does not address whether individual TMDs preferentially partition into membranes of defined thickness. Nevertheless, this model‐based framework provides a structurally informed definition of TMD length, thereby complementing sequence‐based approaches.

Simulations of all human SNARE TMDs were conducted in both DMPC and DOPC membranes, which differ in acyl‐chain length and saturation and therefore provide bilayers of distinct thickness (DMPC: ~36.0 Å, DOPC: ~38.6 Å considering the phosphate planes) and fluidity. Both bilayers are either thinner than or comparable in thickness to the mammalian ER membrane (40.2 Å [2], 37 Å [43]), which is thought to be the thinnest membrane in the cell. This ensures that even the shortest SNARE TMDs should be accommodated without artificial elongation due to hydrophobic mismatch. Conversely, longer TMDs are expected to fully bury their hydrophobic residues, for example through increased tilting. To ensure that TMD length differences can be resolved by our MD setup, we generated elongated and truncated variants of two SNARE TMDs and found that the resulting changes in TMD length were readily detected (Figures S8 and S9).

Because membrane‐spanning boundaries are not uniquely defined, we quantified TMD lengths using two complementary measures: the number of Cα atoms located between the acyl‐oxygen (O–O) or phosphate (P–P) planes of opposing leaflets (Figure 3A). For each SNARE, TMD lengths fluctuate substantially over time (Figure 3B,C). Nevertheless, replicate simulations and lipid compositions yield largely consistent results, with Pearson correlation coefficients (PCC) between DOPC and DMPC of 0.64 (O–O) and 0.90 (P–P), indicating minimal dependence on the lipid model, particularly for the P–P definition (Figure S10A,B).

**FIGURE 3:**
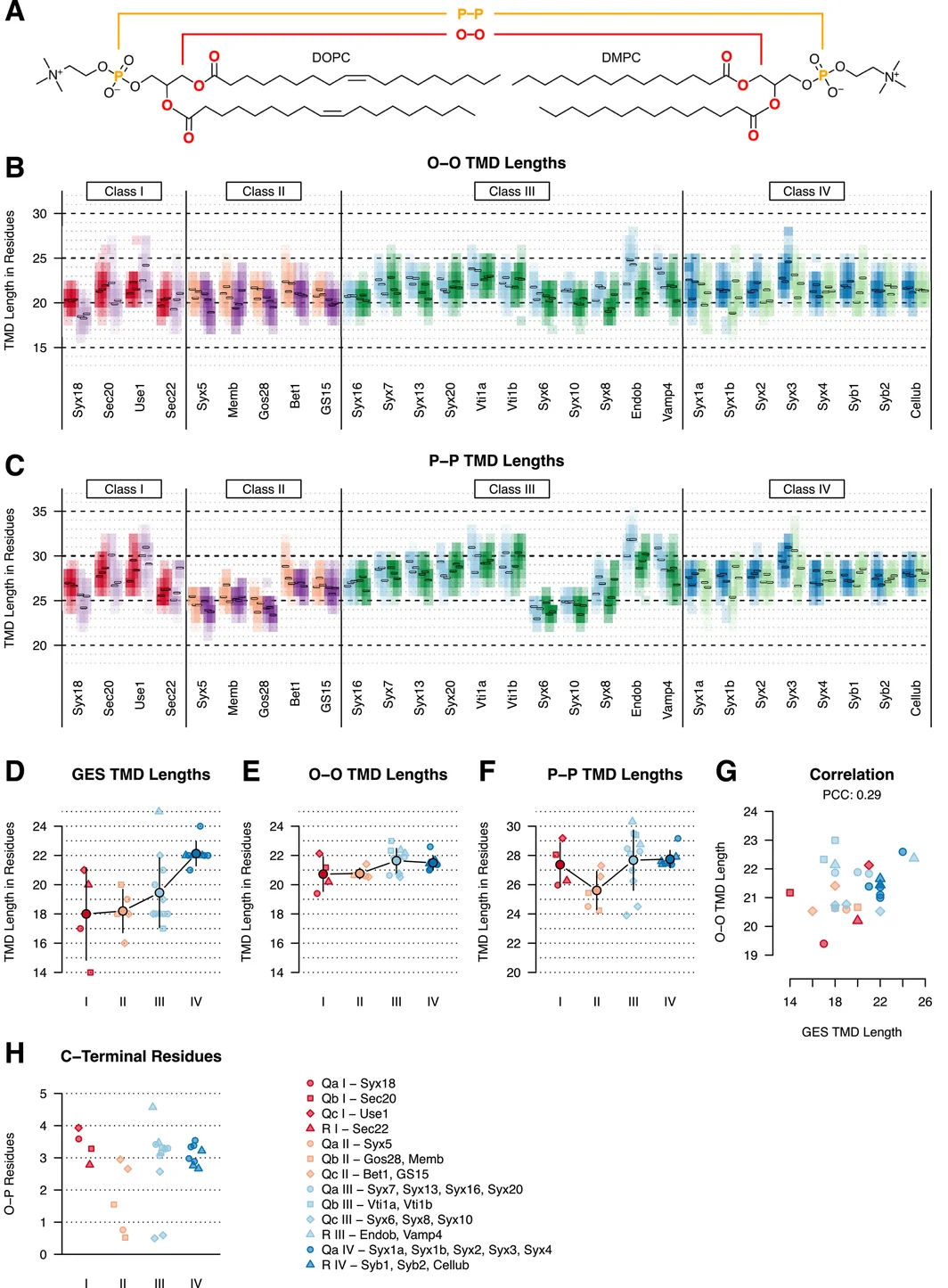
TMD lengths of human SNAREs determined from molecular dynamics (MD) simulations and sequence data. (A) Lipid molecules used in the MD simulations. Atoms used to define the membrane boundaries are highlighted in orange (phosphorus atoms for P–P definition) and in red (acyl acid oxygens for O–O definition). (B) Frequency heatmap of TMD lengths from MD simulations of human SNAREs. Lengths were determined by the Cα atoms located within the O−O layers considering the 10 lipids closest to the TMD in each leaflet. MD simulations were performed in triplicate for each SNARE TMD in DOPC and DMPC bilayers. For each TMD, results obtained with DOPC are shown on the left and those with DMPC on the right using complementary colors. Color intensity indicates how frequently a given TMD length was observed. The average TMD length for each trajectory is shown as a short bar. (C) Frequency heatmap of human SNARE TMD lengths from MD simulations, based on the number of Cα atoms located within the P–P layers considering the 10 lipids closest to the TMD in each leaflet. Other details are the same as in subfigure B. (D) TMD lengths of human SNAREs calculated from sequence data using the GES scale. Values for individual SNAREs are plotted in the background, while class averages are shown in the foreground; bars indicate standard deviations. (E) Average TMD lengths from MD simulations in both membrane types using the O–O definition. Individual protein averages are plotted in the background, class averages in the foreground; bars indicate standard deviations. (F) Average TMD lengths from MD simulations in both membrane types using the P–P definition. Individual protein averages are plotted in the background, class averages in the foreground; bars indicate standard deviations. (G) Correlation between TMD lengths obtained from MD simulations in both membrane types (O–O definition) and those derived from sequence data using the GES hydrophobicity scale. (H) Number of residues located between the oxygen and phosphate layers in the outer (luminal or exoplasmic) leaflet considering both membrane types. The difference between the O–O and P–P thickness is approximately 8.5 Å, corresponding to about 5.5 residues in an α‐helix (1.5 Å per residue). Roughly half of this distance (∼3 residues) is expected to lie between the O and P layers. Smaller numbers indicate that the *C*‐terminus does not reach the phosphate layer.

Using the O–O criterion, TMD lengths fall mostly between 20 and 25 residues, with little variation among SNAREs (Figure 3B). Greater heterogeneity is observed with the P–P definition, where most SNARE TMDs span 25 to 30 residues, but five proteins (Syx5, Memb, Gos28, Syx6, and Syx10) exhibit noticeably shorter apparent lengths (Figure 3C). This reduction arises because their *C*‐termini fail to fully traverse the distance between the oxygen and phosphate planes at the outer (luminal) leaflet, resulting in fewer residues between these planes compared with other SNAREs (Figures 3H, S10C,D).

In contrast to the sequence‐based GES analysis (Figure 3D), the trend of increasing TMD length along the secretory pathway is markedly attenuated in the MD simulations. Average O–O‐based lengths increase only marginally across Classes I–IV (Figure 3E), remaining well within the intrinsic fluctuations observed during the simulations and substantially smaller than the differences inferred from GES. A similar pattern is observed for the P–P definition, where the shorter average length of Class II TMDs is driven by three proteins with short *C*‐termini (Figure 3F). Excluding the five shortest TMDs aligns Class II more closely with Class I, while Class III TMDs become slightly longer than those of Class IV (27.4, 26.9, 28.4 and 27.7 residues for Classes I–IV). A graphical overview of the various TMD segments identified with each method for human SNAREs is provided in Figure S11.

The discrepancy between GES‐ and MD‐derived TMD lengths is further evident in the weak correlation between them: The PCC is 0.29 between GES and O–O TMD lengths (Figure 3G) and 0.28 between GES and P–P TMD lengths (Figure S10E). This creates a potential issue for the statistics on TMD amino acid composition we presented in the previous section, where we aimed to analyze the composition of the membrane‐embedded portion of the protein. If TMD boundaries are not accurately captured with GES, then the amino acid statistics could be considerably different with the MD‐defined boundaries. To evaluate this effect, amino acid compositions of human SNARE TMDs were recalculated. Amino acid contents remain similar overall between GES and O–O TMDs, whereas larger differences occur with P–P TMDs (Figure S12A). This is consistent with the smaller length differences between GES and O–O TMDs and the considerably longer spans of the P–P TMDs. The longer P–P spans include regions flanking the hydrophobic core, leading to a general decrease in hydrophobic amino acid content and an increase in positively charged residues.

Similarly, PLS‐DA scores were recalculated for the human SNARE TMDs to assess the impact of the different TMD definitions (Figure S12B–D). Although the separation between Classes I/II and III/IV becomes less pronounced, especially for P–P TMDs, the scores remain strongly correlated across methods (PCC = 0.82–0.89). Interestingly, Gos28 becomes even more similar to Class III/IV TMDs with the O–O criterion than was the case with GES. However, because the PLS‐DA model was trained on GES‐defined TMDs, recalibrating it with MD‐based TMDs could improve the distinction between Classes I/II and III/IV again.

TMDs may also differ between compartments in properties other than length. We therefore analyzed the tilt and curvature of human SNARE TMDs. Tilt angles fluctuate substantially but show no systematic differences between compartments and are generally larger in the thinner DMPC bilayer than in DOPC (Figure S13). MD‐derived TMD length and tilt are moderately correlated, with longer TMDs displaying larger tilt angles. Similarly, TMD curvature fluctuates over the course of the simulations without compartment‐specific trends and shows a similar moderate correlation with TMD length, such that longer TMDs tend to be more curved (Figure S14). A notable SNARE is Vti1b, the TMD of which displays pronounced curvature in DOPC due to a charged Glu residue within the TMD (Figure S15).

Because both DOPC and DMPC represent relatively thin membranes, we additionally performed simulations of a subset of TMDs (without replicates) in a thicker bilayer composed of 60% DOPC and 40% cholesterol (Figure S16). Cholesterol incorporation increases DOPC bilayer thickness from ~38.6 to ~43.7 Å (based on P–P distances), resulting in a general elongation of TMDs. However, O–O TMD lengths remain comparable across classes, and the Syx5 TMD remains the shortest P–P TMD within the tested subset. Interestingly, P–P Class IV TMDs are shorter than Class I TMDs in this subset.

### 2.3 Lipid‐Accessible Surface Area

TMDs of ER and GA SNAREs differ from those in the TGN, ES, SV, and PM in their Phe and Ile content. Both amino acids are among the most hydrophobic residues, but they differ in side‐chain architecture: Phe carries a bulkier side chain than Ile, providing a larger surface for interactions with surrounding lipids (Figure 4A). However, side‐chain geometry alone can play an important role: Despite differing only by the position of a single methyl group, Leu generates a larger interaction surface than Ile because the γ‐branch in Leu positions its methyl group farther from the protein backbone (Figure 4A) [44].

**FIGURE 4:**
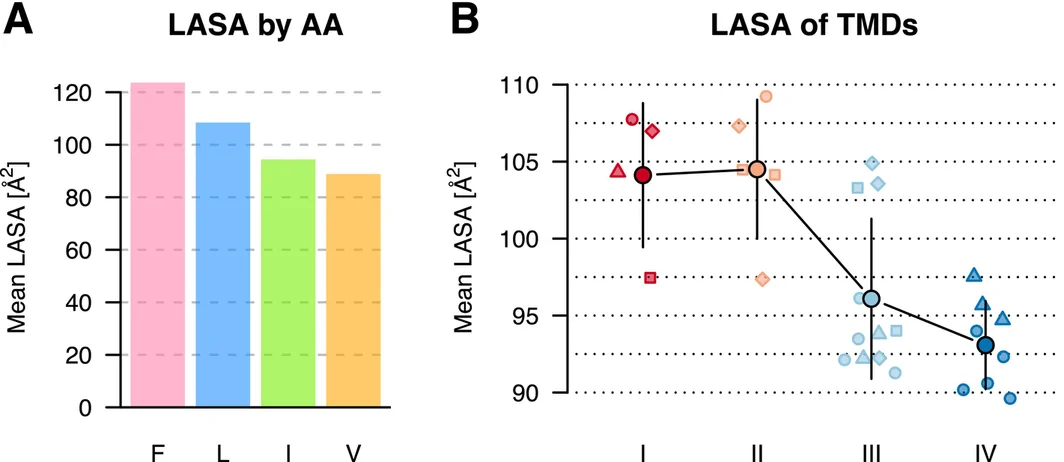
Lipid‐accessible surface area (LASA) of human SNAREs. (A) Mean LASA of Phe, Ile, Leu, and Val within a model TMD. Twenty residues in the TMD of Syb2 (M29–F48, Alignment S1) were mutated to each of the four amino acids. The bar plot shows the average LASA per residue within the mutated span. (B) Average LASA per residue of human SNARE TMDs. Average values of individual SNAREs are shown in the background, class averages are displayed in the foreground, and error bars indicate the standard deviation across individual SNAREs.

To determine whether the compositional differences translate into altered lipid interaction surfaces, we calculated the LASA for human SNARE TMDs (Figure 4B). Indeed, the average LASA per residue calculated from MD simulations using the O–O definition shows a pronounced difference between Classes I/II and III/IV, with Classes I and II exhibiting the larger lipid‐interacting surfaces. Some exceptions exist: Sec20 (Class I) and GS15 (Class II) show markedly smaller LASAs, whereas Vti1b, Syx6, and Syx10 (all Class III) display larger values, all of which resemble more closely the values of the opposite group. Notably, Syx6 and Syx10 are also among the TMDs with shorter MD‐derived lengths.

In the human SNARE TMD set, and considering the four major hydrophobic amino acids, Phe and Ile contents correlate most strongly with LASA, indicating that these residues are the primary drivers controlling lipid accessibility (Figure S17A–D). Accordingly, the PLS‐DA scores for amino acid composition closely mirror the LASA values (Figure S17E). Some of the largest deviations occur in the five TMDs with unexpectedly large or small LASA and, notably, in Gos28. Although its PLS‐DA score places Gos28 closer to Class III/IV SNARE TMDs in terms of composition, its LASA remains large and comparable to that of other Class I/II members. This elevated LASA is driven at least in part by its high Leu content, a pattern also observed in Vti1b, Syx6, and Syx10, all of which contain relatively few Phe residues and appear to achieve a large LASA through increased Leu levels.

### 2.4 *C*‐Terminal Ends of TMDs

In most human SNAREs, the TMD extends no more than a single residue beyond the luminal phosphate plane of the membrane (Figure S10D). In some cases, the TMD is shorter and ends before reaching the phosphate plane, as mentioned above, whereas in a few proteins it extends beyond this boundary, such as in Sec20, GS15, Use1, and Vti1a. Among the latter, Sec20 exhibits the largest number of residues extending beyond the membrane with an average of four. The Sec20 *C*‐terminus found in the human protein is highly conserved within animals of the deuterostome lineage but varies across eukaryotic phyla. In many plants and fungi, the Sec20 *C*‐terminus is substantially longer, extending beyond 100 residues, and contains a KDEL signal or related motif such as RDEL or HDEL for ER retention [16].

The two longest *C*‐termini in human SNAREs are found in Vamp7 and Myob, which were not included in the analysis because their extended *C*‐termini partially insert into the membrane during the MD simulations, thereby interfering with the measurements. The *C*‐terminus of human Myob contains roughly 22 residues and is most likely intrinsically disordered based on its sequence. Similar luminal *C*‐terminal extensions are also found in several non‐human R IV proteins. Vamp7 possesses a highly conserved *C*‐terminal region that AlphaFold 2 (AF2) predicts will fold back on itself and form a disulfide bond (Figure 5H). This curled *C*‐terminus is not only present in the human ortholog but is highly conserved across all major eukaryotic lineages, suggesting that it represents the ancestral state already present in the LECA (Figure S18).

**FIGURE 5:**
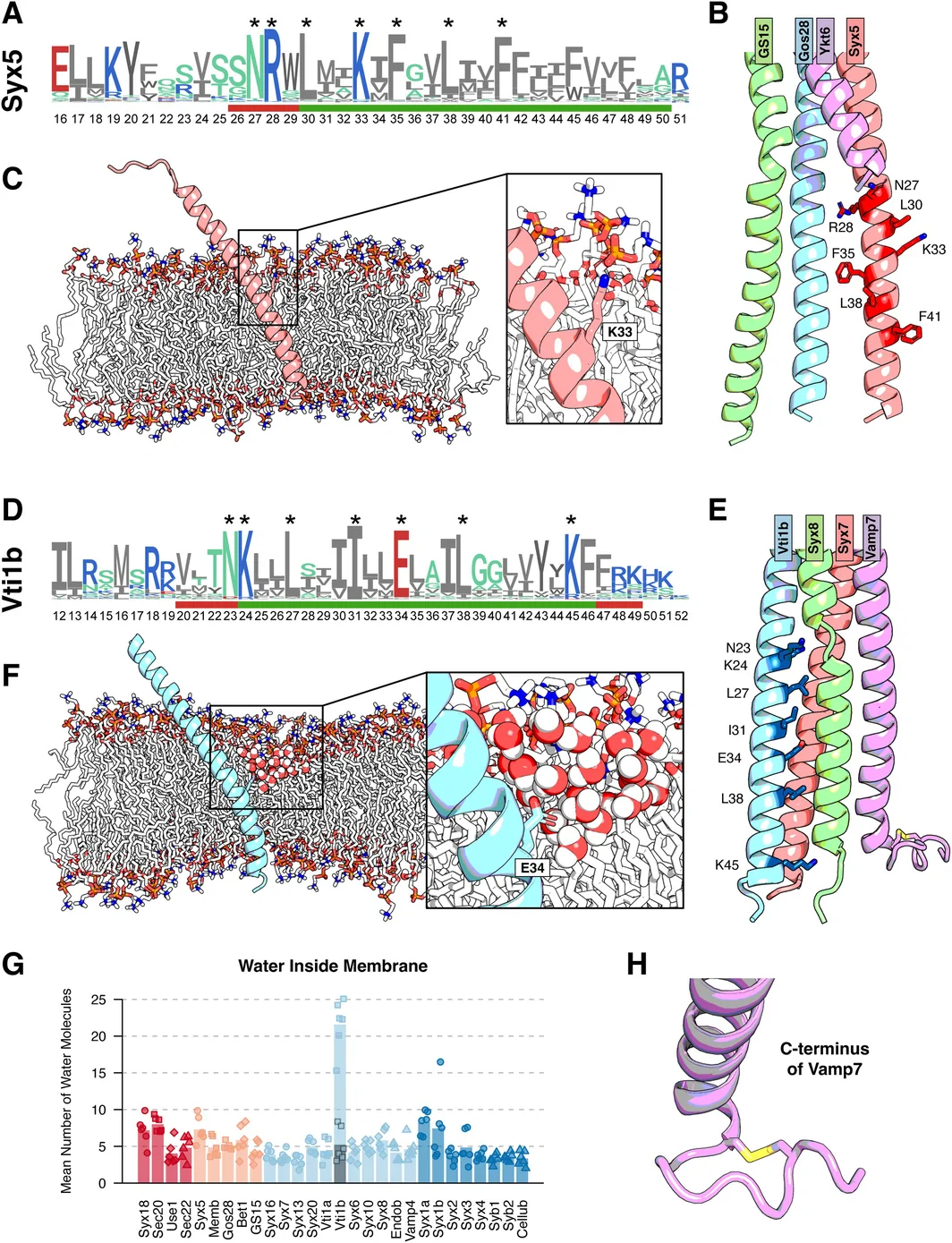
Potentially charged residues within Syx5 and Vti1b TMDs. (A) Sequence logo of Syx5 based on 652 sequences. Stars mark the seven most conserved residues in the TMD. Red and green bars indicate the TMD span based on the P–P and O–O definitions, respectively. The *C*‐terminal residue is not present in human Syx5. Amino acids are color‐coded according to their chemical properties. Sequence numbering corresponds to the models used in MD simulations, which were based on the last 50 residues of the human SNAREs. (B) AF2 model of the tetramer formed by human Syx5, Gos28, GS15, and Ykt6. Side chains of the seven most conserved TMD residues in Syx5 are highlighted. Ykt6 lacks a TMD and is membrane‐anchored via palmitoylation. (C) MD snapshot of Syx5 embedded in a DOPC bilayer. The charged residue K33 is positioned within the membrane, but its side chain extends toward lipid phosphates and headgroups. (D) Sequence logo of Vti1b based on 154 sequences. Stars mark the seven most conserved residues in the TMD. Red and green bars indicate the TMD span based on the P–P and O–O definitions, respectively. Amino acids are color‐coded according to their chemical properties. Sequence numbering corresponds to the models used in MD simulations, which were based on the last 50 residues of the human SNAREs. (E) AF2 model of the tetramer formed by human Syx7, Vti1b, Syx8, and Vamp7. The seven most conserved residues in Vti1b are highlighted. The AF2 model predicts a potential disulfide bond at the *C*‐terminus of Vamp7. (F) MD snapshot of Vti1b in a DOPC bilayer. The buried charged residue E34 induces the formation of a water pocket within the membrane. (G) Number of water molecules within the membrane based on the O–O definition. Only water molecules within 10 Å (in the x‐y plane) around the TMD center were considered. Bars represent protein averages; data points show averages from individual trajectories. The gray Vti1b bar indicates the result for the protonated (and therefore neutral) E34. (H) AF2 model of the *C*‐terminal end of human Vamp7. The predicted disulfide bond is highlighted in yellow.

### 2.5 Conserved Charges in TMDs

Our sequence analysis shows that potentially charged residues (Arg, Lys, His, Asp, Glu) are rare within SNARE TMDs but are slightly enriched in ER and GA SNARE TMDs, where they are present at very low levels (Figure 2A). Using the GES‐based boundaries, only four of the 28 investigated human SNAREs contain a single potentially charged residue within their TMD: Sec22 (Arg), Syx5 (Lys), Gos28 (His), and Vti1b (Glu). While the His in Gos28 is located at the *C*‐terminus, the charged residues in the other SNAREs lie within the hydrophobic core of the TMDs, with the Glu in Vti1b positioned at its very center. Whereas the potentially charged residues are highly conserved in Syx5 and Vti1b, the situation is a bit more complex in the other two proteins. The *C*‐terminus of Gos28 is most often formed by a potentially charged residue, which is typically Arg or Lys outside the animal lineage of Deuterostomia, within which it is predominantly His. Similarly, most Sec22 proteins contain either a Lys or Arg residue toward the *C*‐terminal end of the TMD. However, the exact position of this potentially charged residue does not appear to be conserved.

The membrane interior is highly hydrophobic, disfavoring polar or charged groups unless they can form compensating stabilizing interactions. Thus, the presence of even a few potentially charged residues within SNARE TMDs is surprising and raises the question of how they are accommodated within the hydrophobic environment of the membrane. Using snapshots from the MD simulations of the monomers and AF2 predictions of the tetramers, we took a closer look at the potentially charged residues in Syx5 and Vti1b.

Syx5 contains a Lys within its TMD that is highly conserved across Eukaryota (Figure 5A). In addition to this Lys, several other residues are strongly conserved, but they do not align on the same face of the helix as can be seen in a model of the Golgi SNARE complex, indicating that they are unlikely to form a shared interaction surface (Figure 5B). The long aliphatic chain of Lys allows its Cα to remain buried in the membrane while the terminal amino group reaches and interacts with the polar headgroup region of the lipids, a conformation commonly referred to as “snorkeling” (Figure 5C) [45]. It is therefore plausible that this Lys remains positively charged in the membrane and that it has a role in restricting the orientations that the Syx5 TMD can adopt. This interpretation is further supported by the observation that substitution of Lys with Leu promotes deeper insertion of the TMD into the membrane, resulting in partial membrane insertion of several residues flanking the TMD at the *N*‐terminal side (Figure S19). Despite the short TMD of Syx5, the snorkeling of the Lys residue is likely not a consequence of hydrophobic mismatch in our simulation setup, as it remains membrane‐embedded even when the TMD is extended by seven residues (Figure S9). Interestingly, a few exceptions exist in which Lys is substituted by Gln, such as in *Plasmodium* and *Caenorhabditis* (including *C. elegans* ) Syx5, which might fulfill a similar functional role.

The transition to multicellularity at the base of Metazoa was accompanied by an expansion of the set of endosomal SNAREs through the emergence of new paralogs [4]. Vti1b is such a metazoan SNARE and contains a highly conserved Glu within its TMD (Figure 5D). Unlike Syx5, the seven most conserved residues in Vti1b align on the same face of the helix, likely forming the TMD‐TMD interaction surface in the tetrameric complex (Figure 5E). Consequently, the potentially charged Glu residue is buried within the tetrameric core, where it may interact with backbone amides, as no complementary charged or polar side chains are expected nearby.

While the tetrameric arrangement poses no issue for a charged Glu, the situation is different in the monomeric Vti1b (Figure 5F). In the absence of a stabilizing partner, the charged Glu side chain promotes the formation of a water pocket within the membrane interior and induces bending of the TMD (Figure S15). No other SNARE TMD produced similar water‐filled cavities in the MD simulations (Figure 5G). However, the low dielectric constant of the membrane interior can substantially shift the pKa values of ionizable residues, potentially allowing their side‐chain charges to be neutralized even at physiological pH [46]. When Glu is protonated, and thus neutral, no water pocket forms in MD simulations. The protonation does not affect MD‐derived TMD length (Figure S20), suggesting that other residues, particularly positively charged amino acids near both TMD flanks, determine the effective span. The strong membrane deformation by charged Glu suggests that the Glu residue may be protonated and thus neutral in vivo or that an interaction partner provides a compensating charge in the native membrane.

TMD boundaries are commonly assigned based on the position of charged residues in sequence‐based prediction, even though such residues can be partially or fully buried within the membrane, as illustrated for Syx5 and Vti1b. Based on sequence alone, only 4 of the 28 analyzed human SNAREs contain potentially charged residues within their TMD. This assessment changes markedly when MD‐derived boundaries are considered. Using the O–O definition, the TMD is frequently shifted toward the *N*‐terminus relative to the GES‐defined TMD, resulting in the inclusion of additional *N*‐terminal residues and the exclusion of *C*‐terminal residues (Figure S11). As a result, 80% of the analyzed human SNAREs contain at least one potentially charged residue within the membrane‐embedded segment using the O–O criterion. Syx5 is therefore not an outlier with its Lys but instead displays a common feature among SNARE TMDs. The potentially charged residues present in O–O TMDs occur close to the cytosolic boundary and are positioned one to three residues closer to the membrane‐water interface than in Syx5. Even more charged residues are included with the P–P definition because this boundary encompasses additional flanking residues. Under this definition, all TMDs contain at least two potentially charged residues.

## 3 Discussion

The starting point of this study was the question of whether physicochemical differences between intracellular membranes are reflected in the amino acid composition of SNARE TMDs. Our analysis reveals that SNARE TMDs segregate into two broad compositional groups that correlate with their subcellular localization. SNAREs functioning at the ER and Golgi apparatus are enriched in Phe, whereas those acting at the *trans*‐Golgi network, the endosomal compartments, secretory vesicles and the plasma membrane contain higher levels of Ile, with smaller contributions from additional residues. Importantly, this dichotomy is evident not only in average compositions but also at the level of individual TMDs. A difference in Phe and Ile composition between GA and PM TMDs was reported previously by Sean Munro based on a limited dataset of various proteins [40]. Our study extends this principle to a larger TMD collection and shows that such compositional biases are a robust feature within the SNARE family across the eukaryotic endomembrane system. In addition to the work by Sean Munro, several other studies have reported related observations on TMD amino acid composition and cellular localization, including analyses that specifically addressed SNAREs [19, 34, 47, 48, 49]. Collectively, these observations are consistent with the patterns identified in our dataset.

We next asked how these compositional differences relate to subcellular localization: do they represent adaptations to the physicochemical properties of the native membranes, or do they actively contribute to protein sorting? Proper SNARE localization is essential, as only correctly distributed SNAREs can assemble into productive fusion complexes. Because SNAREs reside in the fused membrane after they catalyze membrane fusion, they must be actively trafficked back to their original compartments to enable subsequent fusion events. Thus, SNARE distribution must be continuously maintained by retrieval and re‐sorting mechanisms, making their localization not static but the result of ongoing sorting. This behavior contrasts with that of many other membrane proteins, which are typically confined to a single compartment and its corresponding membrane. The dynamic localization of SNAREs implies that their TMDs are exposed to a range of membrane environments, limiting their utility as precise reporters of individual intracellular membranes. Nevertheless, individual SNAREs remain functionally restricted to specific regions within the cell, for which membranes are expected to share similar physicochemical characteristics.

SNARE distribution is governed by multiple determinants. These include protein–protein interactions with SM proteins, tethering factors, sorting receptors and coat complexes through defined sorting signals in either soluble domains or the TMD [14, 15]. For example, the *C*‐terminal KDEL motif of Sec20 mediates retrieval to the ER, and a dileucine‐based motif in Vamp4 likely contributes to its trafficking itinerary [16, 50]. In addition, a folded epitope in Sec22, present only in the unassembled SNARE, is recognized by a COPII subcomplex to mediate ER export [51].

Beyond these canonical sorting determinants, the physicochemical properties of the TMD itself may contribute to localization [19, 52]. The compositional dichotomy we observe in SNARE TMDs is consistent with such a role. However, these differences could also simply reflect energetically favorable protein‐lipid interactions within distinct membrane environments [44, 47]. Along the secretory pathway, membrane rigidity increases, with ER membranes being the most loosely packed and deformable, whereas the PM is the most rigid and tightly packed (Figure 1E). Loosely packed membranes contain more packing defects, favoring extensive protein‐lipid interactions. In contrast, tight lipid packing in membranes such as the PM disfavors such interactions, as they would disrupt lipid‐lipid contacts. Consequently, TMDs in rigid membranes may minimize their hydrophobic lipid contact area through a reduction in LASA, whereas TMDs in more deformable membranes may benefit from maximizing it.

Importantly, these membrane adaptation and sorting through TMD properties are potentially linked phenomena [40, 47]. At the TGN, lipid microdomains (also referred to as lipid rafts) have been proposed to contribute to the sorting of a subset of PM‐directed proteins [53, 54, 55, 56]. TMDs with low LASAs preferentially partition into these lipid microdomains, whereas specific residues such as Phe and Leu can reduce raft affinity and thereby influence trafficking routes [49, 57]. Based on the calculated LASA values for human SNAREs, raft affinity appears to be a common feature of Class III (TGN, ES) and Class IV (SVs, PM) SNAREs, suggesting that TMD‐dependent partitioning could support their forward trafficking. Although this tendency is not evident from average LASA values alone, the higher Leu content in Class III SNAREs may partially counteract raft partitioning and thereby restrict their steady‐state localization to endosomal compartments.

Notably, several human SNARE TMDs deviate from the general LASA‐based trends, arguing against a model in which TMD properties alone dictate localization. Indeed, multiple sorting determinants can coexist within a single protein. For example, the yeast SNAREs Sed5 and Ufe1 combine TMD features with cytosolic signals, likely enabling more precise trafficking control [48, 58]. Against this background, the compositional differences observed in SNARE TMDs are best interpreted as contributing to, but generally not being sufficient for, compartment‐specific localization.

Another parameter frequently discussed in the context of membrane protein sorting is TMD length, which is thought to increase by four to nine residues along the secretory pathway, in line with the increase in membrane thickness [18, 29, 34, 40, 47]. According to the hydrophobic matching hypothesis, TMDs preferentially partition into membranes matching their hydrophobic length [20, 29]. This sorting mechanism was suggested to act in lipid rafts, which have thicker membranes than the surrounding disordered region and are thought to enrich long TMDs for PM trafficking [54, 57].

Using sequence‐based hydrophobicity predictions, we observe a gradual increase in predicted TMD length along the secretory pathway, both in the expanded dataset and when considering human SNAREs alone. However, when TMD boundaries are defined by membrane positioning in all‐atom MD simulations, this gradient becomes far less pronounced. This discrepancy reflects fundamental differences between the two approaches.

Sequence‐based methods primarily delineate the most hydrophobic stretch of a transmembrane segment. Charged residues are commonly used to define TMD boundaries and are therefore typically assigned to extra‐membranous regions. However, such assignments do not necessarily reflect their actual positioning within the bilayer. For example, Lys is strongly hydrophilic according to hydropathy scales, yet it contains a long aliphatic side chain that can be accommodated into the hydrophobic region of the membrane while its charged ammonium group remains in contact with lipid phosphates or water. Moreover, lipid bilayers do not form sharp boundaries between aqueous and hydrophobic regions but instead contain interfacial transition zones enriched in polar and charged lipid moieties. Additionally, both proteins and membranes are dynamic, and fluctuations further blur static boundary definitions.

Consistent with this view, MD‐derived TMD boundaries frequently differ from the hydrophobic stretch determined by sequence analysis. The more stringent O–O definition emphasizes the hydrophobic core, whereas the P–P definition includes more of the interfacial region and therefore incorporates additional polar or charged residues into the membrane‐embedded segment. The O–O definition yields highly overlapping TMD length distributions across SNAREs, indicating that the membrane‐embedded segments match the same range of membrane thicknesses. With the P–P definition, most short TMDs are found in the GA, which was also observed for fungal TMDs by Sharpe et al. and aligns with experimental results that indicate that GA membranes are thinner than ER membranes [29, 59]. Importantly, under both definitions, differences in effective membrane‐spanning length between SNARE classes are modest at best compared with the compositional differences described above.

However, we determined TMD lengths in only two relatively similar model membranes, DMPC and DOPC. It therefore remains unclear to what extent our findings can be generalized to other model systems or to biological membranes. Consequently, we cannot exclude the possibility that TMD lengths are modulated differently in other membrane environments. Furthermore, it is well established that sequence‐derived TMD lengths correlate with compartmental localization. The fact that MD‐derived TMD lengths do not reproduce the same trend does not invalidate this relationship but rather indicates that sequence‐derived TMD lengths do not necessarily correspond to the actual membrane‐embedded span, at least in DMPC and DOPC bilayers. This distinction is noteworthy because sequence‐derived TMD length is often implicitly interpreted as the membrane‐spanning length. Consequently, the relationship between TMD length and intracellular sorting may be more nuanced than generally appreciated. Our observations therefore raise the possibility that sequence‐based TMD length estimates primarily reflect differences in amino acid composition rather than substantial differences in membrane‐embedded span. At the same time, membrane properties can influence TMD length, as illustrated by the increase in TMD length observed upon the addition of cholesterol to DOPC bilayers (Figure S16). We therefore cannot exclude the possibility that specific protein‐membrane combinations exist in which the SNAREs examined here exhibit the expected differences in TMD length.

Besides providing insight into TMD lengths, the MD simulations help distinguish between two conceptually different types of charged residues within SNARE TMDs. In Syx5, a Lys residue is positioned close to the membrane‐water interface. In the simulations, its aliphatic portion remains embedded while the charged group snorkels toward the polar headgroup region. This positioning appears to contribute to a defined alignment of the helix within the membrane, suggesting a structural role in orienting the TMD.

In contrast, Vti1b contains a Glu residue located centrally within the hydrophobic core of the TMD. Unlike interfacial Lys, this residue cannot reach the membrane surface and instead induces local rearrangements of surrounding lipids and water penetration in the simulations when charged. Comparable centrally buried charged residues have been described in other fusion‐related membrane proteins, including the HIV‐1 envelope protein and the mitochondrial fusion protein Fzo1, where they modulate helix–helix interactions, lipid packing, and water retention inside the membrane [60, 61]. In case of Fzo1, the packing defects expose hydrophobic lipid tails to the solvent, which is thought to facilitate membrane fusion. By analogy, the conserved Glu in Vti1b may influence TMD organization, SNARE complex assembly, or local membrane properties during fusion. The restricted occurrence of such central charges among SNAREs suggests specialized roles rather than a general principle. At the same time, charged residues within TMDs have been implicated in ER retention and degradation pathways, indicating that such features may also affect localization and turnover [62].

It remains unclear whether our findings are generalizable to other membrane proteins or specific to SNAREs. A comparison with the set of bitopic TMDs compiled in the MembranomeX database indicates that SNARE TMDs are generally enriched in Ile (Figure S21) [63]. This finding is consistent with previous work by Neumann and Langosch [64], which showed that Ile and Val are enriched in SNAREs relative to non‐SNARE proteins. They propose that β‐branched residues such as Ile and Val destabilize α‐helices and thereby enhance helix dynamics to facilitate membrane fusion. Notably, Ile enrichment with respect to non‐SNAREs is observed only in ES and PM TMDs, suggesting that increased Ile content is not a requirement for SNARE function. However, it remains possible that Ile enrichment represents a functional adaptation to the more ordered and tightly packed membranes of the ES and PM, which may not be relevant for SNAREs operating in the ER and GA.

The diverse collection of bitopic TMDs in the MembranomeX dataset only partially recapitulates the compositional trends observed for SNARE TMDs (Figure S22). In particular, amino acid compositions of ER and GA TMDs strongly overlap with those of ES and PM TMDs. While ER and GA TMDs show some enrichment in Phe and ES and PM TMDs in Ile, many other amino acids contribute to the compositional differences. Notably, Ala, Gly, and Val are more enriched in ES and PM TMDs than Ile. However, disentangling the contributions of subcellular localization, protein function, and phylogenetic relationships remains challenging in such heterogeneous datasets. Restricting the analysis to kinases identifies Ile as the most enriched residue in ES and PM TMDs, similar to SNARE TMDs, whereas Ala becomes less prominent than in the complete dataset (Figure S22). However, this analysis is limited by the low abundance of kinases outside the PM. These observations underscore the importance of carefully selecting appropriate protein subsets when analyzing TMD composition across compartments.

Taken together, our results show that SNARE TMDs exhibit systematic differences in amino acid composition that correlate with their site of action, whereas differences in effective membrane‐spanning length are small or non‐existent when membrane embedding is assessed dynamically. By focusing on a single protein family with a conserved core function, our analysis minimizes confounding effects from unrelated evolutionary pressures and highlights a common principle: SNARE TMDs appear tuned to their membrane environments primarily through compositional biases that influence protein‐lipid interactions, while length variations play a comparatively minor role. We therefore propose that TMD composition contributes to functional optimization by modulating energetic compatibility with specific bilayer environments and to compartmental localization, in concert with protein‐based sorting mechanisms.

## 4 Methods

All SNARE sequences were obtained from the TRACEY database (https://tracey.unil.ch; accessed on 08 December 2025) [3, 38, 39], yielding a total of 18 810 sequences. SNAREs are assigned to trafficking steps (Classes I–IV) based on sequence similarity of their SNARE motifs to reference SNAREs with known subcellular localization, using hidden Markov model (HMM) profiles [3]. This classification approach does not bias our analysis, as SNARE localization has evolved independently across the four SNARE types. TMDs were identified using the GES hydrophobicity scale, which specifically accounts for α‐helical structures [42]. Hydropathy scores were calculated using a weighted rolling average with a 15‐residue window: the central residue was fully weighted, while flanking residues were linearly downweighted, reaching zero weight seven positions away from the center. Only TMDs located *C*‐terminally to the SNARE domain and between 10 and 35 residues in length were retained for further analysis. The TMDs of Syx17, which contain two α‐helices, were excluded. This filtering step yielded a total of 13 941 TMD sequences. Amino acid compositions were analyzed using PCA with centering but without scaling, and PLS‐DA with both centering and scaling. PLS‐DA is a supervised dimensionality reduction method that incorporates class labels to enhance group separation and is often considered a supervised analogue of PCA [65].

Because SNARE proteins are represented in unequal numbers in the dataset, potential sampling bias was evaluated. To test whether the observed compositional differences reflect true class‐dependent variation, we performed a more stringent analysis (Figure S23). In this analysis, only Classes I and III were compared, as these are the only classes containing TMDs from all four SNARE types (Qa, Qb, Qc and R). In addition, only unique TMD sequences were included. To eliminate sample‐size bias, 1000 TMDs were randomly drawn (with replacement) from each type‐class combination (e.g., Qa II SNAREs). Results from this reduced dataset closely match those of the full analysis, indicating that the findings are robust. The largest differences in PLS‐DA weights occur for less frequent amino acids, notably Lys and Tyr, which are characteristic of Class II but rare in Class I. Nonetheless, the distinction between Classes I and III remains clearly defined in both PCA (Figure S23C) and PLS‐DA (Figure S23D), with Phe and Ile again serving as the main discriminants.

MD simulations were based on structural models for SNAREs made by ColabFold: AlphaFold2 using MMseqs2 [66]. Only the *C*‐terminal 50 residues of human SNAREs were used to create the structural models (Alignment S1). The only exception was Myob, which was truncated after the TMD due to its extended *C*‐terminus, resulting in a construct of 55 residues. AF2 models for Syx8 and Syx10 displayed pronounced kinks between the SNARE domain and the TMD. These distortions were manually corrected in PyMOL 3.0.5 [67] to prevent the *N*‐terminus from entering the membrane.

The 30 human SNAREs containing a single TMD were embedded in lipid bilayers using Membrane Builder [68, 69] in CHARMM‐GUI [70, 71, 72]. Protein charges were kept at their default protonation states in CHARMM‐GUI, corresponding to standard ionization at neutral pH. For Vti1b, a separate simulation was performed in which the Glu residue within the TMD was protonated. Each system was solvated with 25 Å minimum water height. Bilayers consisted of either DOPC or DMPC lipids (100 lipids per leaflet) arranged in a hexagonal box. Protein orientation was determined using PPM2 [73], which successfully positioned most proteins except Vti1b, Syx6, and Endob. For these three proteins, orientation was manually defined by aligning a vector along the z‐axis between the following residue pairs: Vti1b (L26–F46), Syx6 (W30–V49), and Endob (M26–F44).

For every protein‐lipid setup, three independent simulations were conducted, consisting of CHARMM‐GUI default energy minimization and equilibration steps, followed by a 100 ns production run using GROMACS (2024.1‐spack) [74] at 310 K and CHARMM‐GUI default parameters. Only the second half (51–100 ns) of each trajectory was used for analysis.

MD TMD lengths were calculated based on the 10 lipids closest to the helix in each leaflet. Lipid adjacency was determined by the Cα‐phosphate distance in three dimensions. The phosphorus atoms and acyl oxygens of the selected lipids were used as boundaries to define whether a residue (based on its Cα position) was embedded within the membrane, considering the z‐dimension only. These boundaries are referred to as P–P and O–O. To minimize noise from local fluctuations, a rolling average over five residues was applied to smooth the Cα‐phosphorus and Cα‐oxygen distance profiles. TMD lengths were calculated as the span from the first to the last residue within the boundaries; therefore, they are not always identical to a simple count of Cα atoms within the boundaries in rare cases when a subsequent Cα atom lies outside the boundary again.

The TMDs of Vamp7 and Myob were excluded from the analysis because both proteins contain an extended *C*‐terminal tail. In the case of Vamp7, the tail may adopt a defined fold stabilized by a disulfide bond, as suggested by AF2 (Figure 5H). This tail resides at the membrane‐solvent interface and occasionally inserts into the bilayer. Myob contains a long, relatively hydrophobic tail that begins to penetrate the membrane during MD simulations. In both proteins, TMD lengths fluctuate substantially, making them unsuitable for analysis.

LASAs were calculated from the last frame of each trajectory using a probe radius of 1.88 Å, corresponding to the size of a methylene group within a lipid acyl chain [44]. LASAs were computed for TMDs as defined by the O–O criterion. Residues located within the membrane in at least 50% of all frames were classified as TMD residues. In the case of the Syb2 mutants, LASAs were calculated based on the last frame of MD simulations with a 1 ns production run in a bilayer containing 50 DOPC molecules in each leaflet.

Water molecules within the membrane were quantified using the O–O boundaries, considering a 10 Å radius in the x‐y plane around the Cα atom at the membrane center. All computational analyses were performed in R 4.4.1 [75] using RStudio [76] with the following packages: bio3d [77], ggplot2 [78], ggseqlogo [79], mdatools [80], msa [81], and vanddraabe [82]. Protein structures were visualized in PyMOL 3.0.5 [67], sequences were aligned using AliView 1.28 [83] and molecular structures were drawn using ChemDoodle (Version 12.9.0).

## Author Contributions

**Christian Baumann:** conceptualization, methodology, formal analysis, investigation, writing – original draft, visualization. **Carlos Pulido‐Quetglas:** resources, data curation. **Dirk Fasshauer:** writing – original draft, supervision, funding acquisition.

## Funding

This work was supported by the Swiss National Science Foundation (SNF 182732 and SNF 219549).

## Ethics Statement

The authors have nothing to report.

## Conflicts of Interest

The authors declare no conflicts of interest.

## Supporting information


**Figure S1:** PCA and PLS‐DA of SNARE TMD amino acid composition. (A) PCA of SNARE TMD amino acid composition (centered and scaled). Each point represents one TMD sequence. Class I scores below –1 in both dimensions stem mostly (87.7%) from the R‐SNARE Sec22. (B) Corresponding PCA loadings. (C) Histogram of scores from PLS‐DA separating Classes I and II. The associated feature weights are shown in the upper right. The minor Class I peak is formed by an excess (72.0%) of Sec22. (D) Histogram of scores from PLS‐DA separating Classes III and IV. The associated feature weights are shown in the upper right.
**Figure S2:** Phylogenetic distribution and classification accuracy of SNARE TMDs. (A) Number of SNARE TMD sequences per phylogenetic group. Opisthokonta are shown in purple and Archaeplastida in green. SNARE TMDs not belonging to the major groups within Opisthokonta and Archaeplastida are binned in “Other O.” and “Other A.”, respectively. The number of sequences is: Metazoa (5503), Fungi (4263), other Opisthokonta (519), Viridiplantae (1358), Rhodophyta (46), Glaucocystophyceae (18), SAR (1222), Discoba (507), Amoebozoa (286), Metamonada (120), Haptista (41), Apusozoa (28), Cryptophyceae (21), Malawimonadida (9). (B) Classification accuracy of the PLS‐DA models shown in Figures 2, S3, and S4. (C) Balanced accuracy of the same models. Balanced accuracy was calculated as the mean of sensitivity and specificity, to account for unequal class sizes.
**Figure S3:** PCA and PLS‐DA of Metazoan and fungi SNARE TMD amino acid composition. (A) PCA of SNARE TMD amino acid composition for Metazoa with corresponding loadings. Data points represent individual TMDs: I (1035 sequences), II (997), III (1869), IV (1602). (B–D) PLS‐DA histograms of Metazoa TMDs. (B) Separation of Classes I/II and III/IV. (C) Separation of Classes I and II. The central peaks contain an excess of Qa SNAREs (Syx18 for I and Syx5 for II). The minor Class I peak contains almost exclusively the R SNARE Sec22. (D) Separation of Classes III and IV. Feature weights for each comparison are shown to the right. (E) PCA of SNARE TMD amino acid composition for fungi with corresponding loadings: I (1034 sequences), II (1137), III (1427), IV (665). (F–H) PLS‐DA histograms of fungal TMDs: (F) I/II and III/IV; (G) I and II; (H) III and IV, with feature weights shown to the right.
**Figure S4:** PCA and PLS‐DA of Archaeplastida and SAR SNARE TMD amino acid composition. (A) PCA of SNARE TMD amino acid composition for Archaeplastida with corresponding loadings. Each point represents one TMD: I (365 sequences), II (236), III (622), IV (199). (B–D) PLS‐DA histograms of Archaeplastida TMDs. (B) Separation of Classes I/II and III/IV. (C) Separation of Classes I and II. (D) Separation of Classes III and IV. Feature weights for each comparison are shown to the right. (E) PCA of SAR SNARE TMD amino acid composition with corresponding loadings: I (172), II (217), III (690), IV (143). (F–H) PLS‐DA histograms of SAR TMDs: (F) I/II and III/IV; (G) I and II; (H) III and IV, with feature weights shown to the right.
**Figure S5:** SNARE type‐specific variation in TMD amino acid composition. (A) Mean amino acid composition of SNARE TMDs by SNARE type. Error bars indicate the range containing 80% of all TMDs. (B) Unscaled PCA of SNARE TMD amino acid composition with corresponding PCA loadings. Each point represents one TMD. (C) Scaled PCA of SNARE TMD amino acid composition. (D) Scaled PLS‐DA separating all SNARE types using two principal components. (E) Feature weights contributing to the PLS‐DA separation.
**Figure S6:** SNARE type‐specific variation in TMD amino acid composition of Class I and Class II SNAREs. (A) Mean amino acid composition of Class I SNARE TMDs. Error bars indicate the range containing 80% of all TMDs. (B) Scaled PLS‐DA of Class I SNARE TMD amino acid composition. Each point represents a single TMD. (C) Feature weights contributing to the Class I PLS‐DA separation. (D) Mean amino acid composition of Class II SNARE TMDs. Error bars indicate the range containing 80% of all TMDs. (E) Scaled PLS‐DA of Class II SNARE TMD amino acid composition. Each point represents a single TMD. (F) Feature weights contributing to the Class II PLS‐DA separation. Data were resampled by drawing 2,000 sequences per class with replacement.
**Figure S7:** SNARE type‐specific variation in TMD amino acid composition of Class III and Class IV SNAREs. (A) Mean amino acid composition of Class III SNARE TMDs. Error bars indicate the range containing 80% of all TMDs. (B) Scaled PLS‐DA of Class III SNARE TMD amino acid composition. Each point represents a single TMD. (C) Feature weights contributing to the Class III PLS‐DA separation. (D) Mean amino acid composition of Class IV SNARE TMDs. Error bars indicate the range containing 80% of all TMDs. (E) Scaled PLS‐DA of Class IV SNARE TMD amino acid composition. Each point represents a single TMD. (F) Feature weights contributing to the Class IV PLS‐DA separation. Data were resampled by drawing 2,000 sequences per class with replacement.
**Figure S8:** TMD lengths of Syx5 and Syb2 with modified TMDs. TMDs were truncated and elongated by 4 and 7 residues, which roughly correspond to one and two helical turns. Residues were removed from or added to the center of the TMDs. The wt MD simulation data are identical to those used for the comparison between SNAREs and were performed in triplicates. Elongated Syx5 constructs were simulated in duplicates. PPM 2.0 positioned these TMDs with a strong initial tilt, leading to partial insertion of TMD‐adjacent residues (similar to Figure S19; example frames are shown in Figure S9). To test the influence of the initial positioning in this case, a second MD run was performed using an alternative initial orientation with substantially reduced tilt, positioning the Lys residue in the Syx5 TMD close to the membrane‐water interface (Figure S9). The first trajectories shown for Syx5 +4 and +7 correspond to the PPM 2.0‐derived initial orientation, whereas the second ones correspond to the less tilted initial positioning. The two initial states generally produced distinct MD trajectories. The only exception is Syx5 +4 in DMPC, for which the PPM 2.0 trajectory converged to the alternative conformation. (A) Frequency heatmap of TMD lengths determined from the number of Cα atoms located within the O–O layers in DOPC. Color intensity reflects the frequency of observing a given TMD length. The average TMD length for each trajectory is indicated by circles. (B) Same as in (A) but using DMPC. (C) Correlation between sequence modifications and average MD‐derived TMD length based on the O–O definition, relative to the corresponding wt TMDs. Colors are the same as in Panels A and B. Averages are shown for Syx5 +4 and +7, with individual trajectory averages displayed as separate, lighter‐colored data points. Sequence modifications are largely reflected in the MD‐derived TMD lengths. The strongest deviation is observed for the –7 truncations, most prominently for Syb2 in DOPC, indicating that additional residues enter the O–O layers due to the strong truncation. Nevertheless, with the exception of Syb2 in DOPC, all TMDs can be shortened by more than four residues, highlighting that the wt Syx5 and Syb2 TMDs are not at the lower limit of what the model membranes can accommodate. (D) Same as in (A) but considering the P–P definition. (E) Same as in (B) but considering the P–P definition. (F) Same as in (C) but considering the P–P definition. Deviations associated with the –7 truncation are less pronounced than with the O–O definition. (G) Residues located within the membrane in ≥ 50% of frames are color‐coded in green for the O–O definition and in red for the P–P definition. The most deeply buried charged residues are highlighted in red. Sequence modifications are highlighted in blue, indicating added residues or the sites of residue removal (marked by an asterisk). For Syx5 +4 and +7, averages from both trajectories are shown. Results from the individual trajectories are indicated above the sequence for the PPM 2.0 positioning and below the sequence for the less tilted initial positioning. (H) Same as in (G) but in DMPC.
**Figure S9:** Membrane positioning of K33 in wt and modified Syx5 TMDs. (A) Frequency heatmap of K33 Cα burial depth with respect to the O‐layer. Color intensity reflects the frequency of observing a given burial depth. The average burial depth for each trajectory is indicated by circles. Red asterisks show the initial burial depth of K33 in the MD setup, highlighting that K33 becomes inserted into the membrane when initially positioned above the O‐layer. (B) Same as (A), but showing K33 burial depth with respect to the P‐layer. (C) MD snapshots of Syx5 with wt, truncated (–7), and elongated (+7) TMDs obtained from DOPC simulations to illustrate TMD positioning and the burial of K33. The side chain of K33 is highlighted in red, and R28 is additionally shown in blue, as it also appears to contribute to TMD positioning. For the Syx5 +7 TMD, snapshots from simulations initiated with the two different membrane positions are shown. Conformation 1 was obtained from the initial PPM 2.0 positioning, whereas conformation 2 was obtained from the less tilted initial state.
**Figure S10:** Comparison of TMD lengths from MD simulations and sequence‐based predictions. (A) Correlation of O–O TMD lengths between DOPC and DMPC membranes. Data points indicate averages, and error bars show the 95% confidence intervals based on the three replicates per membrane. PCC is shown. (B) Correlation of P–P TMD lengths between DOPC and DMPC membranes. Averages, 95% confidence intervals, and PCC are shown. (C) Frequency heatmap of the number of *C*‐terminal residues extending beyond the phosphate layer. Color intensity indicates frequency across trajectories; short bars denote averages per trajectory. (D) Average number of *C*‐terminal residues outside the membrane using phosphate and oxygen layers considering both membrane types. Error bars indicate the 95% confidence intervals. Dashed lines correspond to the length of a single residue. (E) Correlation between MD‐derived P–P TMD lengths and sequence‐based lengths obtained using the GES hydrophobicity scale.
**Figure S11:** TMDs determined by MD simulations and the GES scale. The *C*‐terminal 40 residues of each SNARE protein are shown. Curves indicate the fraction of frames in which each residue resides within the membrane across all trajectories in DOPC and DMPC bilayers, using the O–O definition (green) and P–P definition (red). Residues located within the membrane in ≥ 50% of frames are color‐coded in the sequence accordingly (green for O–O, red for P–P). The blue bar highlights residues assigned to the TMD by the GES hydrophobicity scale. Vti1b is shown for both the charged and protonated (GluH) states.
**Figure S12:** Amino acid composition of GES‐ and MD‐defined SNARE TMDs. (A) Mean amino acid composition of human SNARE TMDs when determined using either the GES hydrophobicity scale and MD simulations. (B) Correlation of PLS‐DA PC1 scores between TMDs defined by the O–O definition and the GES scale. PC1 scores were calculated using feature weights from the PLS‐DA of the complete SNARE TMD dataset. Gos28 (1) and Vti1b (2) are highlighted. The dotted line highlights the separation between Classes I/II and III/IV. (C) Correlation of PLS‐DA PC1 scores between TMDs defined by the P–P definition and the GES scale. The dotted line highlights the separation between Classes I/II and III/IV. (D) Correlation of PLS‐DA PC1 scores between TMDs defined by the P–P and O–O definitions. Gos28 (1) and Vti1b (2) are highlighted.
**Figure S13:** TMD tilts of human SNAREs determined from MD simulations using residues within the O–O layer. In the data shown here, the Glu residue in the Vti1b TMD is protonated and thus neutral. Helix tilt angles were calculated based on a smoothed representation of the helix axis: Midpoints between Cα atoms of neighboring residues (i–1 and i+1) were first determined to define a centerline. These coordinates were subsequently smoothed using a three‐residue rolling average to reduce local structural fluctuations. The resulting coordinates were transformed using PCA with centering and without scaling, and the resulting PC1 was taken as the helix axis. The tilt angle was defined as the angle between PC1 and the membrane normal (z‐axis). (A) Frequency heatmap of TMD tilts from MD simulations. For each SNARE, results obtained in DOPC are shown on the left and those in DMPC on the right using complementary colors. Color intensity reflects the frequency of observing a given tilt angle. The average tilt for each trajectory is indicated by a short bar. (B) TMD tilts of human SNAREs obtained from MD simulations in both membrane types. Individual protein values are shown in the background, while class averages are displayed in the foreground; bars indicate standard deviations. (C) Same as in (B), but showing TMD tilts derived from simulations in DOPC only. (D) Same as in (B), but showing TMD tilts derived from simulations in DMPC only. (E) Correlation between TMD tilts obtained from MD simulations in DOPC and DMPC. (F–H) Correlation between TMD tilts from MD simulations in DOPC and (F) GES‐derived TMD lengths, (G) O–O TMD lengths, and (H) P–P TMD lengths. (I–K) Correlation between TMD tilts from MD simulations in DMPC and (I) GES‐derived TMD lengths, (J) O–O TMD lengths, and (K) P–P TMD lengths.
**Figure S14:** TMD curvatures of human SNAREs determined from MD simulations using residues within the O–O layer. In the data shown here, the Glu residue in the Vti1b TMD is protonated and thus neutral. Helix curvature was quantified as the average deviation of the smoothed centerline from the principal helix axis (see Figure S13). Specifically, the perpendicular displacement of each point from the principal axis was calculated as:
$\text{Curvature}=\frac{1}{n}\sum_{i=1}^{n}\sqrt{\mathrm{PC}2_{i}^{2}+\mathrm{PC}3_{i}^{2}}$
This metric reflects the extent to which the helix deviates from an ideal straight axis, with larger values indicating increased curvature. (A) Frequency heatmap of TMD curvatures from MD simulations. For each SNARE, results obtained in DOPC are shown on the left and those in DMPC on the right using complementary colors. Color intensity reflects the frequency of observing a given curvature. The average curvature for each trajectory is indicated by a short bar. (B) TMD curvatures of human SNAREs obtained from MD simulations in both membrane types. Individual protein values are shown in the background, while class averages are displayed in the foreground; bars indicate standard deviations. (C) Same as in (B), but showing TMD curvatures derived from simulations in DOPC only. (D) Same as in (B), but showing TMD curvatures derived from simulations in DMPC only. (E) Correlation between TMD curvatures obtained from MD simulations in DOPC and DMPC. (F–I) Correlation between TMD curvatures from MD simulations in DOPC and (F) GES‐derived TMD lengths, (G) O–O TMD lengths, (H) P–P TMD lengths, and (I) TMD tilt. (J–M) Correlation between TMD curvatures from MD simulations in DMPC and (J) GES‐derived TMD lengths, (K) O–O TMD lengths, (L) P–P TMD lengths, and (M) TMD tilt.
**Figure S15:** TMD curvatures of human SNAREs determined from MD simulations using residues within the O–O layer. In the data shown here, the Glu residue in the Vti1b TMD is deprotonated and therefore charged. Helix curvature was quantified as described in Figure S14. (A) Frequency heatmap of TMD curvatures from MD simulations. For each SNARE, results obtained in DOPC are shown on the left and those in DMPC on the right using complementary colors. Color intensity reflects the frequency of observing a given curvature. The average curvature for each trajectory is indicated by a short bar. (B) Representative snapshots of Vti1b in DOPC with Glu in its neutral (Vti1b GluH) and charged (Vti1b) states, illustrating TMD curvature. Curvature values are given in Å.
**Figure S16:** TMD lengths of a subset of human SNAREs determined from MD simulations in a mixed bilayer containing 60% DOPC and 40% cholesterol. Each leaflet contains 60 DOPC and 40 cholesterol molecules. For the definition of O–O and P–P boundaries, only the six DOPC molecules closest to the TMD helix in each leaflet were considered to account for the reduced DOPC density compared to pure DOPC bilayers. All other calculations were performed as described for DOPC and DMPC bilayers. (A) Frequency heatmap of TMD lengths determined from the number of Cα atoms located within the O–O layers. Color intensity reflects the frequency of observing a given TMD length. The average TMD length for each trajectory is indicated by a circle. (B) Same as in (A), but with TMD lengths defined using the P–P boundaries. (C) TMD tilts of the SNARE subset analyzed in cholesterol‐containing membranes. Each data point represents the trajectory average. DOPC and DMPC simulations were performed in triplicate, resulting in threefold more data points. (D) TMD lengths based on the O–O definition. Trajectory averages are shown. (E) TMD lengths based on the P–P definition. Trajectory averages are shown. (F) Membrane thickness based on the O–O definition. Trajectory averages are shown. (G) Membrane thickness based on the P–P definition. Trajectory averages are shown.
**Figure S17:** Correlations between LASA and amino acid composition in human SNARE TMDs based on the O–O definition: (A) LASA versus Phe content. (B) LASA versus Leu content. (C) LASA versus Ile content. (D) LASA versus Val content. (E) LASA versus PLS‐DA scores. PCCs are indicated at the top of each plot. Highlighted SNAREs: Sec20 (1), Gos28 (2), GS15 (3), Syx6 (4), Syx10 (5), Vti1b (6).
**Figure S18:** Conservation of the *C*‐terminal region of Vamp7 across eukaryotes. (A) Multiple sequence alignment of the *C*‐terminal regions of Vamp7 orthologs across Eukaryota. AF2 structural models are shown for (B) *Homo sapiens* (Obazoa) Vamp7, (C) *Arabidopsis thaliana* (Archaeplastida) Vamp714, (D) *Bigelowiella natans* (SAR) Vamp71, (E) *Acanthamoeba castellanii* (Amoebozoa) Vamp72, and (F) *Naegleria gruberi* (Discoba) Vamp71. Sulfur atoms forming the potentially conserved disulfide bond are highlighted in yellow.
**Figure S19:** Effect of the K33L substitution on Syx5 TMD length and membrane insertion. (A) Frequency heatmap of TMD lengths based on the P–P definition for wt‐ and K33L‐Syx5 trajectories. Color intensity indicates how frequently a given TMD length was observed. Small bars indicate the mean for each trajectory. (B) Same as (A), but using the O–O definition. Most K33L‐Syx5 trajectories display an increase in TMD length due to more residues being inserted on the cytosolic side of the membrane. (C, D) TMDs determined by MD simulations. The *C*‐terminal 40 residues of wt‐Syx5 (C) and K33L‐Syx5 (D) are shown. Curves indicate the fraction of frames in which each residue resides within the membrane across all trajectories, using the O–O (green) and P–P definition (red). Residues located within the membrane in ≥ 50% of frames are color‐coded in the sequence accordingly (green for O–O, red for P–P). (E, F) MD snapshots of wt‐Syx5 (E) and K33L‐Syx5 (F) obtained from simulations with DOPC to illustrate the sampled conformational states. The interfacial Trp residue and the hydrophobic residues of the amphipathic helix are highlighted in blue. The Lys residue in the wt TMD and its Leu substitution are shown in red. The K33L substitution promotes deeper insertion of the TMD into the membrane and induces a pronounced kink within the helix, resulting in partial insertion of the TMD‐adjacent region through the hydrophobic face of the amphipathic helix. These findings suggest that K33 contributes to proper positioning of the TMD within the membrane.
**Figure S20:** Influence of Glu protonation on Vti1b TMD length. (A) Frequency heatmap of TMD lengths based on the P–P definition for all Vti1b trajectories with Glu in its charged and in its neutral (GluH) state. Color intensity indicates how frequently a given TMD length was observed. Small bars indicate the mean for each trajectory. Differences between the two Glu protonation states were not statistically significant (two‐tailed t‐test, *p* = 0.65). (B) Same as (A), but using the O–O definition (*p* = 0.31). One Vti1b (GluH) trajectory in DMPC displays a markedly shorter TMD due to formation of a loop between the TMD and the *N*‐terminus. (C) MD snapshots of Vti1b obtained from DMPC simulations illustrating the sampled conformation states. The loop observed in the second DMPC trajectory (residues 18–RKVTTN–23) is highlighted in dark blue. Similar rearrangements of the *N*‐terminal region occurred in other SNARE simulations, such as the first trajectory of Syx1b in DMPC. Looped or kinked states have also been reported previously.
**Figure S21:** Comparison of amino acid composition between SNARE and non‐SNARE TMDs. MembranomeX data (https://biomembhub.org/membranome/) were retrieved on 11 May 2026. To ensure comparability, TMD boundaries were redefined using the GES hydrophobicity scale, as applied to SNARE proteins in this study. MembranomeX TMDs were assigned to the same classes used for SNAREs based on annotated subcellular localization: Class I (ER, 1958 TMDs), Class II (GA, 1232 TMDs), Class III (ES; including vesicles, endosomes, lysosomes, melanosomes, peroxisomes, and vacuoles; 353 TMDs), and Class IV (PM; 5053 TMDs). SNARE proteins were extracted from the MembranomeX data based on annotations. As a non‐SNARE example, kinase TMDs were extracted from the MembranomeX data based on annotations. Due to the low number of kinases outside the PM, TMDs were resampled with replacement to generate datasets of 5000 sequences each for ER/GA and ES/PM proteins, respectively. (A) Mean amino acid composition of TMDs. Error bars indicate the central 80% range of the distribution. (B–E) Mean amino acid composition of TMDs by subcellular compartment: ER (B), GA (C), ES (D), and PM (E). Error bars indicate the central 80% range of the distribution.
**Figure S22:** Comparison of amino acid composition between SNARE and non‐SNARE TMDs. For MembranomeX data preparation, see Figure S21 legend. (A) Mean amino acid composition of TMDs by protein group and subcellular localization. Error bars indicate the central 80% range of the distribution. (B) PCA of amino acid composition for MembranomeX non‐SNARE TMDs, with corresponding feature loadings. Each point represents a single TMD sequence. (C) Histogram of PLS‐DA results separating Classes I/II and III/IV of MembranomeX non‐SNARE TMDs; feature weights are shown in the upper‐right inset. (D) PCA of amino acid composition for MembranomeX kinase TMDs, with corresponding feature loadings. Each point represents a single TMD sequence. (E) Histogram of PLS‐DA results separating Classes I/II and III/IV of MembranomeX kinase TMDs; feature weights are shown in the upper‐right inset.
**Figure S23:** Subsampling analysis of SNARE TMD amino acid composition. (A) Number of unique SNARE TMD sequences grouped by type (Qa, Qb, Qc, R) and Class (I–IV). Only Class I and III contain all four SNARE types and were therefore used for this analysis. For each type‐class combination (e.g., Qa II), 1,000 TMDs were randomly sampled with replacement. (B) Mean amino acid composition of Class I and Class III SNARE TMDs. TMDs were defined using the GES hydrophobicity scale. Error bars indicate the range containing 80% of all TMDs. (C) PCA of SNARE TMD amino acid composition with corresponding relative loadings. Each point represents one TMD sequence. (D) Histogram of PLS‐DA results separating Classes I/II and III/IV, with feature weights shown in the upper‐right panel.
**Alignment S1**. *C*‐terminal sequences of human SNAREs used for MD simulations in this study.

## Data Availability

The data that support the findings of this study are openly available in the TRACEY database at https://tracey.unil.ch. This database is a collection of sequences from different protein families, including SNARE, AAA, and Rab, classified by HMM profiles. The MD simulation data that support the findings of this study are available from the corresponding author upon reasonable request.

## References

[1] M. Doktorova , J. L. Symons , X. Zhang , et al., “Cell Membranes Sustain Phospholipid Imbalance via Cholesterol Asymmetry,” Cell 188, no. 10 (2025): 2586–2602e2524, 10.1016/j.cell.2025.02.034. PMC12085300 40179882

[2] I. D. Pogozheva , G. A. Armstrong , L. Kong , et al., “Comparative Molecular Dynamics Simulation Studies of Realistic Eukaryotic, Prokaryotic, and Archaeal Membranes,” Journal of Chemical Information and Modeling 62, no. 4 (2022): 1036–1051, 10.1021/acs.jcim.1c01514. 35167752

[3] T. H. Kloepper , C. N. Kienle , and D. Fasshauer , “An Elaborate Classification of SNARE Proteins Sheds Light on the Conservation of the Eukaryotic Endomembrane System,” Molecular Biology of the Cell 18, no. 9 (2007): 3463–3471, 10.1091/mbc.e07-03-0193. PMC1951749 17596510

[4] T. H. Kloepper , C. N. Kienle , and D. Fasshauer , “SNAREing the Basis of Multicellularity: Consequences of Protein Family Expansion During Evolution,” Molecular Biology and Evolution 25, no. 9 (2008): 2055–2068, 10.1093/molbev/msn151. 18621745

[5] I. Dingjan , P. T. A. Linders , D. R. J. Verboogen , N. H. Revelo , M. Ter Beest , and G. van den Bogaart , “Endosomal and Phagosomal SNAREs,” Physiological Reviews 98, no. 3 (2018): 1465–1492, 10.1152/physrev.00037.2017. 29790818

[6] K. More , C. M. Klinger , L. D. Barlow , and J. B. Dacks , “Evolution and Natural History of Membrane Trafficking in Eukaryotes,” Current Biology 30, no. 10 (2020): R553–R564, 10.1016/j.cub.2020.03.068. 32428497

[7] R. Jahn and R. H. Scheller , “SNAREs–Engines for Membrane Fusion,” Nature Reviews Molecular Cell Biology 7, no. 9 (2006): 631–643, 10.1038/nrm2002. 16912714

[8] R. Jahn , D. C. Cafiso , and L. K. Tamm , “Mechanisms of SNARE Proteins in Membrane Fusion,” Nature Reviews. Molecular Cell Biology 25, no. 2 (2024): 101–118, 10.1038/s41580-023-00668-x. PMC11578640 37848589

[9] D. Fasshauer , R. B. Sutton , A. T. Brunger , and R. Jahn , “Conserved Structural Features of the Synaptic Fusion Complex: SNARE Proteins ReClassified as Q‐ and R‐SNAREs,” Proceedings of the National Academy of Sciences of the United States of America 95, no. 26 (1998): 15781–15786, 10.1073/pnas.95.26.15781. PMC28121 9861047

[10] D. Yadav , A. Hacisuleyman , M. Dergai , et al., “A Look Beyond the QR Code of SNARE Proteins,” Protein Science 33, no. 9 (2024): e5158. 10.1002/pro.5158 PMC11344281 39180485

[11] E. Itakura and N. Mizushima , “Syntaxin 17: The Autophagosomal SNARE,” Autophagy 9, no. 6 (2013): 917–919, 10.4161/auto.24109. PMC3672300 23466629

[12] R. S. Hegde and R. J. Keenan , “Tail‐Anchored Membrane Protein Insertion Into the Endoplasmic Reticulum,” Nature Reviews. Molecular Cell Biology 12, no. 12 (2011): 787–798, 10.1038/nrm3226. PMC3760496 22086371

[13] U. S. Chio , H. Cho , and S. O. Shan , “Mechanisms of Tail‐Anchored Membrane Protein Targeting and Insertion,” Annual Review of Cell and Developmental Biology 33 (2017): 417–438, 10.1146/annurev-cellbio-100616-060839. PMC6343671 28992441

[14] S. Koike and R. Jahn , “SNARE Proteins: Zip Codes in Vesicle Targeting?,” Biochemical Journal 479, no. 3 (2022): 273–288, 10.1042/BCJ20210719. PMC8883487 35119456

[15] K. W. Downes and G. Zanetti , “Mechanisms of COPII Coat Assembly and Cargo Recognition in the Secretory Pathway,” Nature Reviews. Molecular Cell Biology 26, no. 12 (2025): 910–925, 10.1038/s41580-025-00839-y. PMC7617623 40133632

[16] D. J. Sweet and H. R. Pelham , “The *Saccharomyces cerevisiae* SEC20 Gene Encodes a Membrane Glycoprotein Which Is Sorted by the HDEL Retrieval System,” EMBO Journal 11, no. 2 (1992): 423–432, 10.1002/j.1460-2075.1992.tb05071.x. PMC556471 1537327

[17] S. Newstead and F. Barr , “Molecular Basis for KDEL‐Mediated Retrieval of Escaped ER‐Resident Proteins ‐ SWEET Talking the COPs,” Journal of Cell Science 133, no. 19 (2020): 1–10, 10.1242/jcs.250100. PMC7561476 33037041

[18] R. T. Watson and J. E. Pessin , “Transmembrane Domain Length Determines Intracellular Membrane Compartment Localization of Syntaxins 3, 4, and 5,” American Journal of Physiology. Cell Physiology 281, no. 1 (2001): C215–C223, 10.1152/ajpcell.2001.281.1.C215. 11401844

[19] R. Quiroga , A. Trenchi , A. Gonzalez Montoro , J. Valdez Taubas , and H. J. Maccioni , “Short Transmembrane Domains With High‐Volume Exoplasmic Halves Determine Retention of Type II Membrane Proteins in the Golgi Complex,” Journal of Cell Science 126, no. Pt 23 (2013): 5344–5349, 10.1242/jcs.130658. 24105265

[20] D. Milovanovic , A. Honigmann , S. Koike , et al., “Hydrophobic Mismatch Sorts SNARE Proteins Into Distinct Membrane Domains,” Nature Communications 6 (2015): 5984, 10.1038/ncomms6984. PMC4313621 25635869

[21] C. Bian , A. Marchetti , M. Dias , J. Perrin , and P. Cosson , “Short Transmembrane Domains Target Type II Proteins to the Golgi Apparatus and Type I Proteins to the Endoplasmic Reticulum,” Journal of Cell Science 137, no. 15 (2024): 1–8, 10.1242/jcs.261738. 38973735

[22] T. Dingjan and A. H. Futerman , “The Fine‐Tuning of Cell Membrane Lipid Bilayers Accentuates Their Compositional Complexity,” BioEssays 43, no. 5 (2021): e2100021, 10.1002/bies.202100021. 33656770

[23] T. Harayama and H. Riezman , “Understanding the Diversity of Membrane Lipid Composition,” Nature Reviews. Molecular Cell Biology 19, no. 5 (2018): 281–296, 10.1038/nrm.2017.138. 29410529

[24] J. Bigay and B. Antonny , “Curvature, Lipid Packing, and Electrostatics of Membrane Organelles: Defining Cellular Territories in Determining Specificity,” Developmental Cell 23, no. 5 (2012): 886–895, 10.1016/j.devcel.2012.10.009. 23153485

[25] J. C. Holthuis and A. K. Menon , “Lipid Landscapes and Pipelines in Membrane Homeostasis,” Nature 510, no. 7503 (2014): 48–57, 10.1038/nature13474. 24899304

[26] B. Antonny , S. Vanni , H. Shindou , and T. Ferreira , “From Zero to Six Double Bonds: Phospholipid Unsaturation and Organelle Function,” Trends in Cell Biology 25, no. 7 (2015): 427–436, 10.1016/j.tcb.2015.03.004. 25906908

[27] A. K. Menon , “Sterol Gradients in Cells,” Current Opinion in Cell Biology 53 (2018): 37–43, 10.1016/j.ceb.2018.04.012. 29783105

[28] I. Levental and E. Lyman , “Regulation of Membrane Protein Structure and Function by Their Lipid Nano‐Environment,” Nature Reviews. Molecular Cell Biology 24, no. 2 (2023): 107–122, 10.1038/s41580-022-00524-4. PMC9892264 36056103

[29] H. J. Sharpe , T. J. Stevens , and S. Munro , “A Comprehensive Comparison of Transmembrane Domains Reveals Organelle‐Specific Properties,” Cell 142, no. 1 (2010): 158–169, 10.1016/j.cell.2010.05.037. PMC2928124 20603021

[30] C. Baeza‐Delgado , M. A. Marti‐Renom , and I. Mingarro , “Structure‐Based Statistical Analysis of Transmembrane Helices,” European Biophysics Journal 42, no. 2–3 (2013): 199–207, 10.1007/s00249-012-0813-9. 22588483

[31] I. D. Pogozheva and A. L. Lomize , “Evolution and Adaptation of Single‐Pass Transmembrane Proteins,” Biochimica et Biophysica Acta ‐ Biomembranes 1860, no. 2 (2018): 364–377, 10.1016/j.bbamem.2017.11.002. 29129605

[32] M. Makarova , M. Peter , G. Balogh , et al., “Delineating the Rules for Structural Adaptation of Membrane‐Associated Proteins to Evolutionary Changes in Membrane Lipidome,” Current Biology 30, no. 3 (2020): 367–380e368, 10.1016/j.cub.2019.11.043. PMC6997885 31956022

[33] G. von Heijne , “The Distribution of Positively Charged Residues in Bacterial Inner Membrane Proteins Correlates With the Trans‐Membrane Topology,” EMBO Journal 5, no. 11 (1986): 3021–3027, 10.1002/j.1460-2075.1986.tb04601.x. PMC1167256 16453726

[34] N. Nikolovski , D. Rubtsov , M. P. Segura , et al., “Putative Glycosyltransferases and Other Plant Golgi Apparatus Proteins Are Revealed by LOPIT Proteomics,” Plant Physiology 160, no. 2 (2012): 1037–1051, 10.1104/pp.112.204263. PMC3461528 22923678

[35] J. A. Baker , W. C. Wong , B. Eisenhaber , J. Warwicker , and F. Eisenhaber , “Charged Residues Next to Transmembrane Regions Revisited: “Positive‐Inside Rule” Is Complemented by the “Negative Inside Depletion/Outside Enrichment Rule”,” BMC Biology 15, no. 1 (2017): 66, 10.1186/s12915-017-0404-4. PMC5525207 28738801

[36] J. H. Lorent , K. R. Levental , L. Ganesan , et al., “Plasma Membranes Are Asymmetric in Lipid Unsaturation, Packing and Protein Shape,” Nature Chemical Biology 16, no. 6 (2020): 644–652, 10.1038/s41589-020-0529-6. PMC7246138 32367017

[37] E. Neveu , D. Khalifeh , N. Salamin , and D. Fasshauer , “Prototypic SNARE Proteins Are Encoded in the Genomes of Heimdallarchaeota, Potentially Bridging the Gap Between the Prokaryotes and Eukaryotes,” Current Biology 30, no. 13 (2020): 2468–2480e2465, 10.1016/j.cub.2020.04.060. 32442459

[38] N. Kienle , T. H. Kloepper , and D. Fasshauer , “Phylogeny of the SNARE Vesicle Fusion Machinery Yields Insights Into the Conservation of the Secretory Pathway in Fungi,” BMC Evolutionary Biology 9 (2009): 19, 10.1186/1471-2148-9-19. PMC2639358 19166604

[39] C. Pulido‐Quetglas and D. Fasshauer , “TRACEY: An Updated Resource for SNARE Protein Domain Annotation With Improved HMMs and Expanded Sequence Coverage,” bioRxiv: the preprint server for biology (2026): 2026.2006.2008.729283.

[40] S. Munro , “A Comparison of the Transmembrane Domains of Golgi and Plasma Membrane Proteins,” Biochemical Society Transactions 23, no. 3 (1995): 527–530, 10.1042/bst0230527. 8566407

[41] J. Koehler , N. Woetzel , R. Staritzbichler , C. R. Sanders , and J. Meiler , “A Unified Hydrophobicity Scale for Multispan Membrane Proteins,” Proteins 76, no. 1 (2009): 13–29, 10.1002/prot.22315. PMC2761718 19089980

[42] D. M. Engelman , T. A. Steitz , and A. Goldman , “Identifying Nonpolar Transbilayer Helices in Amino Acid Sequences of Membrane Proteins,” Annual Review of Biophysics and Biophysical Chemistry 15, no. 1 (1986): 321–353, 10.1146/annurev.bb.15.060186.001541. 3521657

[43] M. Medina , Y. T. Chang , H. Rahmani , et al., “Surface Morphometrics Reveals Local Membrane Thickness Variation in Organellar Subcompartments,” Journal of Cell Biology 225, no. 3 (2026), 10.1083/jcb.202505059. PMC12755900 41474626

[44] C. M. Deber and T. A. Stone , “Relative Role(s) of Leucine Versus Isoleucine in the Folding of Membrane Proteins,” Peptide Science 111, no. 1 (2019): e24075.

[45] E. Strandberg and J. A. Killian , “Snorkeling of Lysine Side Chains in Transmembrane Helices: How Easy Can It Get?,” FEBS Letters 544, no. 1–3 (2003): 69–73, 10.1016/S0014-5793(03)00475-7. 12782292

[46] A. Panahi and C. L. Brooks, 3rd , “Membrane Environment Modulates the pKa Values of Transmembrane Helices,” Journal of Physical Chemistry B 119, no. 13 (2015): 4601–4607, 10.1021/acs.jpcb.5b00289. PMC4404502 25734901

[47] M. S. Bretscher and S. Munro , “Cholesterol and the Golgi Apparatus,” Science 261, no. 5126 (1993): 1280–1281, 10.1126/science.8362242. 8362242

[48] D. K. Banfield , M. J. Lewis , C. Rabouille , G. Warren , and H. R. Pelham , “Localization of Sed5, a Putative Vesicle Targeting Molecule, to the Cis‐Golgi Network Involves Both Its Transmembrane and Cytoplasmic Domains,” Journal of Cell Biology 127, no. 2 (1994): 357–371, 10.1083/jcb.127.2.357. PMC2120199 7929581

[49] S. Munro , “An Investigation of the Role of Transmembrane Domains in Golgi Protein Retention,” EMBO Journal 14, no. 19 (1995): 4695–4704, 10.1002/j.1460-2075.1995.tb00151.x. PMC394566 7588599

[50] M. Steegmaier , J. Klumperman , D. L. Foletti , J. S. Yoo , and R. H. Scheller , “Vesicle‐Associated Membrane Protein 4 Is Implicated in Trans‐Golgi Network Vesicle Trafficking,” Molecular Biology of the Cell 10, no. 6 (1999): 1957–1972, 10.1091/mbc.10.6.1957. PMC25394 10359608

[51] J. D. Mancias and J. Goldberg , “The Transport Signal on Sec22 for Packaging Into COPII‐Coated Vesicles Is a Conformational Epitope,” Molecular Cell 26, no. 3 (2007): 403–414, 10.1016/j.molcel.2007.03.017. 17499046

[52] P. Cosson , J. Perrin , and J. S. Bonifacino , “Anchors Aweigh: Protein Localization and Transport Mediated by Transmembrane Domains,” Trends in Cell Biology 23, no. 10 (2013): 511–517, 10.1016/j.tcb.2013.05.005. PMC3783643 23806646

[53] M. A. Surma , C. Klose , and K. Simons , “Lipid‐Dependent Protein Sorting at the Trans‐Golgi Network,” Biochimica et Biophysica Acta 1821, no. 8 (2012): 1059–1067, 10.1016/j.bbalip.2011.12.008. 22230596

[54] B. B. Diaz‐Rohrer , K. R. Levental , K. Simons , and I. Levental , “Membrane Raft Association Is a Determinant of Plasma Membrane Localization,” Proceedings of the National Academy of Sciences of the United States of America 111, no. 23 (2014): 8500–8505, 10.1073/pnas.1404582111. PMC4060687 24912166

[55] B. Diaz‐Rohrer , K. R. Levental , and I. Levental , “Rafting Through Traffic: Membrane Domains in Cellular Logistics,” Biochimica et Biophysica Acta 1838, no. 12 (2014): 3003–3013, 10.1016/j.bbamem.2014.07.029. 25130318

[56] I. Levental , K. R. Levental , and F. A. Heberle , “Lipid Rafts: Controversies Resolved, Mysteries Remain,” Trends in Cell Biology 30, no. 5 (2020): 341–353, 10.1016/j.tcb.2020.01.009. PMC7798360 32302547

[57] J. H. Lorent , B. Diaz‐Rohrer , X. Lin , et al., “Structural Determinants and Functional Consequences of Protein Affinity for Membrane Rafts,” Nature Communications 8, no. 1 (2017): 1219, 10.1038/s41467-017-01328-3. PMC5663905 29089556

[58] J. C. Rayner and H. R. Pelham , “Transmembrane Domain‐Dependent Sorting of Proteins to the ER and Plasma Membrane in Yeast,” EMBO Journal 16, no. 8 (1997): 1832–1841, 10.1093/emboj/16.8.1832. PMC1169786 9155009

[59] D. Glushkova , S. Bohm , and M. Beck , “Systematic Membrane Thickness Variation Across Cellular Organelles Revealed by Cryo‐ET,” Journal of Cell Biology 225, no. 1 (2026), 10.1083/jcb.202504053. PMC12584875 41186573

[60] J. Dev , D. Park , Q. Fu , et al., “Structural Basis for Membrane Anchoring of HIV‐1 Envelope Spike,” Science 353, no. 6295 (2016): 172–175, 10.1126/science.aaf7066. PMC5085267 27338706

[61] R. Versini , M. Baaden , L. Cavellini , M. M. Cohen , A. Taly , and P. F. J. Fuchs , “Lys716 in the Transmembrane Domain of Yeast Mitofusin Fzo1 Modulates Anchoring and Fusion,” Structure 32, no. 11 (2024): 1997–2012e1997, 10.1016/j.str.2024.08.017. 39299234

[62] J. S. Bonifacino , P. Cosson , N. Shah , and R. D. Klausner , “Role of Potentially Charged Transmembrane Residues in Targeting Proteins for Retention and Degradation Within the Endoplasmic Reticulum,” EMBO Journal 10, no. 10 (1991): 2783–2793, 10.1002/j.1460-2075.1991.tb07827.x. PMC452987 1915263

[63] A. L. Lomize , K. A. Schnitzer , S. C. Todd , et al., “Membranome 3.0: Database of Single‐Pass Membrane Proteins With AlphaFold Models,” Protein Science 31, no. 5 (2022): e4318, 10.1002/pro.4318. PMC9047035 35481632

[64] S. Neumann and D. Langosch , “Conserved Conformational Dynamics of Membrane Fusion Protein Transmembrane Domains and Flanking Regions Indicated by Sequence Statistics,” Proteins 79, no. 8 (2011): 2418–2427, 10.1002/prot.23063. 21633971

[65] D. Ruiz‐Perez , H. Guan , P. Madhivanan , K. Mathee , and G. Narasimhan , “So You Think You Can PLS‐DA?,” BMC Bioinformatics 21, no. Suppl 1 (2020): 2, 10.1186/s12859-019-3310-7. PMC7724830 33297937

[66] M. Mirdita , K. Schutze , Y. Moriwaki , L. Heo , S. Ovchinnikov , and M. Steinegger , “ColabFold: Making Protein Folding Accessible to All,” Nature Methods 19, no. 6 (2022): 679–682, 10.1038/s41592-022-01488-1. PMC9184281 35637307

[67] L. Schrödinger , “The PyMOL Molecular Graphics System” (2024).

[68] S. Jo , T. Kim , and W. Im , “Automated Builder and Database of Protein/Membrane Complexes for Molecular Dynamics Simulations,” PLoS One 2, no. 9 (2007): e880, 10.1371/journal.pone.0000880. PMC1963319 17849009

[69] E. L. Wu , X. Cheng , S. Jo , et al., “CHARMM‐GUI Membrane Builder Toward Realistic Biological Membrane Simulations,” Journal of Computational Chemistry 35, no. 27 (2014): 1997–2004, 10.1002/jcc.23702. PMC4165794 25130509

[70] S. Jo , T. Kim , V. G. Iyer , and W. Im , “CHARMM‐GUI: A Web‐Based Graphical User Interface for CHARMM,” Journal of Computational Chemistry 29, no. 11 (2008): 1859–1865, 10.1002/jcc.20945. 18351591

[71] B. R. Brooks , C. L. Brooks, 3rd , A. D. Mackerell, Jr. , et al., “CHARMM: The Biomolecular Simulation Program,” Journal of Computational Chemistry 30, no. 10 (2009): 1545–1614, 10.1002/jcc.21287. PMC2810661 19444816

[72] J. Lee , X. Cheng , J. M. Swails , et al., “CHARMM‐GUI Input Generator for NAMD, GROMACS, AMBER, OpenMM, and CHARMM/OpenMM Simulations Using the CHARMM36 Additive Force Field,” Journal of Chemical Theory and Computation 12, no. 1 (2016): 405–413, 10.1021/acs.jctc.5b00935. PMC4712441 26631602

[73] M. A. Lomize , I. D. Pogozheva , H. Joo , H. I. Mosberg , and A. L. Lomize , “OPM Database and PPM Web Server: Resources for Positioning of Proteins in Membranes,” Nucleic Acids Research 40, no. Database issue (2012): D370–D376, 10.1093/nar/gkr703. PMC3245162 21890895

[74] M. J. Abraham , T. Murtola , R. Schulz , et al., “GROMACS: High Performance Molecular Simulations Through Multi‐Level Parallelism From Laptops to Supercomputers,” SoftwareX 1 (2015): 19–25.

[75] R‐Core‐Team , R: A Language and Environment for Statistical Computing ( *R foundation for statistical computing* , 2024).

[76] Posit‐team , RStudio: Integrated Development Environment for R (2024).

[77] B. J. Grant , A. P. Rodrigues , K. M. ElSawy , J. A. McCammon , and L. S. Caves , “Bio3d: An R Package for the Comparative Analysis of Protein Structures,” Bioinformatics 22, no. 21 (2006): 2695–2696, 10.1093/bioinformatics/btl461. 16940322

[78] H. Wickham , ggplot2: Elegant Graphics for Data Analysis (Springer International Publishing, 2016).

[79] O. Wagih , ggseqlogo: A ‘ggplot2’ Extension for Drawing Publication‐Ready Sequence Logos (2024).

[80] S. Kucheryavskiy , “Mdatools–R Package for Chemometrics,” Chemometrics and Intelligent Laboratory Systems 198 (2020): 103937, 10.1016/j.chemolab.2020.103937.

[81] U. Bodenhofer , E. Bonatesta , C. Horejs‐Kainrath , and S. Hochreiter , “Msa: An R Package for Multiple Sequence Alignment,” Bioinformatics 31, no. 24 (2015): 3997–3999, 10.1093/bioinformatics/btv494. 26315911

[82] E. X. Esposito , “Vanddraabe: Identification and Statistical Analysis of Conserved Waters Near Proteins” (2025).

[83] A. Larsson , “AliView: A Fast and Lightweight Alignment Viewer and Editor for Large Datasets,” Bioinformatics 30, no. 22 (2014): 3276–3278, 10.1093/bioinformatics/btu531. PMC4221126 25095880

