# Supplementary material for: Transmembrane Domain Composition Reflects Subcellular Localization of SNARE Proteins
Source: Traffic. 2026 Nov 9;27(4):e70059. doi: 10.1111/tra.70059 (PMC13748404; doi:10.1111/tra.70059)
Supplement: Supplementary file 1 [file TRA-27-e70059-s001.pdf]

Supplementary Material

Transmembrane domain composition reflects subcellular localization of SNARE proteins

Christian Baumann, Carlos Pulido-Quetglas, Dirk Fasshauer

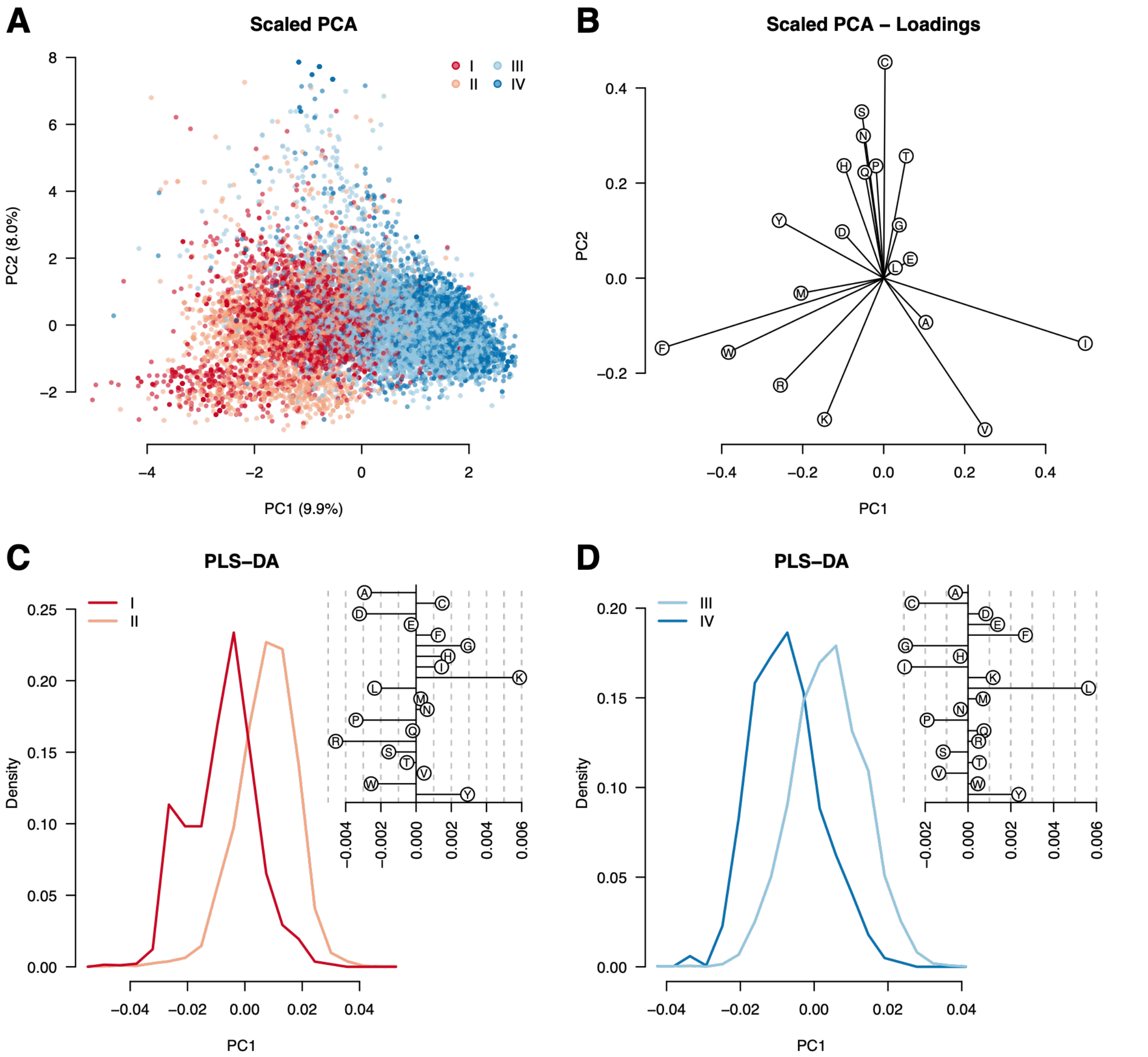

Figure S1. PCA and PLS-DA of SNARE TMD amino acid composition. **(A)** PCA of SNARE TMD amino acid composition (centered and scaled). Each point represents one TMD sequence. Class I scores below −1 in both dimensions stem mostly (87.7%) from the R-SNARE Sec22. **(B)** Corresponding PCA loadings. **(C)** Histogram of scores from PLS-DA separating classes I and II. The associated feature weights are shown in the upper right. The minor class I peak is formed by an excess (72.0%) of Sec22. **(D)** Histogram of scores from PLS-DA separating classes III and IV. The associated feature weights are shown in the upper right.

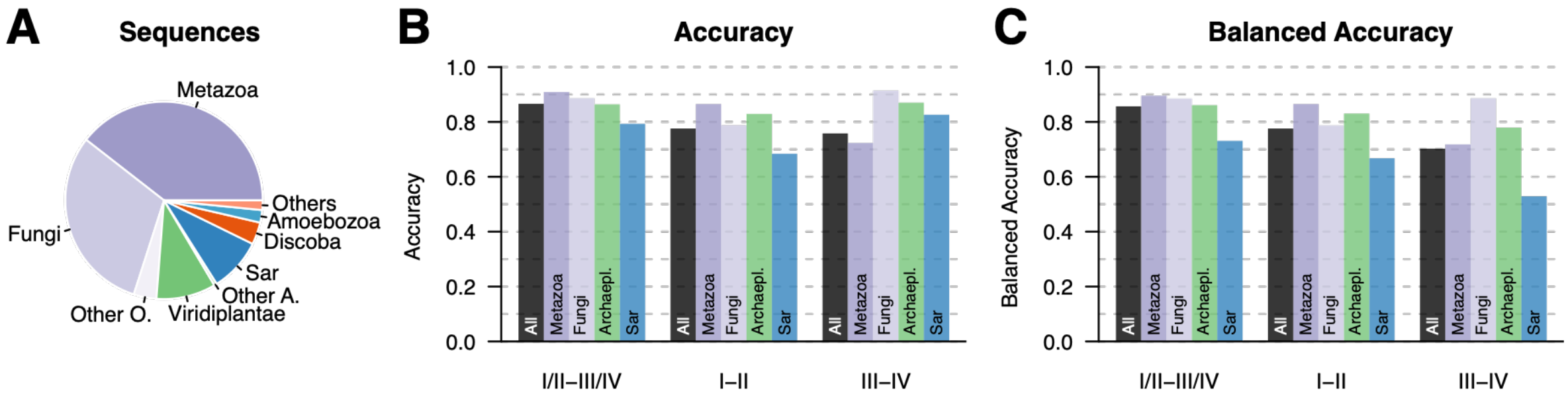

Figure S2. Phylogenetic distribution and classification accuracy of SNARE TMDs. **(A)** Number of SNARE TMD sequences per phylogenetic group. Opisthokonta are shown in purple and Archaeplastida in green. SNARE TMDs not belonging to the major groups within Opisthokonta and Archaeplastida are binned in "Other O." and "Other A.", respectively. The number of sequences is: Metazoa (5503), Fungi (4263), other Opisthokonta (519), Viridiplantae (1358), Rhodophyta (46), Glaucocystophyceae (18), SAR (1222), Discoba (507), Amoebozoa (286), Metamonada (120), Haptista (41), Apusozoa (28), Cryptophyceae (21), Malawimonadida (9). **(B)** Classification accuracy of the PLS-DA models shown in Figures 2, S3, and S4. **(C)** Balanced accuracy of the same models. Balanced accuracy was calculated as the mean of sensitivity and specificity, to account for unequal class sizes.

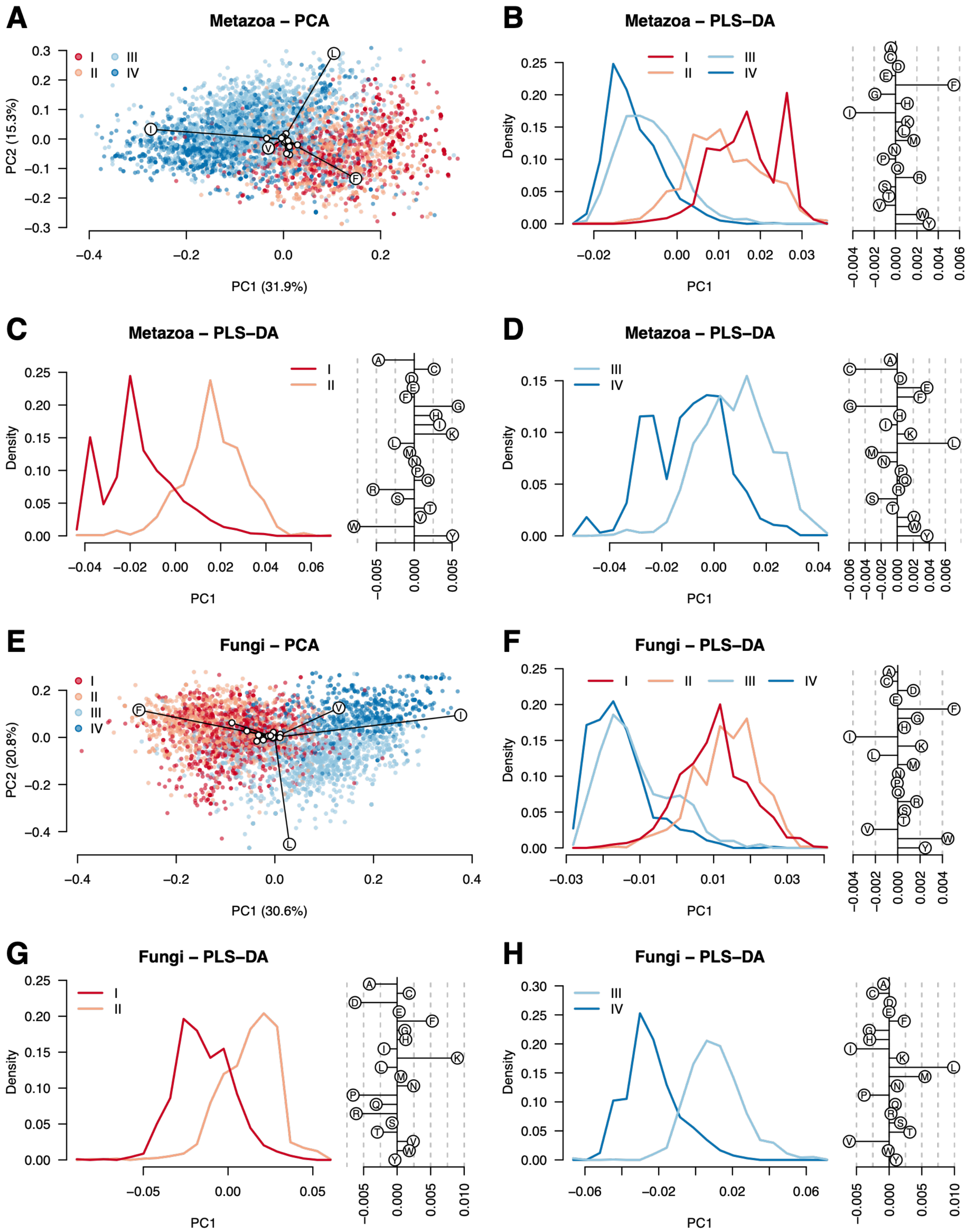

Figure S3. PCA and PLS-DA of Metazoan and fungi SNARE TMD amino acid composition. **(A)** PCA of SNARE TMD amino acid composition for Metazoa with corresponding loadings. Data points represent individual TMDs: I (1035 sequences), II (997), III (1869), IV (1602). **(B–D)** PLS-DA histograms of Metazoa TMDs. (B) Separation of classes I/II and III/IV. (C) Separation of classes I and II. The central peaks contain an excess of Qa SNAREs (Syx18 for I and Syx5 for II). The minor class I peak contains almost exclusively the R SNARE Sec22. (D) Separation of classes III and IV. Feature weights for each comparison are shown to the right. **(E)** PCA of SNARE TMD amino acid composition for fungi with corresponding loadings: I (1034 sequences), II (1137), III (1427), IV (665). **(F–H)** PLS-DA histograms of fungal TMDs: (F) I/II and III/IV; (G) I and II; (H) III and IV, with feature weights shown to the right.

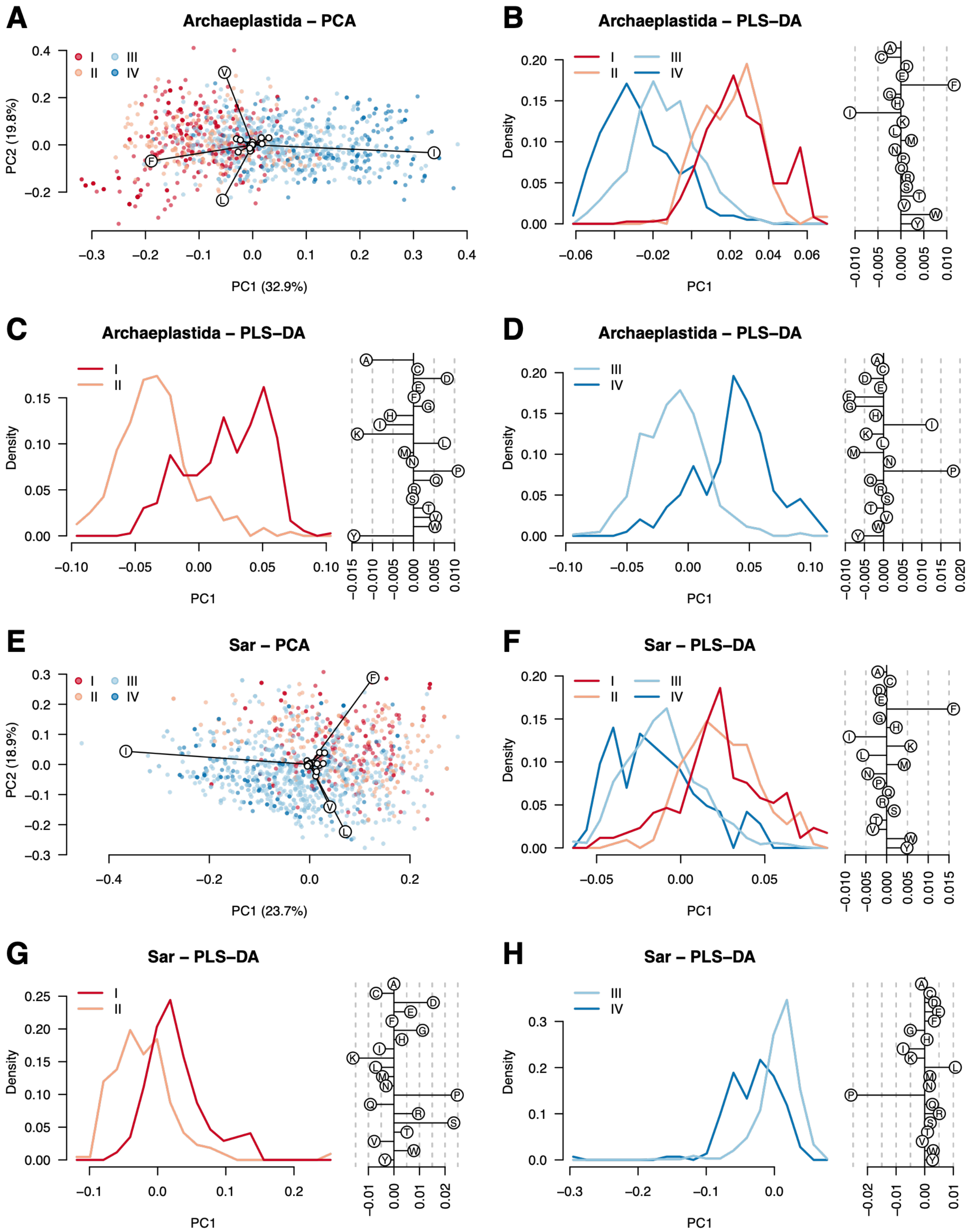

Figure S4. PCA and PLS-DA of Archaeplastida and SAR SNARE TMD amino acid composition. **(A)** PCA of SNARE TMD amino acid composition for Archaeplastida with corresponding loadings. Each point represents one TMD: I (365 sequences), II (236), III (622), IV (199). **(B–D)** PLS-DA histograms of Archaeplastida TMDs. (B) Separation of classes I/II and III/IV. (C) Separation of classes I and II. (D) Separation of classes III and IV. Feature weights for each comparison are shown to the right. **(E)** PCA of SAR SNARE TMD amino acid composition with corresponding loadings: I (172), II (217), III (690), IV (143). **(F–H)** PLS-DA histograms of SAR TMDs: (F) I/II and III/IV; (G) I and II; (H) III and IV, with feature weights shown to the right.

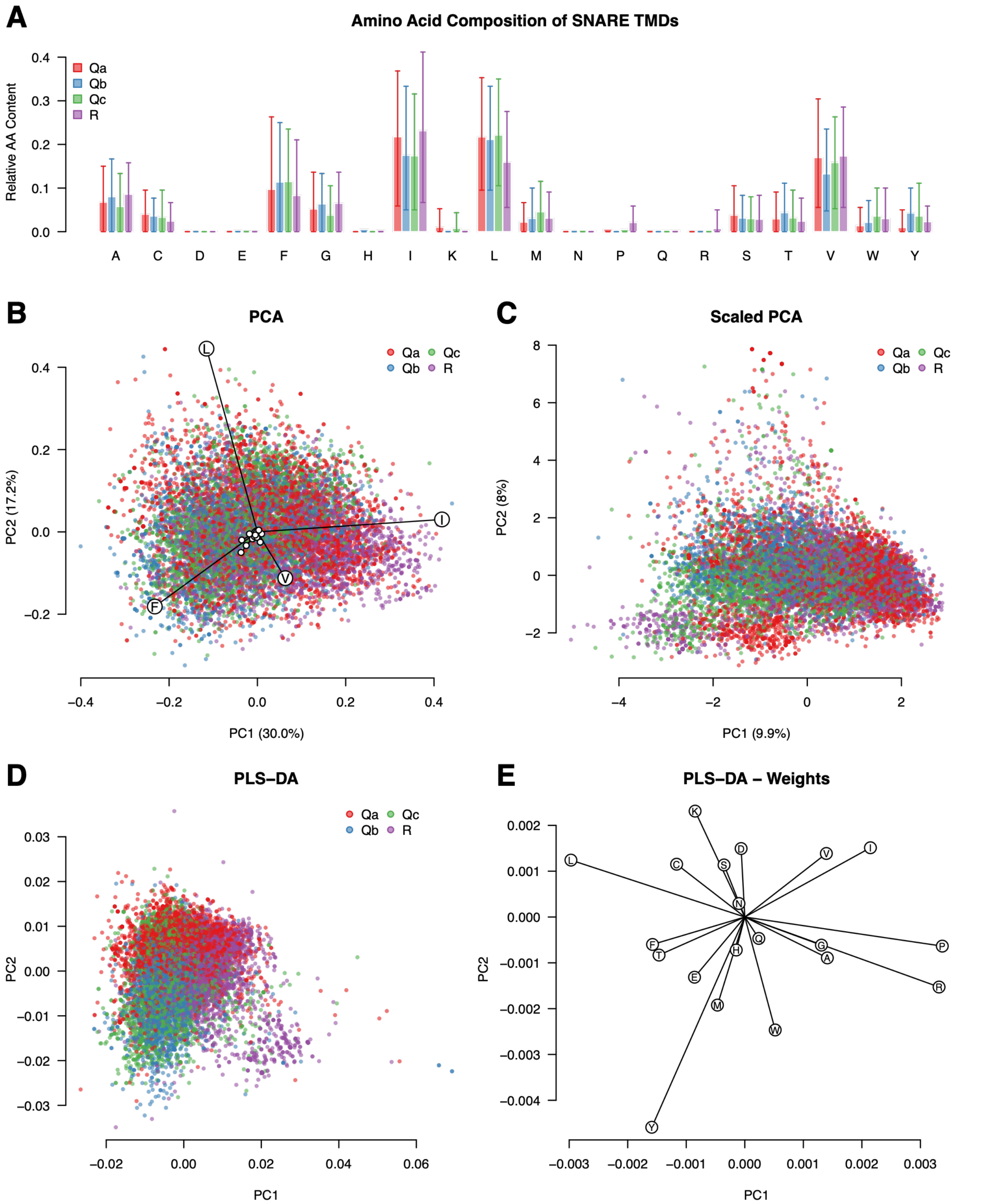

Figure S5. SNARE type-specific variation in TMD amino acid composition. **(A)** Mean amino acid composition of SNARE TMDs by SNARE type. Error bars indicate the range containing 80% of all TMDs. **(B)** Unscaled PCA of SNARE TMD amino acid composition with corresponding PCA loadings. Each point represents one TMD. **(C)** Scaled PCA of SNARE TMD amino acid composition. **(D)** Scaled PLS-DA separating all SNARE types using two principal components. **(E)** Feature weights contributing to the PLS-DA separation.

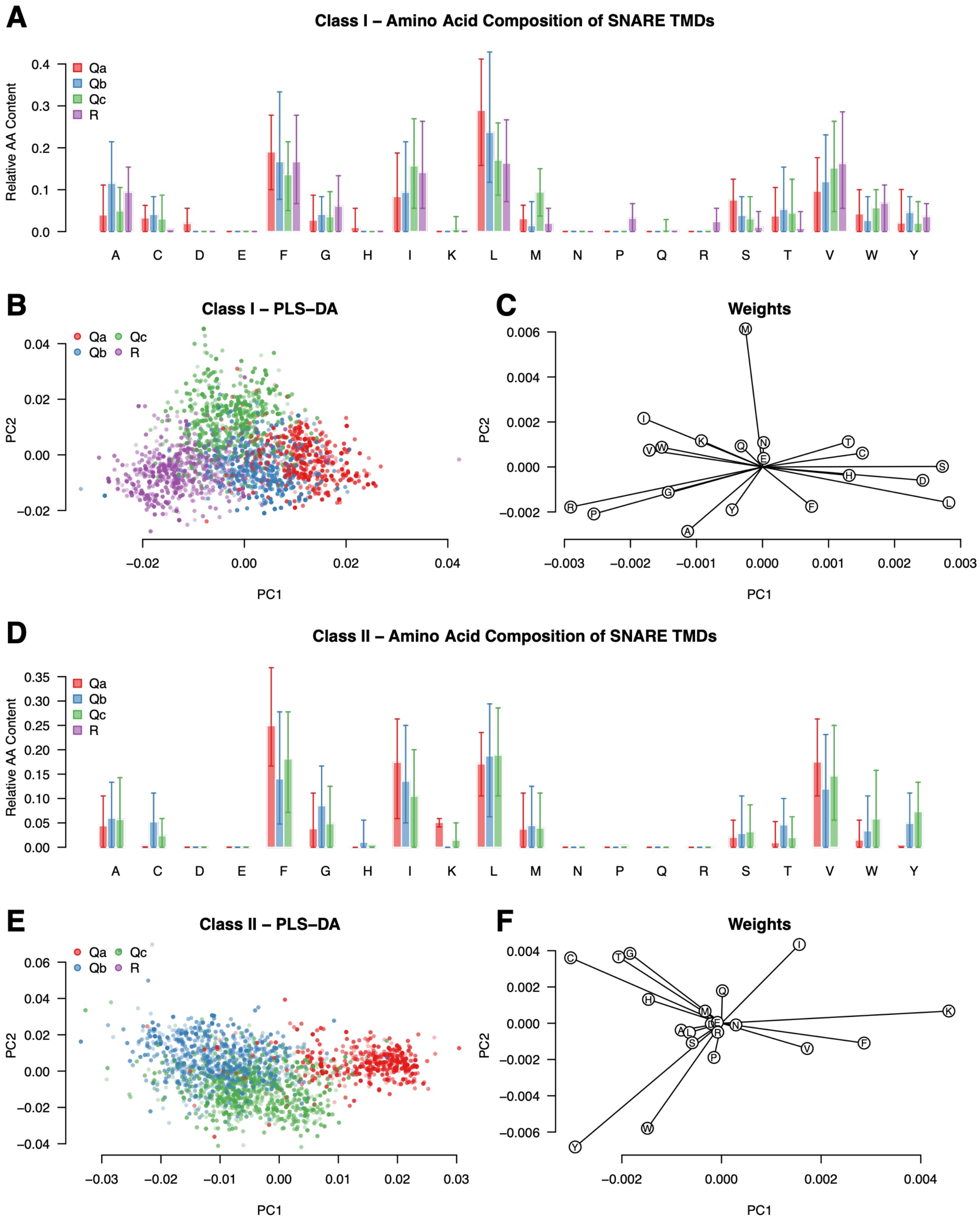

Figure S6. SNARE type-specific variation in TMD amino acid composition of Class I and Class II SNAREs. **(A)** Mean amino acid composition of class I SNARE TMDs. Error bars indicate the range containing 80% of all TMDs. **(B)** Scaled PLS-DA of class I SNARE TMD amino acid composition. Each point represents a single TMD. **(C)** Feature weights contributing to the class I PLS-DA separation. **(D)** Mean amino acid composition of class II SNARE TMDs. Error bars indicate the range containing 80% of all TMDs. **(E)** Scaled PLS-DA of class II SNARE TMD amino acid composition. Each point represents a single TMD. **(F)** Feature weights contributing to the class II PLS-DA separation. Data were resampled by drawing 2,000 sequences per class with replacement.

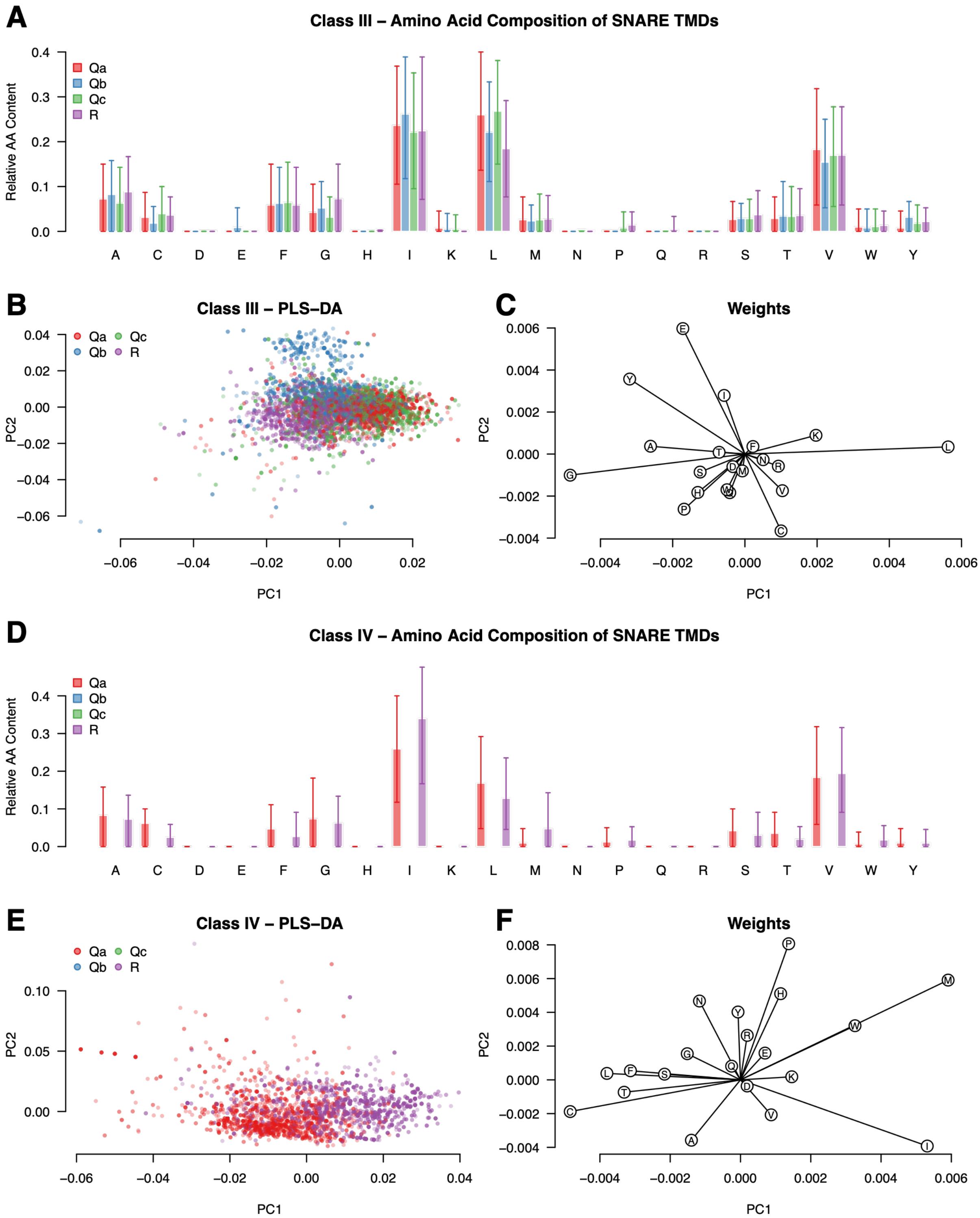

Figure S7. SNARE type-specific variation in TMD amino acid composition of Class III and Class IV SNAREs. **(A)** Mean amino acid composition of class III SNARE TMDs. Error bars indicate the range containing 80% of all TMDs. **(B)** Scaled PLS-DA of class III SNARE TMD amino acid composition. Each point represents a single TMD. **(C)** Feature weights contributing to the class III PLS-DA separation. **(D)** Mean amino acid composition of class IV SNARE TMDs. Error bars indicate the range containing 80% of all TMDs. **(E)** Scaled PLS-DA of class IV SNARE TMD amino acid composition. Each point represents a single TMD. **(F)** Feature weights contributing to the class IV PLS-DA separation. Data were resampled by drawing 2,000 sequences per class with replacement.

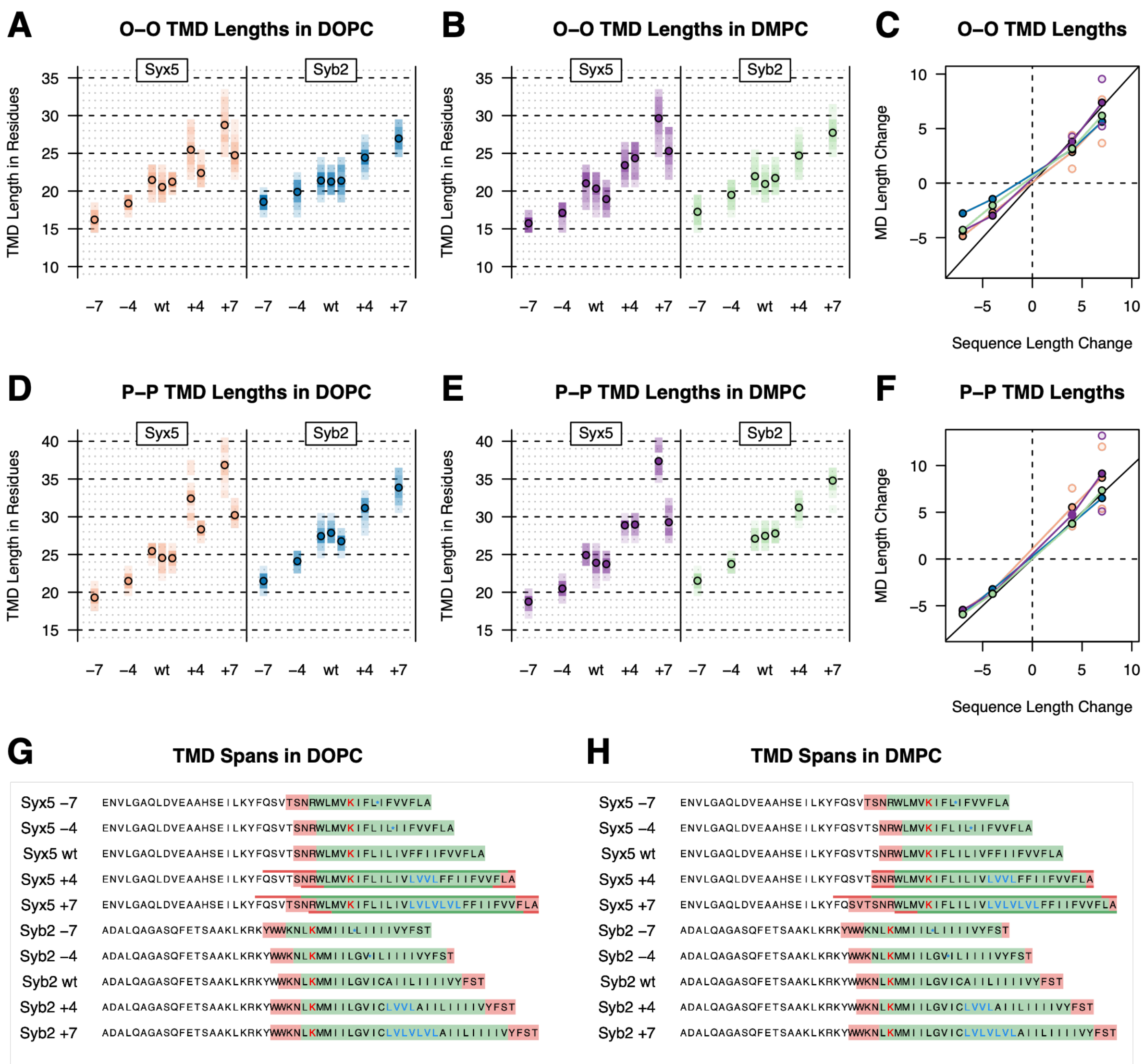

Figure S8. TMD lengths of Syx5 and Syb2 with modified TMDs. TMDs were truncated and elongated by 4 and 7 residues, which roughly correspond to one and two helical turns. Residues were removed from or added to the center of the TMDs. The wt MD simulation data are identical to those used for the comparison between SNAREs and were performed in triplicates. Elongated Syx5 constructs were simulated in duplicates. PPM 2.0 positioned these TMDs with a strong initial tilt, leading to partial insertion of TMD-adjacent residues (similar to Fig. S19; example frames are shown in Fig. S9). To test the influence of the initial positioning in this case, a second MD run was performed using an alternative initial orientation with substantially reduced tilt, positioning the Lys residue in the Syx5 TMD close to the membrane-water interface (Fig. S9). The first trajectories shown for Syx5 +4 and +7 correspond to the PPM 2.0-derived initial orientation, whereas the second ones correspond to the less tilted initial positioning. The two initial states generally produced distinct MD trajectories. The only exception is Syx5 +4 in DMPC, for which the PPM 2.0 trajectory converged to the alternative conformation. **(A)** Frequency heatmap of TMD lengths determined from the number of Cα atoms located within the O–O layers in DOPC. Color intensity reflects the frequency of observing a given TMD length. The average TMD length for each trajectory is indicated by circles. **(B)** Same as in (A) but using DMPC. **(C)** Correlation between sequence modifications and average MD-derived TMD length based on the O–O definition, relative to the corresponding wt TMDs. Colors are the same as in panels A and B. Averages are shown for Syx5 +4 and +7, with individual trajectory averages displayed as separate, lighter-colored data points. Sequence modifications are largely reflected in the MD-derived TMD lengths. The strongest deviation is observed for the −7 truncations, most prominently for Syb2 in DOPC, indicating that additional residues enter the O–O layers due to the strong truncation. Nevertheless, with the exception of Syb2 in DOPC, all TMDs can be shortened by more than four residues, highlighting that the wt Syx5

and Syb2 TMDs are not at the lower limit of what the model membranes can accommodate. **(D)** Same as in (A) but considering the P–P definition. **(E)** Same as in (B) but considering the P–P definition. **(F)** Same as in (C) but considering the P–P definition. Deviations associated with the −7 truncation are less pronounced than with the O–O definition. **(G)** Residues located within the membrane in ≥50% of frames are color-coded in green for the O–O definition and in red for the P–P definition. The most deeply buried charged residues are highlighted in red. Sequence modifications are highlighted in blue, indicating added residues or the sites of residue removal (marked by an asterisk). For Syx5 +4 and +7, averages from both trajectories are shown. Results from the individual trajectories are indicated above the sequence for the PPM 2.0 positioning and below the sequence for the less tilted initial positioning. **(H)** Same as in (G) but in DMPC.

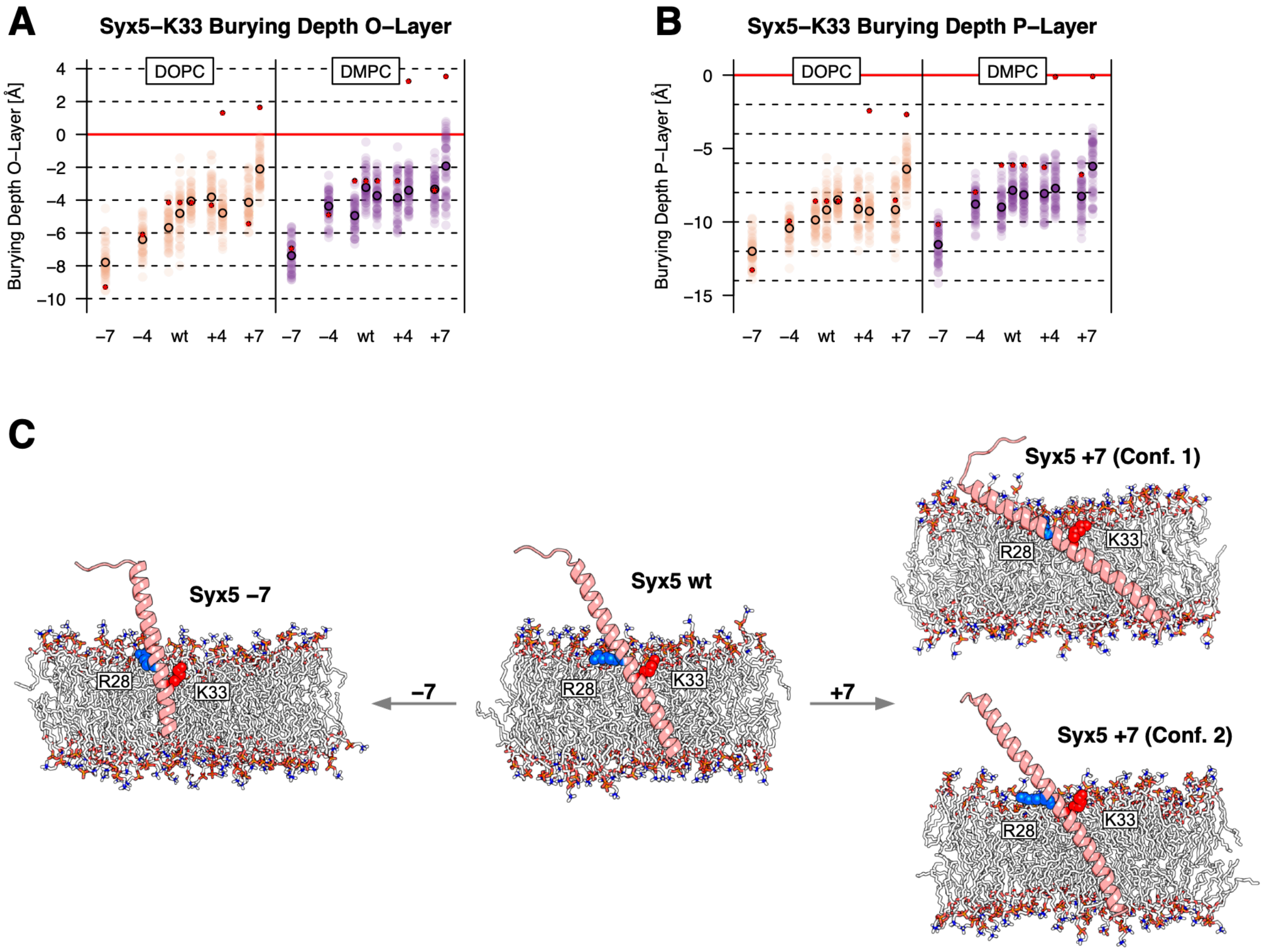

Figure S9. Membrane positioning of K33 in wt and modified Syx5 TMDs. **(A)** Frequency heatmap of K33 Cα burial depth with respect to the O-layer. Color intensity reflects the frequency of observing a given burial depth. The average burial depth for each trajectory is indicated by circles. Red asterisks show the initial burial depth of K33 in the MD setup, highlighting that K33 becomes inserted into the membrane when initially positioned above the O-layer. **(B)** Same as (A), but showing K33 burial depth with respect to the P-layer. **(C)** MD snapshots of Syx5 with wt, truncated (−7), and elongated (+7) TMDs obtained from DOPC simulations to illustrate TMD positioning and the burial of K33. The side chain of K33 is highlighted in red, and R28 is additionally shown in blue, as it also appears to contribute to TMD positioning. For the Syx5 +7 TMD, snapshots from simulations initiated with the two different membrane positions are shown. Conformation 1 was obtained from the initial PPM 2.0 positioning, whereas conformation 2 was obtained from the less tilted initial state.

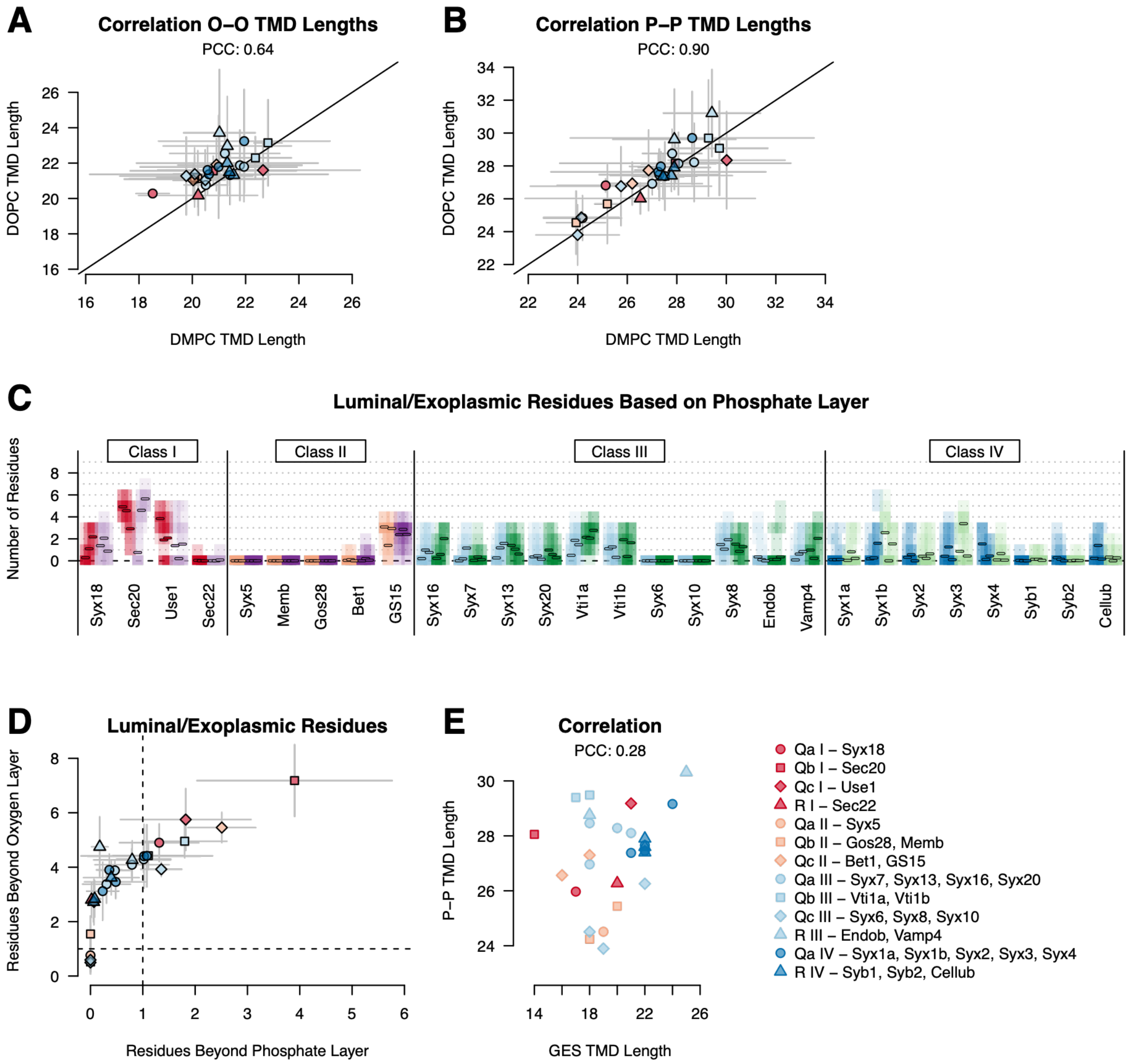

Figure S10. Comparison of TMD lengths from MD simulations and sequence-based predictions. **(A)** Correlation of O–O TMD lengths between DOPC and DMPC membranes. Data points indicate averages, and error bars show the 95% confidence intervals based on the three replicates per membrane. PCC is shown. **(B)** Correlation of P–P TMD lengths between DOPC and DMPC membranes. Averages, 95% confidence intervals, and PCC are shown. **(C)** Frequency heatmap of the number of *C*-terminal residues extending beyond the phosphate layer. Color intensity indicates frequency across trajectories; short bars denote averages per trajectory. **(D)** Average number of *C*-terminal residues outside the membrane using phosphate and oxygen layers considering both membrane types. Error bars indicate the 95% confidence intervals. Dashed lines correspond to the length of a single residue. **(E)** Correlation between MD-derived P–P TMD lengths and sequence-based lengths obtained using the GES hydrophobicity scale.

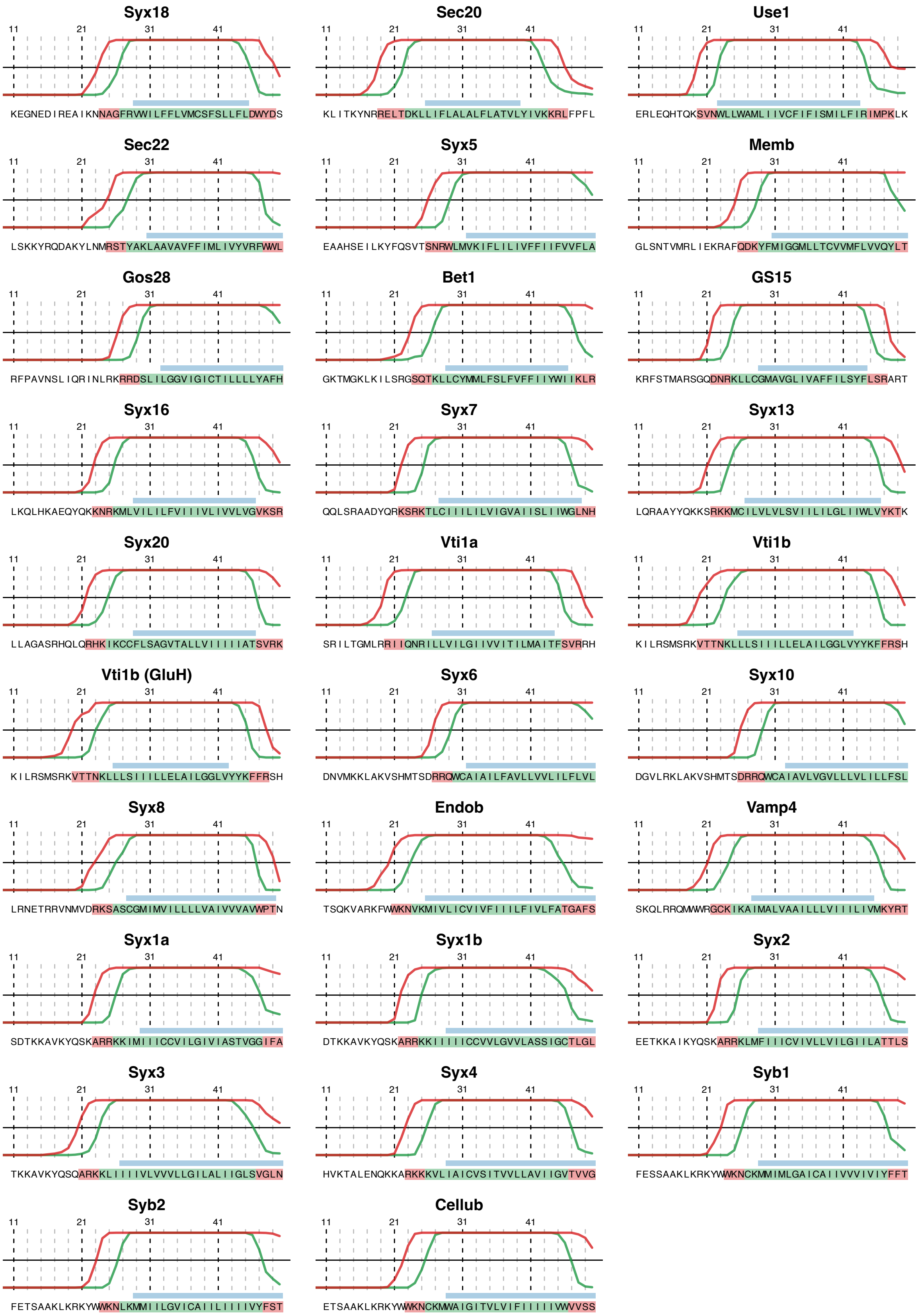

Figure S11. TMDs determined by MD simulations and the GES scale. The *C*-terminal 40 residues of each SNARE protein are shown. Curves indicate the fraction of frames in which each residue resides within the membrane across all trajectories in DOPC and DMPC bilayers, using the O–O definition (green) and P–P definition (red). Residues located within the membrane in ≥50% of frames are color-coded in the sequence accordingly (green for O–O, red for P–P). The blue bar highlights residues assigned to the TMD by the GES hydrophobicity scale. Vti1b is shown for both the charged and protonated (GluH) states.

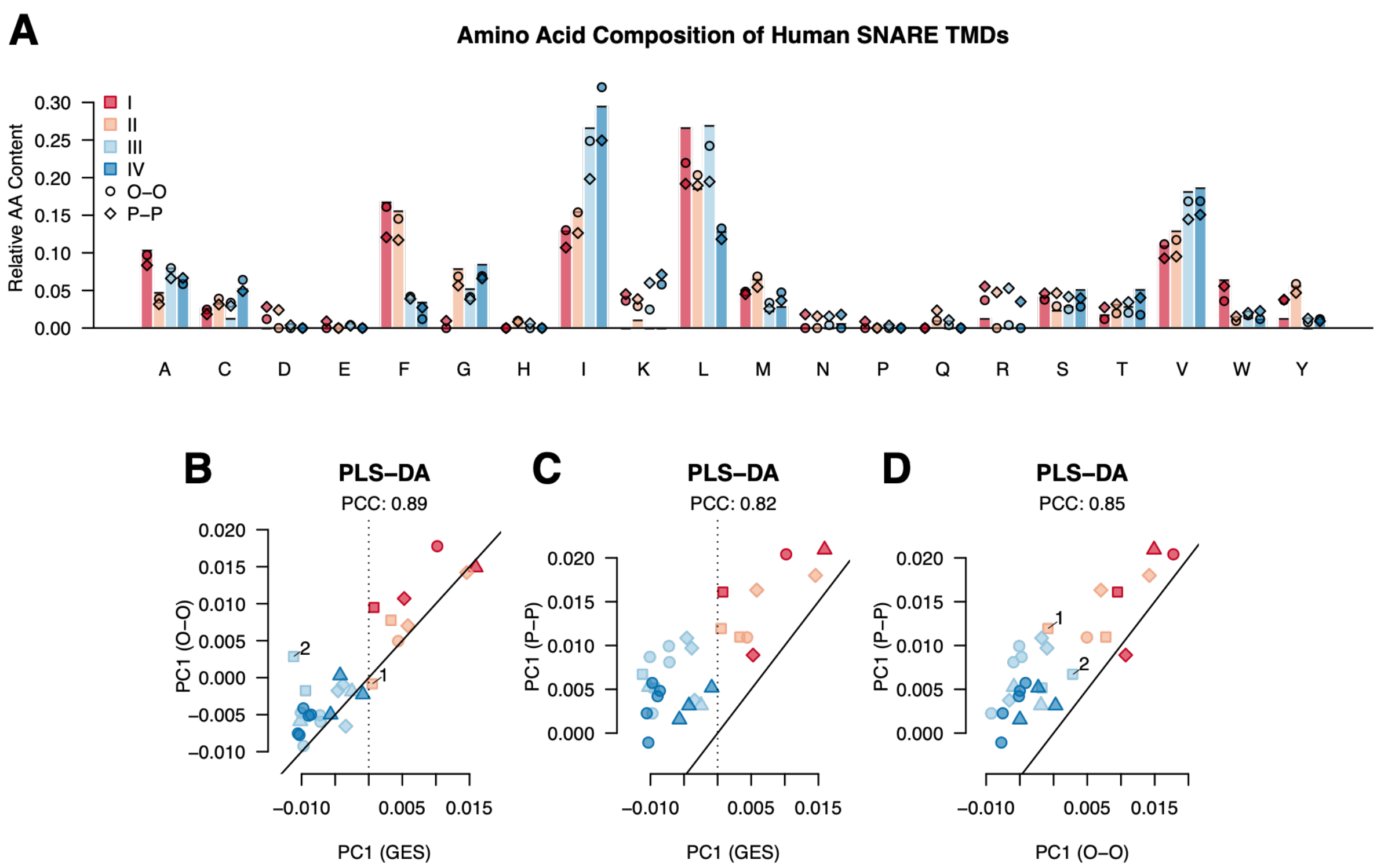

Figure S12. Amino acid composition of GES- and MD-defined SNARE TMDs. **(A)** Mean amino acid composition of human SNARE TMDs when determined using either the GES hydrophobicity scale and MD simulations. **(B)** Correlation of PLS-DA PC1 scores between TMDs defined by the O–O definition and the GES scale. PC1 scores were calculated using feature weights from the PLS-DA of the complete SNARE TMD dataset. Gos28 (1) and Vti1b (2) are highlighted. The dotted line highlights the separation between classes I/II and III/IV. **(C)** Correlation of PLS-DA PC1 scores between TMDs defined by the P–P definition and the GES scale. The dotted line highlights the separation between classes I/II and III/IV. **(D)** Correlation of PLS-DA PC1 scores between TMDs defined by the P–P and O–O definitions. Gos28 (1) and Vti1b (2) are highlighted.

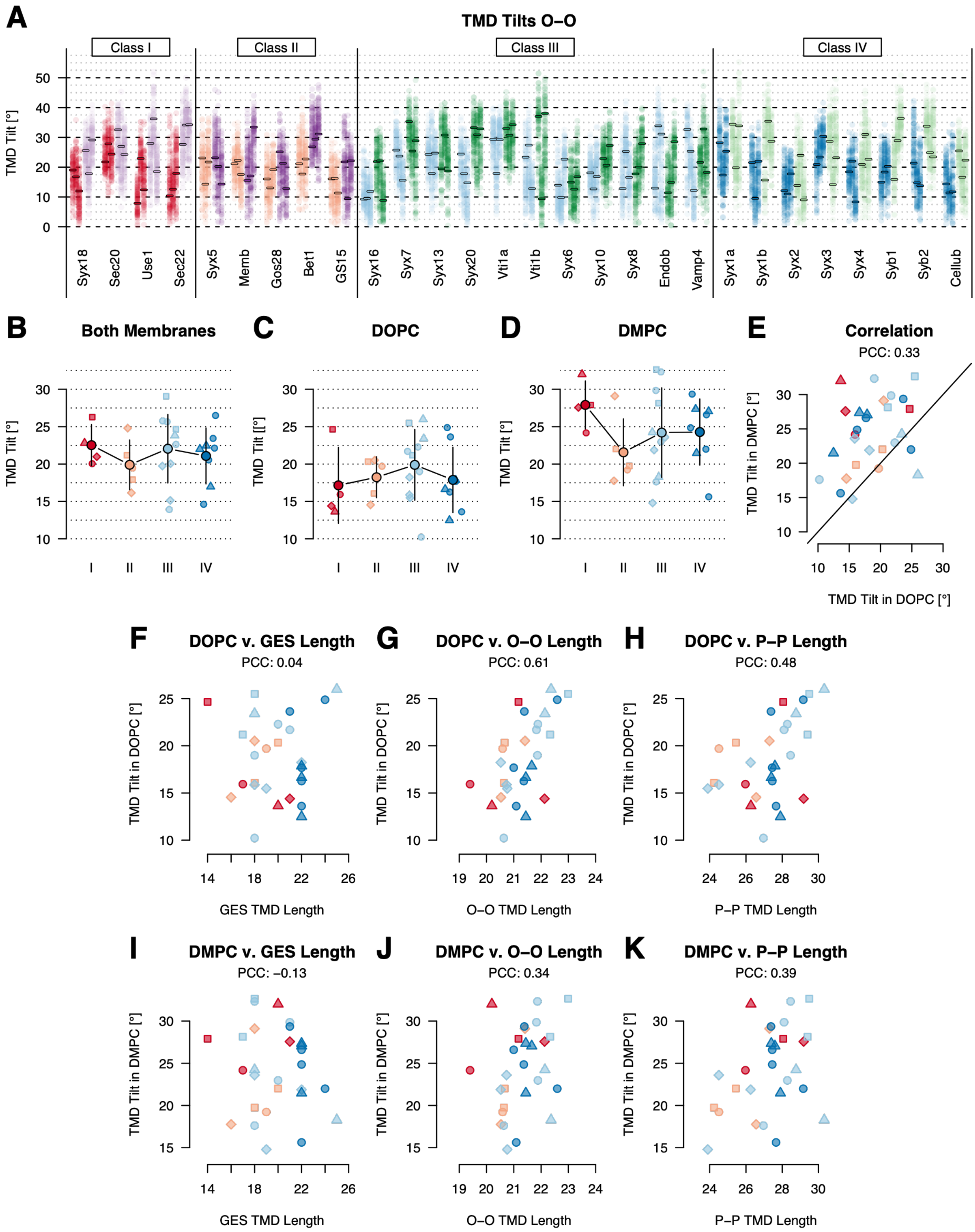

Figure S13. TMD tilts of human SNAREs determined from MD simulations using residues within the O–O layer. In the data shown here, the Glu residue in the Vti1b TMD is protonated and thus neutral. Helix tilt angles were calculated based on a smoothed representation of the helix axis: Midpoints between Cα atoms of neighboring residues (i−1 and i+1) were first determined to define a centerline. These coordinates were subsequently smoothed using a three-residue rolling average to reduce local structural fluctuations. The resulting coordinates were transformed using PCA with centering and without scaling, and the resulting PC1 was taken as the helix axis. The tilt angle was defined as the angle between PC1 and the membrane normal (z-axis). **(A)** Frequency heatmap of TMD tilts from MD simulations. For each SNARE, results obtained in DOPC are shown on the left and those in DMPC on the right using complementary colors. Color intensity reflects the frequency of observing a given tilt

angle. The average tilt for each trajectory is indicated by a short bar. **(B)** TMD tilts of human SNAREs obtained from MD simulations in both membrane types. Individual protein values are shown in the background, while class averages are displayed in the foreground; bars indicate standard deviations. **(C)** Same as in (B), but showing TMD tilts derived from simulations in DOPC only. **(D)** Same as in (B), but showing TMD tilts derived from simulations in DMPC only. **(E)** Correlation between TMD tilts obtained from MD simulations in DOPC and DMPC. **(F–H)** Correlation between TMD tilts from MD simulations in DOPC and (F) GES-derived TMD lengths, (G) O–O TMD lengths, and (H) P–P TMD lengths. **(I–K)** Correlation between TMD tilts from MD simulations in DMPC and (I) GES-derived TMD lengths, (J) O–O TMD lengths, and (K) P–P TMD lengths.

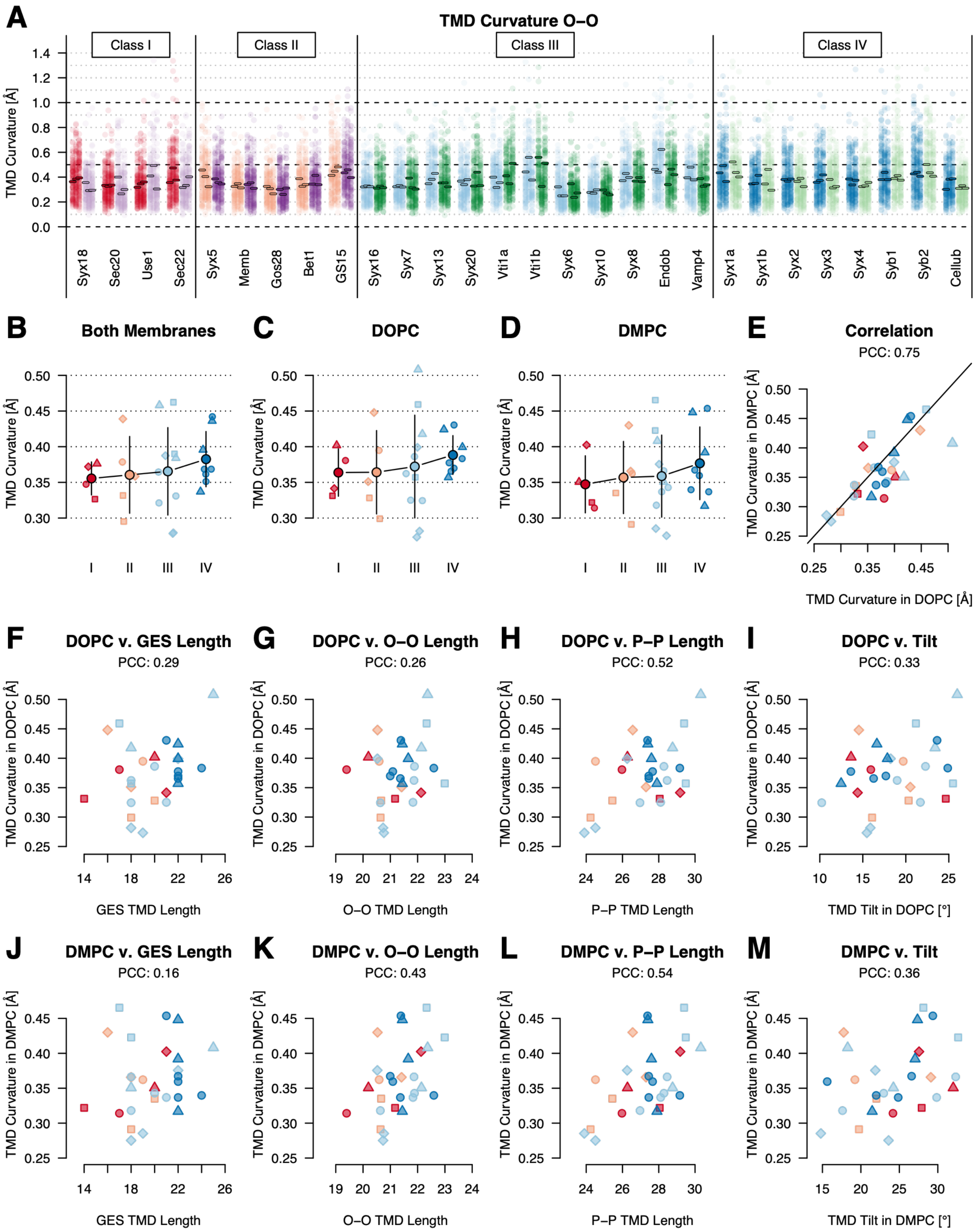

Figure S14. TMD curvatures of human SNAREs determined from MD simulations using residues within the O–O layer. In the data shown here, the Glu residue in the Vti1b TMD is protonated and thus neutral. Helix curvature was quantified as the average deviation of the smoothed centerline from the principal helix axis (see Fig. S13). Specifically, the perpendicular displacement of each point from the principal axis was calculated as:

$$Curvature = \frac{1}{n}\sum_{i=1}^{n}\sqrt{PC2_i^2 + PC3_i^2}$$

This metric reflects the extent to which the helix deviates from an ideal straight axis, with larger values indicating increased curvature. **(A)** Frequency heatmap of TMD curvatures from MD simulations. For each SNARE, results

obtained in DOPC are shown on the left and those in DMPC on the right using complementary colors. Color intensity reflects the frequency of observing a given curvature. The average curvature for each trajectory is indicated by a short bar. **(B)** TMD curvatures of human SNAREs obtained from MD simulations in both membrane types. Individual protein values are shown in the background, while class averages are displayed in the foreground; bars indicate standard deviations. **(C)** Same as in (B), but showing TMD curvatures derived from simulations in DOPC only. **(D)** Same as in (B), but showing TMD curvatures derived from simulations in DMPC only. **(E)** Correlation between TMD curvatures obtained from MD simulations in DOPC and DMPC. **(F–I)** Correlation between TMD curvatures from MD simulations in DOPC and (F) GES-derived TMD lengths, (G) O–O TMD lengths, (H) P–P TMD lengths, and (I) TMD tilt. **(J–M)** Correlation between TMD curvatures from MD simulations in DMPC and (J) GES-derived TMD lengths, (K) O–O TMD lengths, (L) P–P TMD lengths, and (M) TMD tilt.

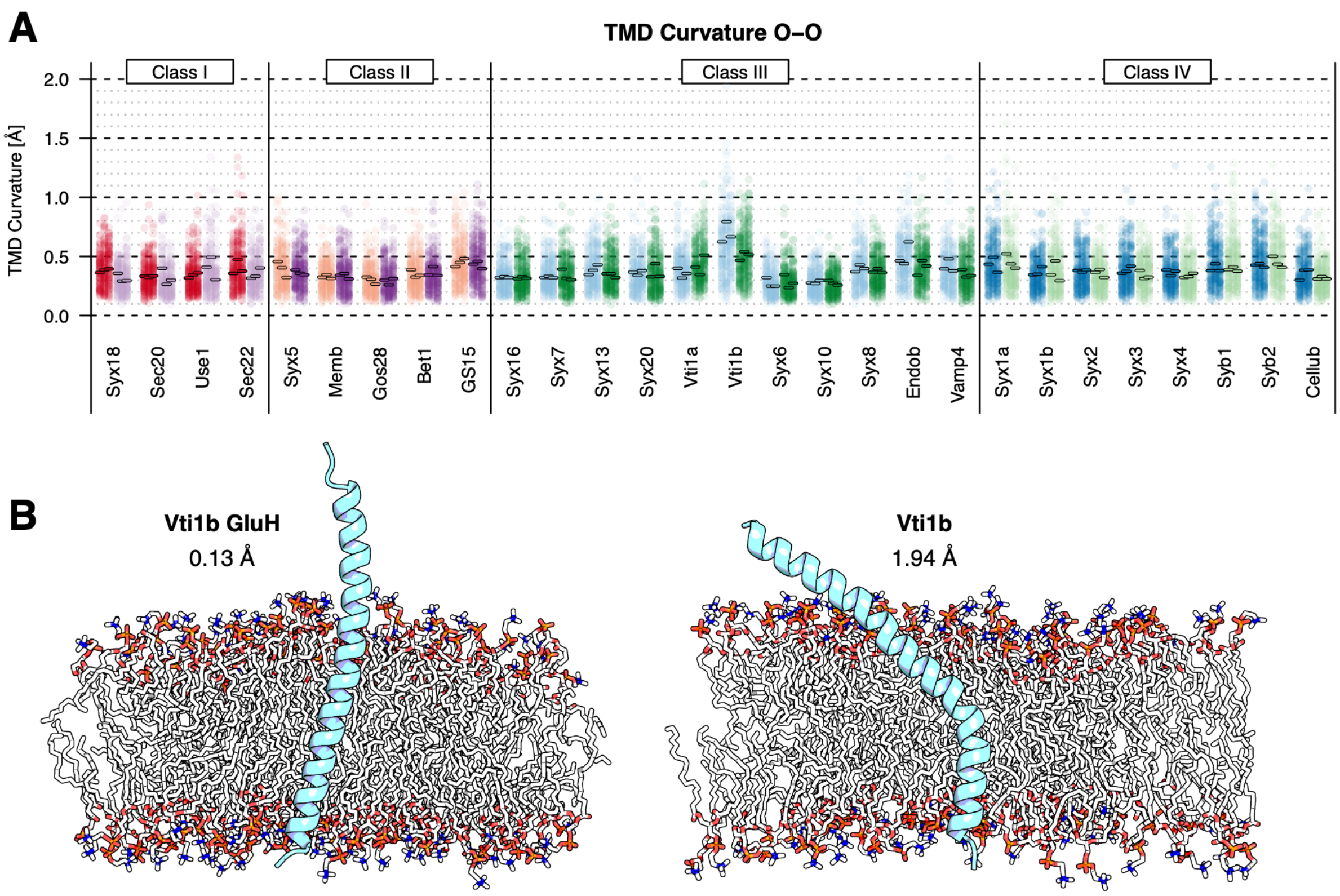

Figure S15. TMD curvatures of human SNAREs determined from MD simulations using residues within the O–O layer. In the data shown here, the Glu residue in the Vti1b TMD is deprotonated and therefore charged. Helix curvature was quantified as described in Fig. S14. **(A)** Frequency heatmap of TMD curvatures from MD simulations. For each SNARE, results obtained in DOPC are shown on the left and those in DMPC on the right using complementary colors. Color intensity reflects the frequency of observing a given curvature. The average curvature for each trajectory is indicated by a short bar. **(B)** Representative snapshots of Vti1b in DOPC with Glu in its neutral (Vti1b GluH) and charged (Vti1b) states, illustrating TMD curvature. Curvature values are given in Å.

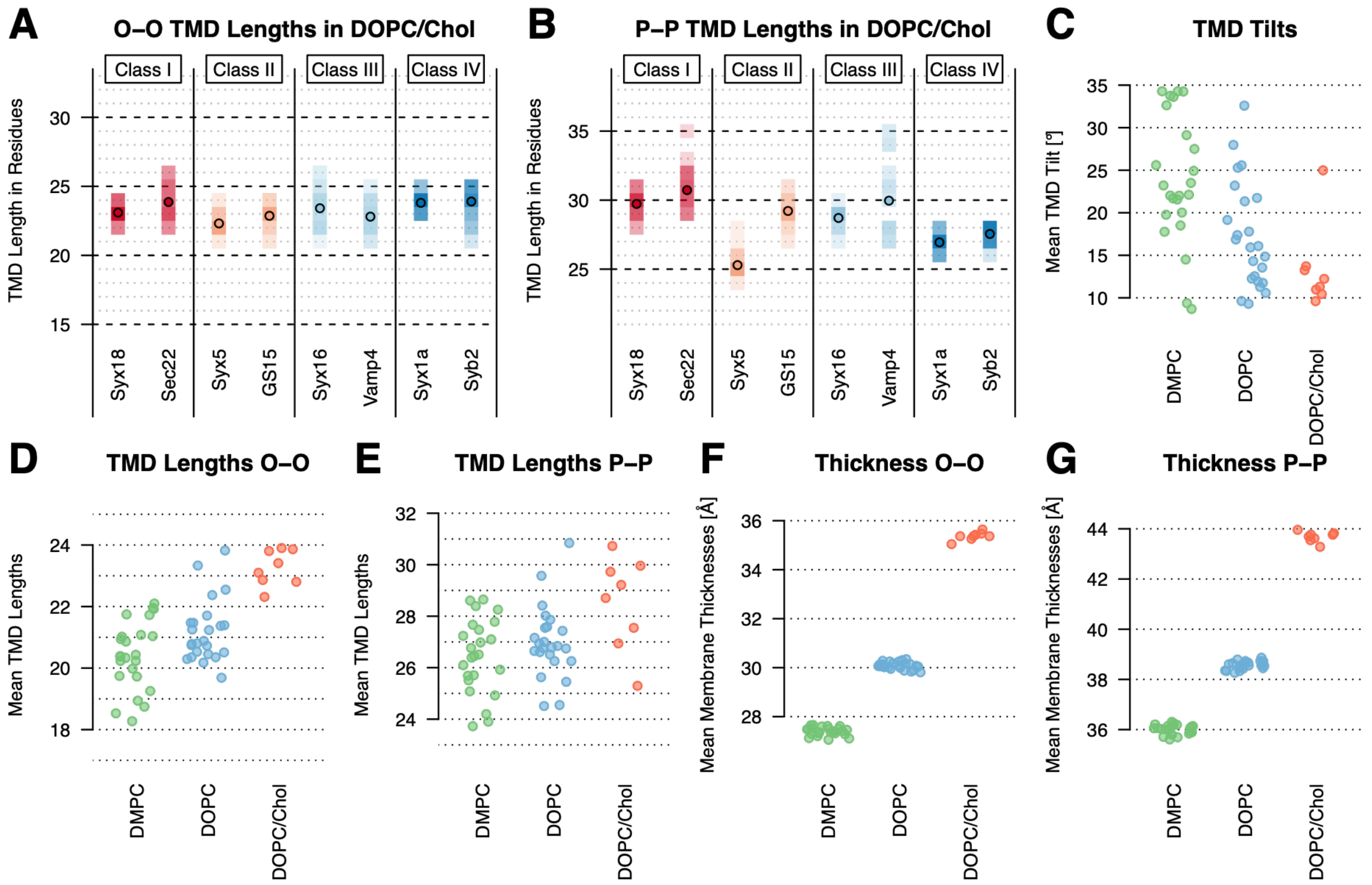

Figure S16. TMD lengths of a subset of human SNAREs determined from MD simulations in a mixed bilayer containing 60% DOPC and 40% cholesterol. Each leaflet contains 60 DOPC and 40 cholesterol molecules. For the definition of O–O and P–P boundaries, only the six DOPC molecules closest to the TMD helix in each leaflet were considered to account for the reduced DOPC density compared to pure DOPC bilayers. All other calculations were performed as described for DOPC and DMPC bilayers. **(A)** Frequency heatmap of TMD lengths determined from the number of Cα atoms located within the O–O layers. Color intensity reflects the frequency of observing a given TMD length. The average TMD length for each trajectory is indicated by a circle. **(B)** Same as in (A), but with TMD lengths defined using the P–P boundaries. **(C)** TMD tilts of the SNARE subset analyzed in cholesterol-containing membranes. Each data point represents the trajectory average. DOPC and DMPC simulations were performed in triplicate, resulting in threefold more data points. **(D)** TMD lengths based on the O–O definition. Trajectory averages are shown. **(E)** TMD lengths based on the P–P definition. Trajectory averages are shown. **(F)** Membrane thickness based on the O–O definition. Trajectory averages are shown. **(G)** Membrane thickness based on the P–P definition. Trajectory averages are shown.

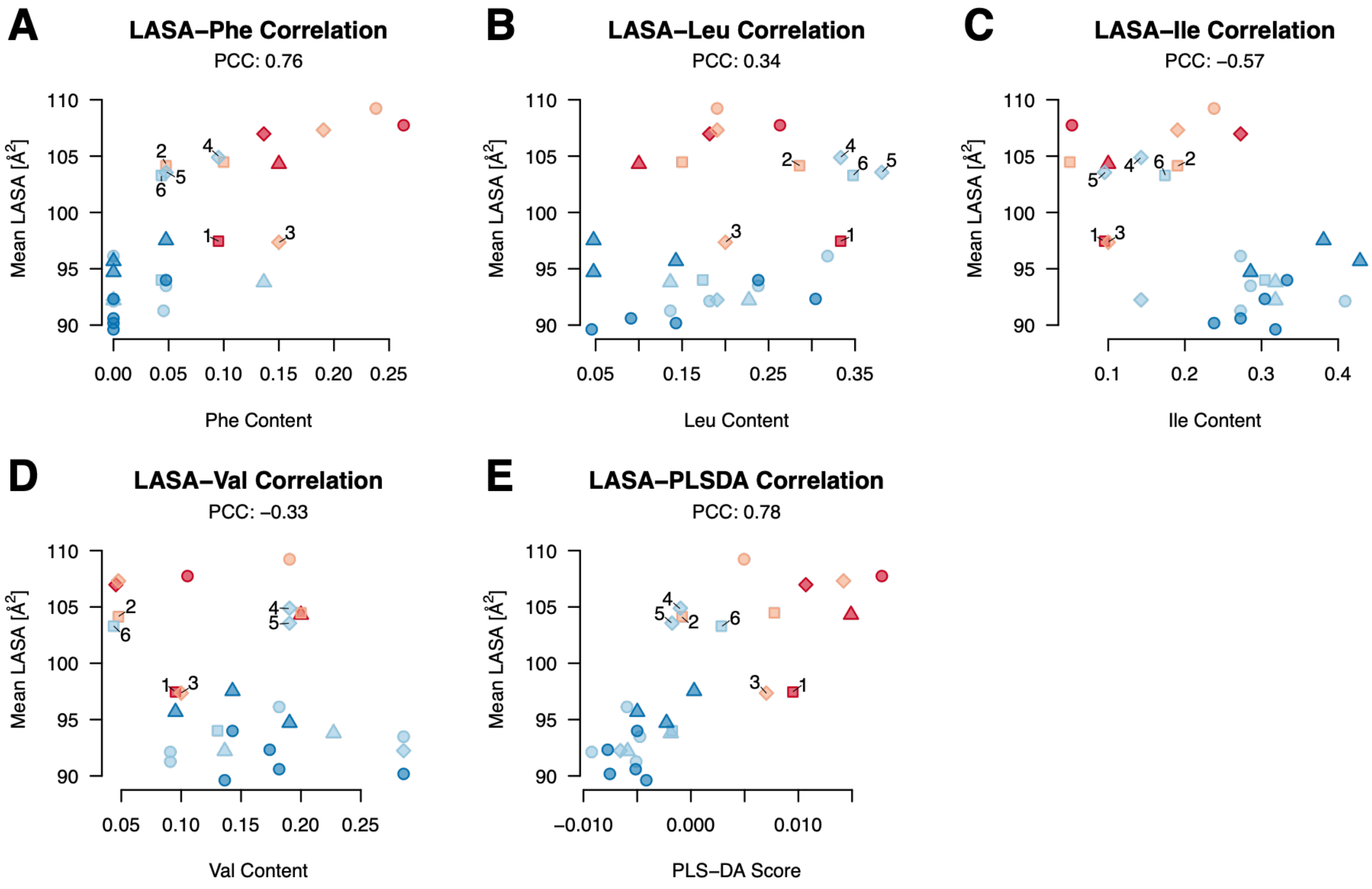

Figure S17. Correlations between LASA and amino acid composition in human SNARE TMDs based on the O–O definition: **(A)** LASA versus Phe content. **(B)** LASA versus Leu content. **(C)** LASA versus Ile content. **(D)** LASA versus Val content. **(E)** LASA versus PLS-DA scores. PCCs are indicated at the top of each plot. Highlighted SNAREs: Sec20 (1), Gos28 (2), GS15 (3), Syx6 (4), Syx10 (5), Vti1b (6).

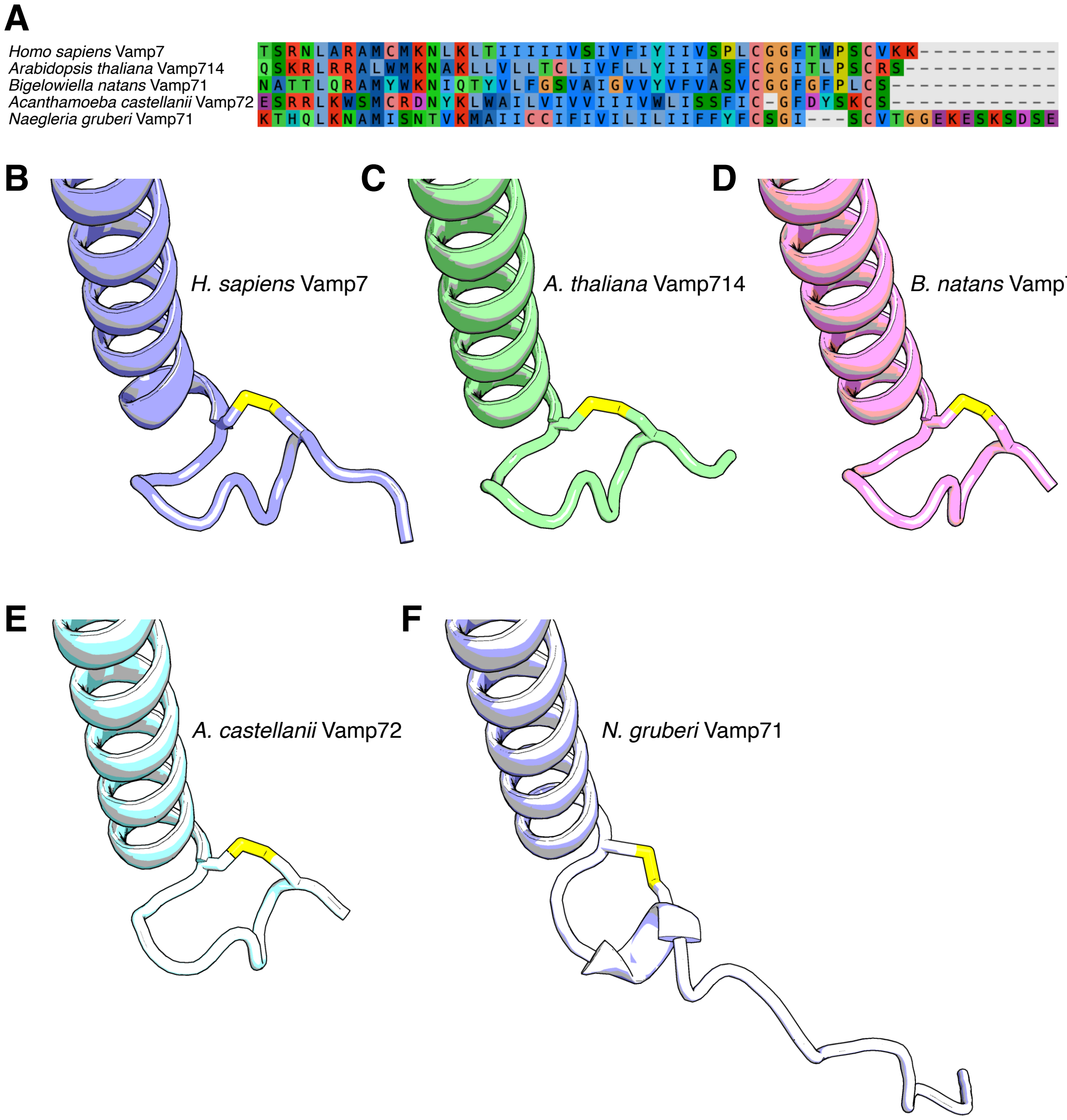

Figure S18. Conservation of the *C*-terminal region of Vamp7 across eukaryotes. **(A)** Multiple sequence alignment of the *C*-terminal regions of Vamp7 orthologs across Eukaryota. AF2 structural models are shown for **(B)** *Homo sapiens* (Obazoa) Vamp7, **(C)** *Arabidopsis thaliana* (Archaeplastida) Vamp714, **(D)** *Bigelowiella natans* (SAR) Vamp71, **(E)** *Acanthamoeba castellanii* (Amoebozoa) Vamp72, and **(F)** *Naegleria gruberi* (Discoba) Vamp71. Sulfur atoms forming the potentially conserved disulfide bond are highlighted in yellow.

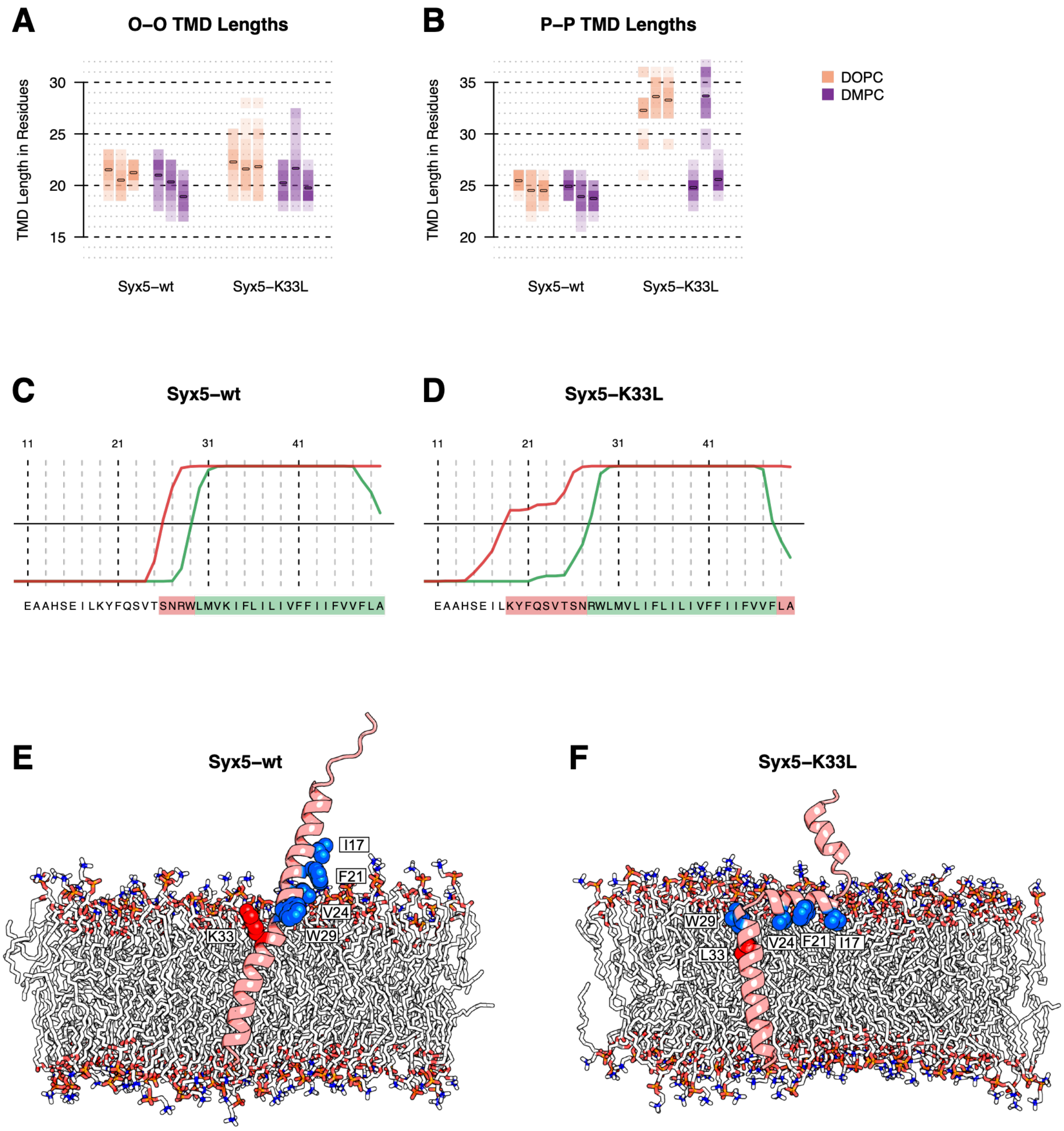

Figure S19. Effect of the K33L substitution on Syx5 TMD length and membrane insertion. **(A)** Frequency heatmap of TMD lengths based on the P–P definition for wt- and K33L-Syx5 trajectories. Color intensity indicates how frequently a given TMD length was observed. Small bars indicate the mean for each trajectory. **(B)** Same as (A), but using the O–O definition. Most K33L-Syx5 trajectories display an increase in TMD length due to more residues being inserted on the cytosolic side of the membrane. **(C, D)** TMDs determined by MD simulations. The *C*-terminal 40 residues of wt-Syx5 (C) and K33L-Syx5 (D) are shown. Curves indicate the fraction of frames in which each residue resides within the membrane across all trajectories, using the O–O (green) and P–P definition (red). Residues located within the membrane in ≥50% of frames are color-coded in the sequence accordingly (green for O–O, red for P–P). **(E, F)** MD snapshots of wt-Syx5 (E) and K33L-Syx5 (F) obtained from simulations with DOPC to illustrate the sampled conformational states. The interfacial Trp residue and the hydrophobic residues of the amphipathic helix are highlighted in blue. The Lys residue in the wt TMD and its Leu substitution are shown in red. The K33L substitution promotes deeper insertion of the TMD into the membrane and induces a pronounced kink within the helix, resulting in partial insertion of the TMD-adjacent region through the hydrophobic face of the amphipathic helix. These findings suggest that K33 contributes to proper positioning of the TMD within the membrane.

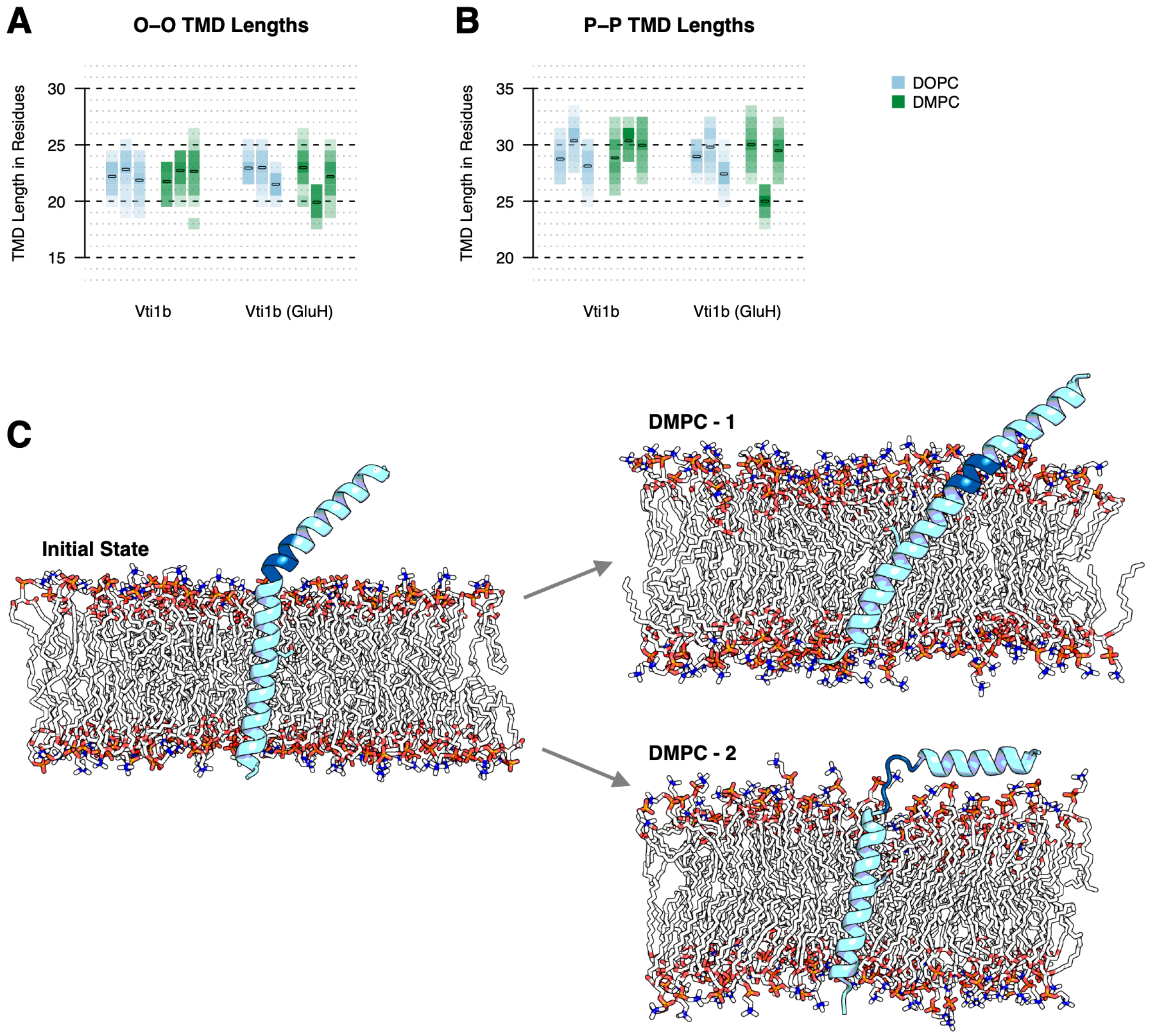

Figure S20. Influence of Glu protonation on Vti1b TMD length. **(A)** Frequency heatmap of TMD lengths based on the P–P definition for all Vti1b trajectories with Glu in its charged and in its neutral (GluH) state. Color intensity indicates how frequently a given TMD length was observed. Small bars indicate the mean for each trajectory. Differences between the two Glu protonation states were not statistically significant (two-tailed t-test, $p = 0.65$). **(B)** Same as (A), but using the O–O definition ($p = 0.31$). One Vti1b (GluH) trajectory in DMPC displays a markedly shorter TMD due to formation of a loop between the TMD and the *N*-terminus. **(C)** MD snapshots of Vti1b obtained from DMPC simulations illustrating the sampled conformation states. The loop observed in the second DMPC trajectory (residues 18–RKVTTN–23) is highlighted in dark blue. Similar rearrangements of the *N*-terminal region occurred in other SNARE simulations, such as the first trajectory of Syx1b in DMPC. Looped or kinked states have also been reported previously.[1]

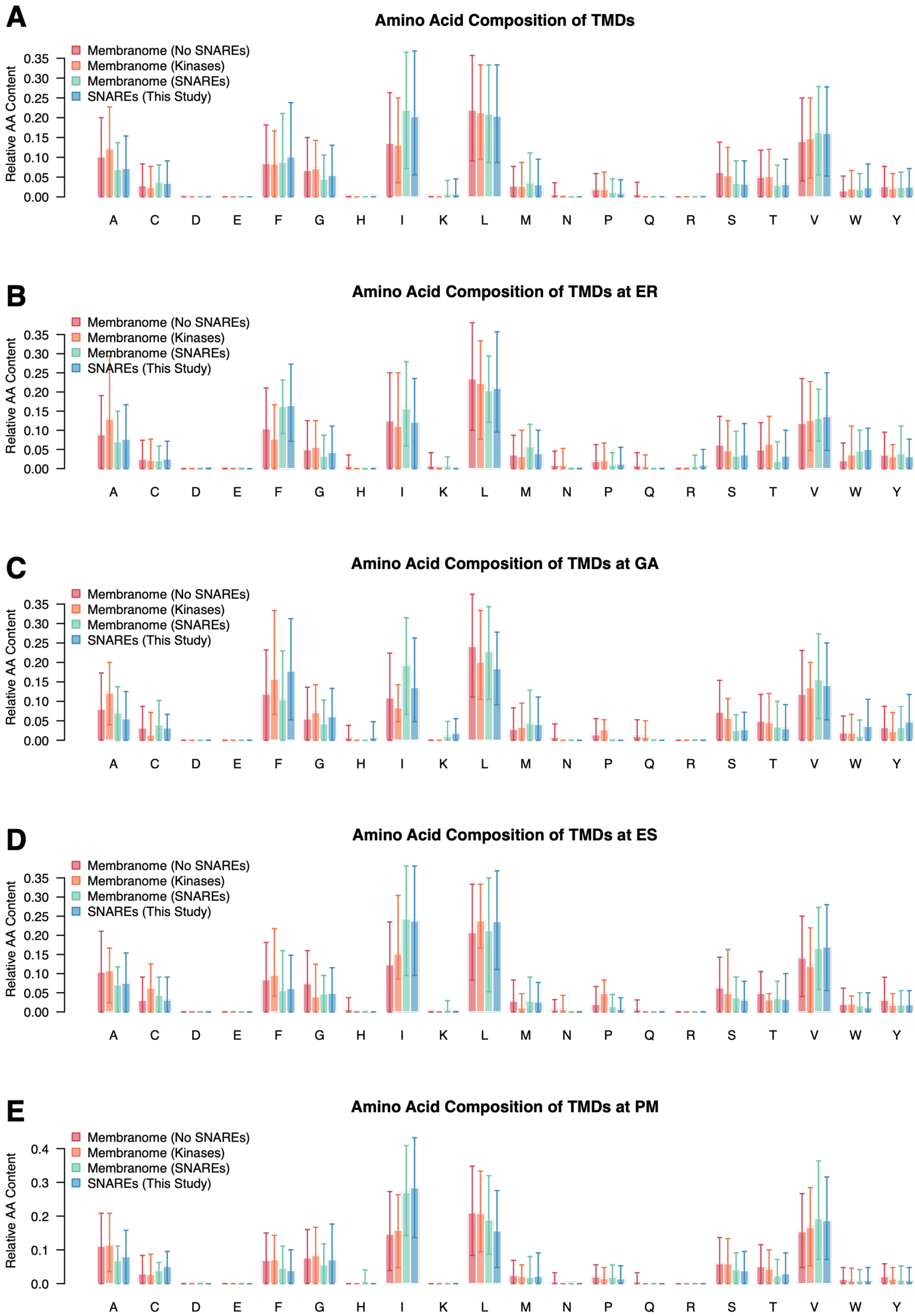

Figure S21. Comparison of amino acid composition between SNARE and non-SNARE TMDs. MembranomeX data (https://biomembhub.org/membranome/) were retrieved on 11 May 2026.[2] To ensure comparability, TMD boundaries were redefined using the GES hydrophobicity scale, as applied to SNARE proteins in this study. MembranomeX TMDs were assigned to the same classes used for SNAREs based on annotated subcellular

localization: class I (ER, 1958 TMDs), class II (GA, 1232 TMDs), class III (ES; including vesicles, endosomes, lysosomes, melanosomes, peroxisomes, and vacuoles; 353 TMDs), and class IV (PM; 5053 TMDs). SNARE proteins were extracted from the MembranomeX data based on annotations. As a non-SNARE example, kinase TMDs were extracted from the MembranomeX data based on annotations. Due to the low number of kinases outside the PM, TMDs were resampled with replacement to generate datasets of 5000 sequences each for ER/GA and ES/PM proteins, respectively. **(A)** Mean amino acid composition of TMDs. Error bars indicate the central 80% range of the distribution. **(B–E)** Mean amino acid composition of TMDs by subcellular compartment: ER (B), GA (C), ES (D), and PM (E). Error bars indicate the central 80% range of the distribution.

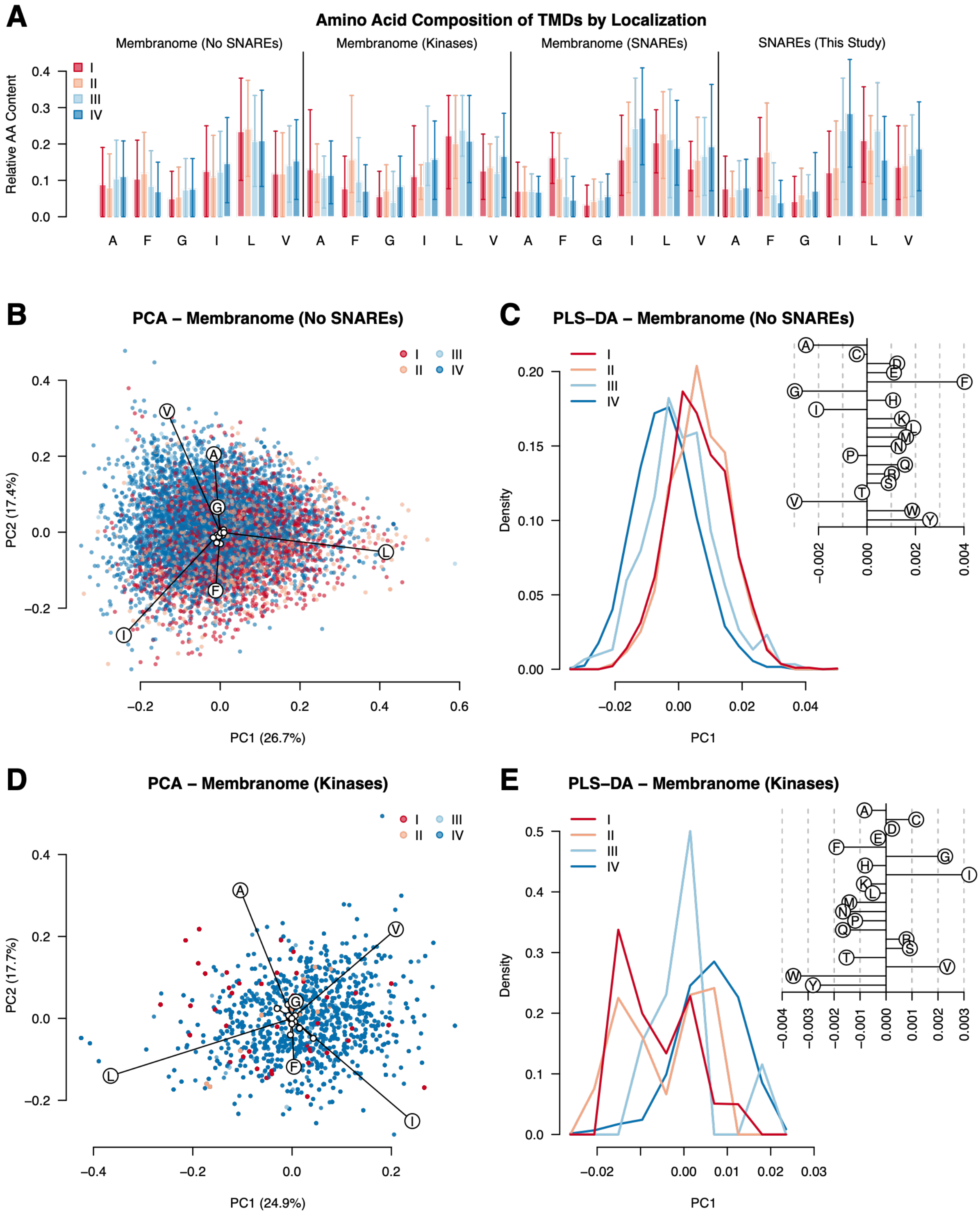

Figure S22. Comparison of amino acid composition between SNARE and non-SNARE TMDs. For MembranomeX data preparation, see Fig. S21 legend. **(A)** Mean amino acid composition of TMDs by protein group and subcellular localization. Error bars indicate the central 80% range of the distribution.**(B)** PCA of amino acid composition for MembranomeX non-SNARE TMDs, with corresponding feature loadings. Each point represents a single TMD sequence. **(C)** Histogram of PLS-DA results separating classes I/II and III/IV of MembranomeX non-SNARE TMDs; feature weights are shown in the upper-right inset. **(D)** PCA of amino acid composition for MembranomeX kinase TMDs, with corresponding feature loadings. Each point represents a single TMD sequence. **(E)** Histogram of PLS-DA results separating classes I/II and III/IV of MembranomeX kinase TMDs; feature weights are shown in the upper-right inset.

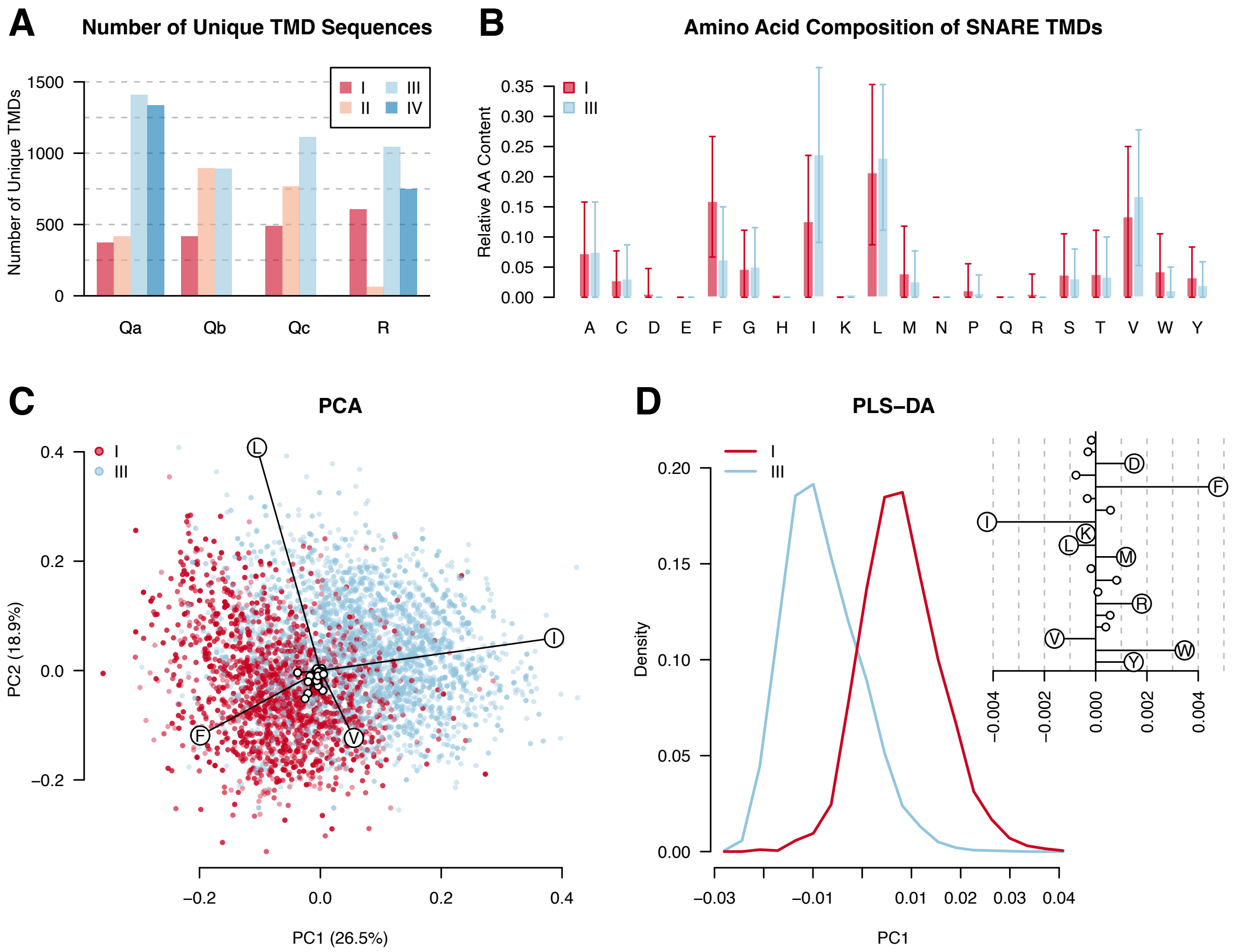

Figure S23. Subsampling analysis of SNARE TMD amino acid composition. **(A)** Number of unique SNARE TMD sequences grouped by type (Qa, Qb, Qc, R) and class (I–IV). Only class I and III contain all four SNARE types and were therefore used for this analysis. For each type-class combination (e.g., Qa II), 1,000 TMDs were randomly sampled with replacement. **(B)** Mean amino acid composition of class I and class III SNARE TMDs. TMDs were defined using the GES hydrophobicity scale. Error bars indicate the range containing 80% of all TMDs. **(C)** PCA of SNARE TMD amino acid composition with corresponding relative loadings. Each point represents one TMD sequence. **(D)** Histogram of PLS-DA results separating classes I/II and III/IV, with feature weights shown in the upper-right panel.

Alignment S1. *C*-terminal sequences of human SNAREs used for MD simulations in this study.

```
>01_Syx18
QLVVGATENIKEGNEDIREAIKNNAGFRVWILFFLVMCSFSLLFLDWYDS
>02_Sec20
SMSGTIQLGRKLITKYNRRELTDKLLIFLALALFLATVLYIVKKRLFPFL
>03_Use1
QNLEKLKTESERLEQHTQKSVNWLLWAMLIIVCFIFISMILFIRIMPKLK
>04_Sec22
LDSKANNLSSLSKKYRQDAKYLNMRSTYAKLAAVAVFFIMLIVYVRFWWL
>05_Syx5
ENVLGAQLDVEAAHSEILKYFQSVTSNRWLMVKIFLILIVFFIIFVVFLA
>06_Memb
KKILDIANMLGLSNTVMRLIEKRAFQDKYFMIGGMLLTCVVMFLVVQYLT
>07_Gos28
IHSKMNTLANRFPAVNSLIQRINLRKRRDSLILGGVIGICTILLLLYAFH
>08_Bet1
SQFDSTTGFLGKTMGKLKILSRGSQTKLLCYMMLFSLFVFFIIYWIIKLR
>09_GS15
SMTSLLTGSVKRFSTMARSGQDNRKLLCGMAVGLIVAFFILSYFLSRART
>10_Syx16
EQSCIKTEDGLKQLHKAEQYQKKNRKMLVILILFVIIIVLIVVLVGVKSR
>11_Syx7
NAEVHVQQANQQLSRAADYQRKSRKTLCIIILILVIGVAIISLIIWGLNH
>12_Syx13
EVHVERATEQLQRAAYYQKKSRKKMCILVLVLSVIILILGLIIWLVYKTK
>13_Syx20
ASSHAEAARQLLAGASRHQLQRHKIKCCFLSAGVTALLVIIIIIATSVRK
>14_Vti1a
RETDANLGKSSRILTGMLRRIIQNRILLVILGIIVVITILMAITFSVRRH
>15_Vti1b
NTSENLSKSRKILRSMSRKVTTNKLLLSIIILLELAILGGLVYYKFFRSH
>16_Syx6
HELESTQSRLDNVMKKLAKVSHMTSDRRQWCAIAILFAVLLVVLILFLVL
>17_Syx10
QEMDHTQSRMDGVLRKLAKVSHMTSDRRQWCAIAVLVGVLLLVLILLFSL
>18_Syx8
ANLVENTDEKLRNETRRVNMVDRKSASCGMIMVILLLLVAIVVVAVWPTN
>19_Endob
LEATSEHFKTTSQKVARKFWWKNVKMIVLICVIVFIIILFIVLFATGAFS
>20_Vamp4
SDNATAFSNRSKQLRRQMWWRGCKIKAIMALVAAILLLVIIILIVMKYRT
>21_Vamp7
FKTTSRNLARAMCMKNLKLTIIIIIVSIVFIYIIVSPLCGGFTWPSCVKK
>22_Syx1a
HAVDYVERAVSDTKKAVKYQSKARRKKIMIIICCVILGIVIASTVGGIFA
>23_Syx1b
SVDYVERAVSDTKKAVKYQSKARRKKIIIIICCVVLGVVLASSIGCTLGL
>24_Syx2
NATDYVEHAKEETKKAIKYQSKARRKLMFIIICVIVLLVILGIILATTLS
>25_Syx3
VDHVEKARDETKKAVKYQSQARKKLIIIIVLVVVLLGILALIIGLSVGLN
>26_Syx4
SADYVERGQEHVKTALENQKKARKKKVLIAICVSITVVLLAVIIGVTVVG
>27_Syb1
ADALQAGASQFESSAAKLKRKYWWKNCKMMIMLGAICAIIVVVIVIYFFT
>28_Syb2
ADALQAGASQFETSAAKLKRKYWWKNLKMMIILGVICAIILIIIIVYFST
>29_Cellub
DALQAGASQFETSAAKLKRKYWWKNCKMWAIGITVLVIFIIIIIVWVVSS
>30_Myob
MSSTFNKTTQNLAQKKCWENIRYRICVGLVVVGVLLIIILIVLLVVFLPQSSDSSS
```

References

1. Lindau M, Hall BA, Chetwynd A, Beckstein O, Sansom MS. Coarse-grain simulations reveal movement of the synaptobrevin C-terminus in response to piconewton forces. *Biophys J.* 2012;103(5):959-969.
2. Lomize AL, Schnitzer KA, Todd SC, et al. Membranome 3.0: Database of single-pass membrane proteins with AlphaFold models. *Protein Sci.* 2022;31(5):e4318.
